# Catalytic Intramolecular Aminofluorination, Oxyfluorination, and Carbofluorination with a Stable and Versatile Hypervalent Fluoroiodine Reagent**

**DOI:** 10.1002/anie.201503373

**Published:** 2015-06-09

**Authors:** Weiming Yuan, Kálmán J Szabó

**Affiliations:** Department of Organic Chemistry, Stockholm University(Sweden)

**Keywords:** copper, cyclizations, fluorine, heterocycles, homogeneous catalysis

## Abstract

Application of a fluoroiodine analogue of the Togni reagent was studied in fluorocyclization reactions. In the presence of a transition-metal catalyst the applied fluoroiodine reagent can be used for aminofluorination, oxyfluorination, and carbofluorination reactions. The described procedure has a very broad synthetic scope for preparation of functionalized hetero- and isocyclic compounds having a tertiary fluorine substituent.

Organofluorines are very important substances in the pharmaceutical industry, agrochemistry, and medical diagnostics.[1] The widespread use of organofluorines (including compounds with ^18^F isotopes) in life science related applications is due to their favorable biological, pharmacological, and radiochemical properties.[1] Therefore, there is a large demand for easy access to a broad variety of these species, which has been one of the most important driving forces for the development of new synthetic procedures for the preparation of organofluoro compounds.[2]

Recently, the progress in this field was also advanced by the appearance of new, safe, and easily accessible reagents even for electrophilic fluorination reactions. Although excellent electrophilic fluorination reagents have appeared previously, such as Selectfluor, NFSI, DAST, etc., the demand from important application areas of organofluorines motivates additional development of the reagents.[1–3] For example, ^18^F-labelling-based methodologies (such as PET scanning) requires the late-stage introduction of the fluorine to the reagent and subsequently to the target molecule.[1d, 2a, 3]

Electrophilic fluorocyclization reactions represent a very important methodology for the synthesis of heterocycles and functionalized carbocyclic compounds. After the pionneering studies of Liu and co-workers,[4] aminofluorination has become a very important method for the synthesis of nitrogen-containing heterocycles.[5] Most of the published procedures are suitable for the synthesis of piperazine derivatives (six-membered ring) but fluoro indoles/pyrroles and azepanes have also been reported.[4, 6] Fluorocyclization reactions for the synthesis of oxygen-containing rings have also been an attractive synthetic method for the introduction of the fluorine functionality into organic molecules.[6c,m, 7] A somewhat less developed but very challenging area involves carbofluorination reactions for the synthesis of fluorine-containing isocyclic compounds.[8] A limited number of reports have also been published on intermolecular aminofluorination[9] and carbofluorination[10] reactions.

Relatively few studies have been reported for the application of the same reagent under similar reaction conditions to perform all three types of cyclization reactions. There are a couple of reports for the application of intramolecular amino- and oxyfluorination methods based on similar amine and alcohol precursors.[6c,m] However, as far as we know all three types of fluorocyclizations (including even carbofluorination) of similar substrates using the same fluorination reagent have not been reported. The dominant fluorination reagents in the above procedures are NFSI and Selectfluor. The direct application of fluoroiodines is rather limited, because of the low stability and high reactivity of ArIF_2_ and related reagents.[6d, 7h] However, a couple of examples have been presented[4, 6h–j] for using stable and easily available acetyliodines [such as PhI(OAc)_2_, PhI(OPiv)_2_, PhI(TFA)_2_] and PhIO in combination with various fluorine sources for in situ generation of fluoroiodines.

As a concept-driven approach in our fluorine chemistry program,[11] we decided to investigate the application of the air-, moisture-, and thermostable fluoroiodine **1** (for structure see Figure 1) as a reagent for fluorination reactions.[11a] This reagent is a structural analogue of the Togni reagents,[2b, 12] which have been a very popular electrophilic trifluoromethylating reagents in organic synthesis.[2b,d,e] We expected that conceptual analogies between **1** and its CF_3_ analogue could be exploited for the development of new catalytic fluorination reactions. For example, catalytic oxytrifluoromethylation[11c,d, 13] and aminotrifluoromethylation[14] with the Togni reagent are well-known methods, and therefore it was appealing to attempt oxyfluorination and aminofluorination reactions with **1**. Furthermore, unlike Ar-IF_2_ derivatives, **1** is a stable and easily accessible reagent,[15] which is a potent oxidant, a fluorine source, and a preformed base in one reagent. A further interesting property is that **1** can be prepared from its chloro analogue by addition of KF. Thus, it satisfies an important criterion for late-stage electrophilic fluorinating reagents, namely that **1** can be easily prepared from simple anionic fluoride salts. This property can be very useful for ^18^F-labelling studies, as for example the synthesis of PET ligands.[3] So far relatively few studies have appeared on the application of **1** as a fluorinating reagent. This small number of reports is probably because its first synthesis was reported just a couple of years ago by Legault and Prévost.[15a] Stuart and co-workers[15c, 16] reported that **1** reacts with 1,3-diketoesters and 1,3-diketones in the presence of TREAT-HF to give mono- or difluoro products. In addition, recently, we have shown[11a] that **1** can be employed for the difluorination of styrene derivatives.

We have now found that **1** is an excellent and versatile reagent for fluorocyclization reactions in the presence of metal catalysts (Figure 1). Furthermore, **1** is suitable for amino-, oxy-, and carbofluorination of analogue substrates under very similar reaction conditions. The reagent **1** could be easily used for the synthesis of tertiary fluorides, which is often challenging with the commonly used reagents because of steric issues.

**Figure 1:**
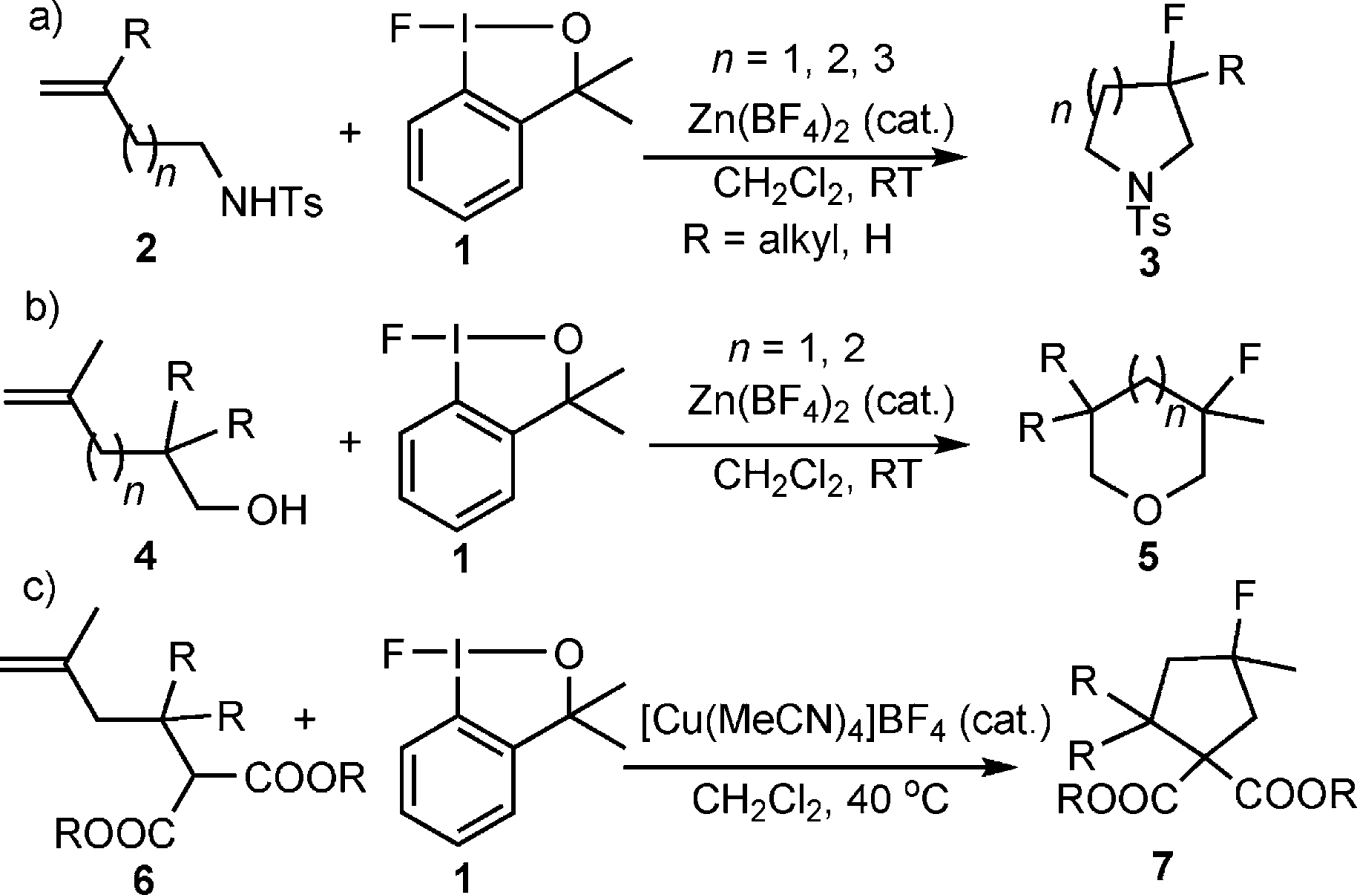
Catalytic fluorocyclization reactions in this study. Ts= 4-toluenesulfonyl.

We selected the fluorocyclization of the alkene tosylamide **2 a** by **1** as a model reaction (Table 1). Several metal catalysts, such as Cu, Pd, Ag, and Zn (tetrafluoroborate salt), proved to be useful catalysts for obtaining **3 a** with a tertiary fluoride substituent (entries 1–4 and 6). CuCl was inefficient (entry 5) and CH_2_Cl_2_ was the best solvent for the reaction (entries 6–9). We could not observe any reaction, when either **1** (entry 10) or the metal catalyst (entry 11) was omitted. The best results were obtained with the commercially available Zn(BF_4_)_2_ catalyst (5 mol %), which contains crystal water (entry 6). This trace amount of water did not affect (at least) the aminocyclization reaction.

**Table 1:** Development of the catalytic aminofluorination reaction^[a]^ 
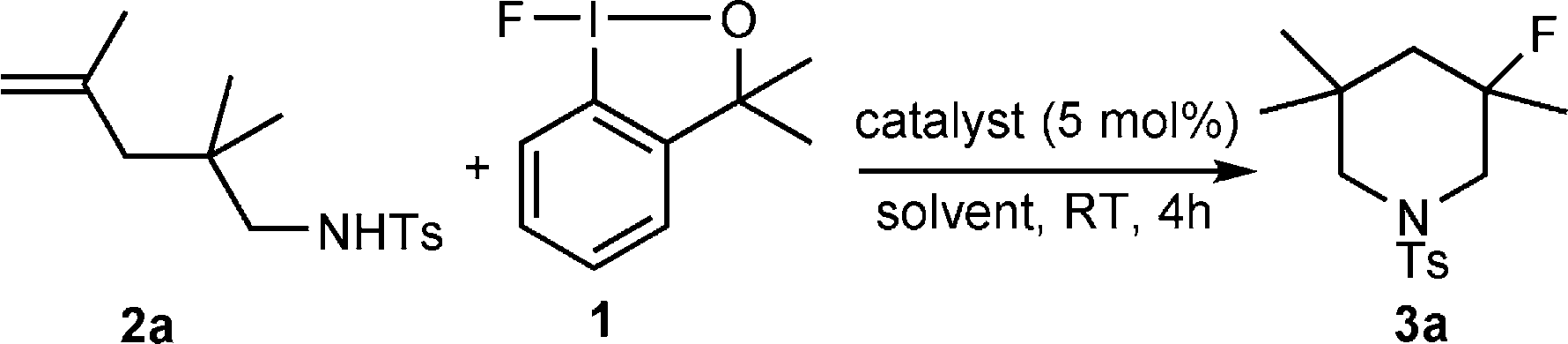

Entry	Catalyst	Solvent	Yield [%]^[b]^
1	[Cu(MeCN)_4_]BF_4_	CH_2_Cl_2_	65
2	[Pd(MeCN)_4_](BF_4_)_2_	CH_2_Cl_2_	50
3	[Ag(MeCN)_4_]BF_4_	CH_2_Cl_2_	46
4	AgBF_4_	CH_2_Cl_2_	58
5	CuCl	CH_2_Cl_2_	0
*6*	*Zn(BF_4_)_2_⋅x H_2_O*	*CH_2_Cl_2_*	*75*
7	Zn(BF_4_)_2_⋅*x* H_2_O	THF	40
8	Zn(BF_4_)_2_⋅*x* H_2_O	PhCH_3_	63
9	Zn(BF_4_)_2_⋅*x* H_2_O	CH_3_CN	<5
10^[c]^	Zn(BF_4_)_2_⋅*x* H_2_O	CH_2_Cl_2_	0
11	–	CH_2_Cl_2_	0

[a] Reaction conditions: 0.1 mmol of **2 a**, Zn(BF_4_)_2_⋅XH_2_O (5 mol %) and **1** (1.1 equiv) was reacted in CH_2_Cl_2_ (0.5 mL) at RT. [b] Yield of the isolated product. [c] Fluoroiodine **1** was not added. THF=tetrahydrofuran.

With the optimization results in hand, we studied the synthetic scope of the aminocyclization reaction in detail (Table 2). The reaction of **2** with **1** in the presence of Zn(BF_4_)_2_ proceeds under mild reaction conditions at room temperature without any additives. The typical reaction time was 3 hours. The reaction was very clean, thus affording high yields for different substituent patterns. The dimethyl-substituted **2 a** (entry 1) reacted about as fast as its diphenyl analogue **2 b** (entry 2), thus affording the tertiary fluoro piperidines **3 a** and **3 b**, respectively. The tosyl group could be replaced with mesyl (entry 3) or nosyl (entry 4) without affecting the reaction rate or lowering the yield. The cyclohexyl derivative **2 e** gave the spiro-piperidine derivative **3 e** in high yield (entry 5). The reaction proceeds well without a methyl substituent on the alkene group (**2 f**), thus affording the secondary fluoro piperidine derivative **3 f** (entry 6). Conversely, either an ethyl (**2 g**) or pentyl group (**2 h**), instead of methyl, on the alkene gave the crowded tertiary fluorines **3 g** and **3 h**, respectively (entries 7 and 8). The achievement of high stereoselectivity for tertiary fluorides is usually challenging. Cyclization of **2 i** to **3 i** (entry 9) proceeds with a poor stereoselectivity (d.r.=2:1), which might be due to the relatively remote stereocenters. Gratifyingly, cyclization of **2 j** (in which the stereocenters are in adjacent position) afforded **3 j** (entry 10) in high stereoselectivity (d.r.>10:1). The double bond in **2 j** is at an internal position, thus indicating that the fluorocyclization does not necessarily require terminal alkenes. However, the aminocyclization was slower for **2 j** (9 h) than for terminal alkenes (typically 3 h). When we employed **2 k** (the *Z* isomer of **2 j**) the reaction resulted in **3 k** (the diastereomeric form of **3 j**) with high diastereoselectivity (entry 11). The cyclization of **2 k** had to be performed at 40 °C (instead of RT), as this substrate is probably more sterically hindered than **2 j**. We have also studied the cyclization of tosyl amide alkenes with different tethers (entries 12–14) to explore the possibility for variation of the ring size. The compound **2 l** (entry 12) gave the pyrrole derivative **3 l**, and proceeded more slowly than the reaction of **2 m**, thus leading to the piperidine derivative **3 m** (entry 13). However, the yields for the two processes were about the same. Azepane with a tertiary fluoro substituent (**3 n**) also formed easily and in high yield from **2 n**. In this process formation of smaller ring sizes (such as **3 l**–**n**) was not observed.

**Table 2:** Catalytic aminofluorination with 1^[a]^

Entry	Substrate	*t* [h]	Product	Yield [%]^[b]^
1	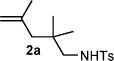	3	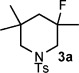	75
2	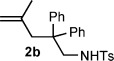	3	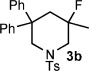	73
3	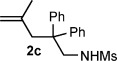	3	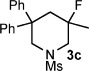	82
4	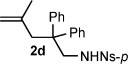	3	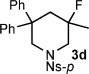	84
5	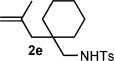	3	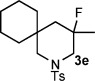	84
6	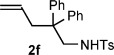	3	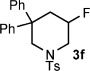	71
7	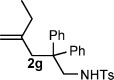	3	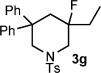	72
8	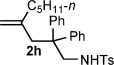	4	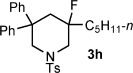	62
9	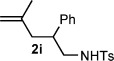	3	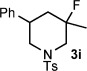	62 (2:1)^[c]^
10^[d]^	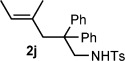	9	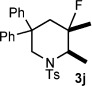	63 (>10:1)^[c]^
11^[e]^	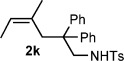	5	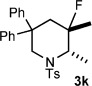	65 (>10:1)^[c]^
12	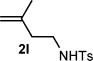	6	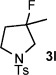	70
13	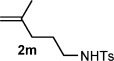	3	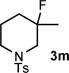	73
14	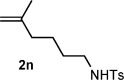	4	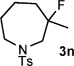	70

[a] 0.3 mmol of **2**, Zn(BF_4_)_2_⋅*x* H_2_O (5 mol %) and **1** (1.1 equiv) was reacted in CH_2_Cl_2_ (0.5 mL) at RT. [b] Yield of isolated product. [c] The ratio of diastereomers was determined by ^19^F NMR analysis of the crude reaction mixture. [d] Zn(BF_4_)_2_⋅*x* H_2_O (10 mol %) in CH_2_Cl_2_ (0.3 mL) was used. [e] The reaction was performed at 40 °C. Ms=methanesulfonyl, Ns=4-nitrobenzenesulfonyl.

Subsequently, we studied the possibility for extending the fluorocyclization reaction to the synthesis of oxygen-containing heterocycles and isocyclic compounds (Table 3). The alkenyl alcohols **4 a**–**c** (entries 1–3) could also be cyclized with **1** and Zn(BF_4_)_2_ under identical reaction conditions to those used for the amino derivatives **2 a**–**n**. In fact the reaction times were shorter (typically 1–2 h) for the oxyfluorination process than for the aminofluorination reaction (typically 3 h). The reaction was suitable for formation of both six- and seven-membered heterocycles (entries 1–3). In these processes only tertiary fluorides were formed. The regioselectivity was the same as for the aminofluorination, thus affording the *endo*-cyclized pyrane (entries 1 and 2) and oxepane (entry 3) products exclusively. To our delight, the cyclization could be extended to carbofluorination reactions as well (entries 4–7). Zn(BF_4_)_2_ was not suitable as a catalyst, probably because it contained crystal water. However, [Cu(MeCN)_4_]BF_4_ (10 mol %) proved to be an excellent catalyst for carbocyclization of the alkenyl malonate derivatives **6 a**–**d**. The process results exclusively in cyclopentane derivatives having a tertiary fluorine substituent (**7 a**–**d**). The reaction requires longer reaction times (typically 8 h) than the cyclization of the amino and hydroxy analogues (typically 1–3 h). The longer reaction time is apparently required because of the poor nucleophilicity of the malonate carbon atom in **6** compared to the nitrogen and oxygen atoms in **2** and **4**, respectively. The ethyl malonate **6 b** gave a somewhat higher yield than the methyl malonate **6 a** (entries 4 and 5). The reaction proceeds smoothly, even for the densely substituted substrate **6 c**, which gave a cyclopentane derivative **7 c** having three quaternary carbon atoms. The substrate **6 d** underwent facile cyclization to give **7 d** with poor stereoselectivity (entry 7). The poor stereoselectivity can be explained by the remote position of the stereocenters and presence of the bulky carboxylate groups.

**Table 3:** Fluorocyclization by oxy- and carbofluorination using 1^[a]^

Entry	Substrate	Method	*t* [h]	Product	Yield [%]^[b]^
1	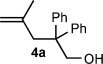	A	2	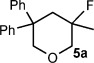	62
2	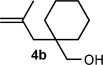	A	1	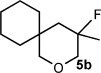	65
3	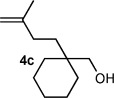	A	1	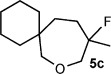	60
4	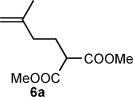	B	8	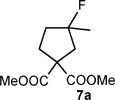	55
5	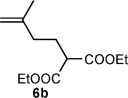	B	8	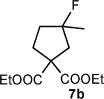	60
6^[c]^	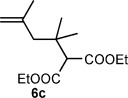	B	8	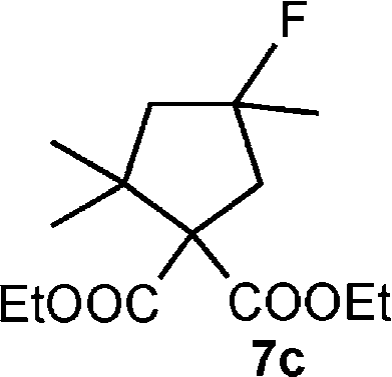	76
7	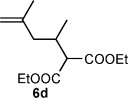	B	4	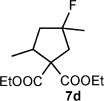	60 (2:1)^[d]^

[a] Method A: 0.3 mmol of **4**, Zn(BF_4_)_2_⋅*x* H_2_O (5 mol %) and **1** (1.1 equiv) was reacted in CH_2_Cl_2_ (0.5 mL) at RT; Method B: 0.3 mmol of **6**, [Cu(MeCN)_4_]BF_4_ (10 mol %) and **1** (1.5 equiv) was reacted in CH_2_Cl_2_ (0.5 mL) at 40 °C. [b] Yield of the isolated product. [c] The reaction was performed at RT. [d] The ratio of diastereomers was determined by ^19^F NMR analysis of the crude reaction mixture.

Based on the above results we propose a mechanism for the fluorocyclization reactions in Figure 2. For the clarity the mechanism is given for the cyclization of **2 m** to **3 m**, which probably can be extended to the other substrates as well. As mentioned above the reactions cannot be performed without a metal catalyst (Table 1, entry 11). Therefore, we suggest that the metal catalyst, such as Zn(BF_4_)_2_, activates **1** to form **8**. A similar type of activation of the CF_3_ analogue using Zn salts was reported by Togni and co-workers.[17] In the activated complex **8** the hypervalent iodine has a low-lying empty orbital (formed by cleavage of the iodine–oxygen bond), which is easily accessible for the π electrons in the double bond of **2 m**. Thus electrophilic addition of **8** to **2 m** leads to the formation of the iodonium ion **9**.[11a] Formation of a similar type of iodonium ion has been invoked for reactions of Ar-IF_2_ reagents and its analogues.[6h, 7h] The next step is probably a nucleophilic attack at the least hindered corner of the iodocyclopropylium cation in **9** to give **10**. This step proceeds with very high regioselectivity, as all the presented reactions give only one regioisomer of the cyclic product. The next step can be nucleophilic displacement of the hypervalent iodine by the fluoride ion to give a tertiary fluoride functionality. The alkoxide moiety in **10** probably deprotonates the nitrogen atom to give the product **3 m**, **11**, and regenerates the catalyst. In the case of using Zn(BF_4_)_2_⋅*x* H_2_O as the catalyst, the water may mediate the proton transfer from the substrate to the alkoxide moiety. However, when [Cu(MeCN)_4_]BF_4_ is used as the catalyst, the alkoxide in **1** may directly deprotonate the substrate (Table 1, entry 1 and Table 3 entries 4–7). Alternatively, the nucleophilic attack by the fluoride may follow the deprotonation of the nitrogen atom. The high basicity of the alkoxide moiety in **10** is probably one of the main reasons for the broad synthetic scope of the reactions. In particular, the successful achievement of the carbocyclization of **6 a**–**d** may be due to the presence of a highly efficient internal base. Furthermore, the alkoxide moiety is very bulky, therefore it cannot compete with the tosyl amide (alcohol moiety in **4** or the malonate in **6**) in the nucleophilic attack of the iodocyclopropylium cation in **9**. Formation of this bulky alkoxide is an inherent property of **1** and its activation mechanism. When other hypervalent iodines are used for fluorocyclizations, the internally formed base is often acetoxy or a halogenide ion, which are usually less efficient for deprotonation than the tertiary alkoxide ion generated from **1**.

**Figure 2:**
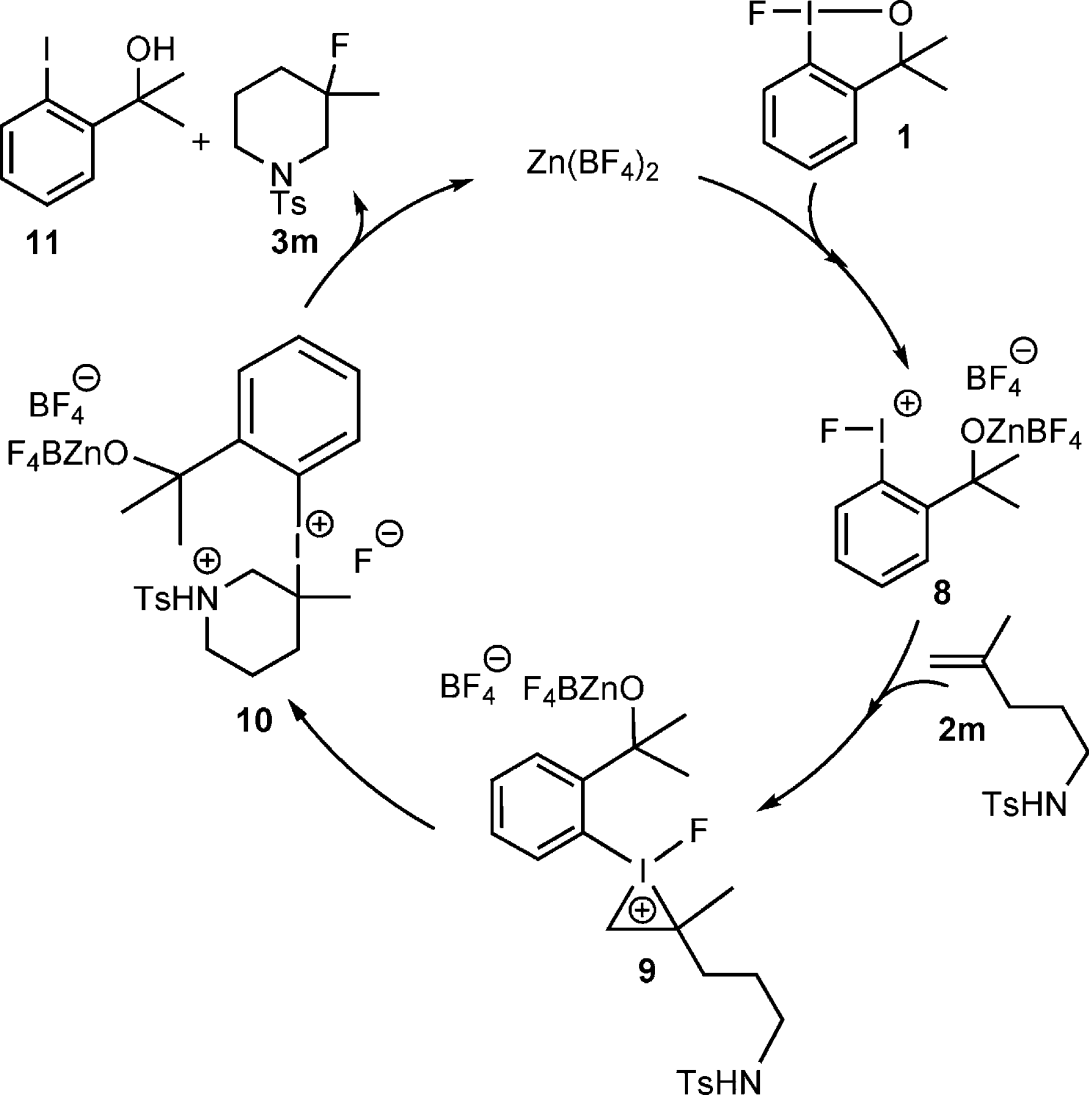
Proposed mechanism of the fluorocyclization reactions exemplified by the reaction of 2 m to afford 3 m (Table 2, entry 13).

In summary, we have shown that **1** is an efficient reagent for fluorocyclization-based aminofluorination, oxyfluorination, and carbofluorination. The processes are suitable for the synthesis of hetero- and isocycles having tertiary and secondary fluorine substituents. By using this method functionalized pyrrole, piperidine, azepane, pyrane, oxepane, and cyclopentane derivatives can be synthesized. The reaction requires activation of **1** by transition metals, such as Zn, Cu, Ag, or Pd. The broad synthetic scope of the reaction probably also depends on the in situ generation of an alkoxide base, which ensures an efficient deprotonation in the nucleophilic step of the process.

## Experimental Section

In a typical procedure (Method A) 0.3 mmol of either **2** or **4**, Zn(BF_4_)_2_⋅*x* H_2_O (5 mol %), and **1** (1.1 equiv) were stirred in CH_2_Cl_2_ (0.5 mL) at RT for three hours. Then the solvent was removed and the product was purified by chromatography. The carbocyclization of the malonate derivatives (**6**) was performed in a similar way (Method B), except that 0.3 mmol of **6**, [Cu(MeCN)_4_]BF_4_ (10 mol %), and **1** (1.5 equiv) were reacted at 40 °C for 8 h.

## Supporting Information

As a service to our authors and readers, this journal provides supporting information supplied by the authors. Such materials are peer reviewed and may be re-organized for online delivery, but are not copy-edited or typeset. Technical support issues arising from supporting information (other than missing files) should be addressed to the authors.

miscellaneous_information
