# Supplementary material for: Catalytic Intramolecular Aminofluorination, Oxyfluorination, and Carbofluorination with a Stable and Versatile Hypervalent Fluoroiodine Reagent
Source: Angew Chem Int Ed Engl. 2015 Jun 9;54(29):8533–7. doi: 10.1002/anie.201503373 (PMC4531827; doi:10.1002/anie.201503373)
Supplement: Supplementary file 1 — miscellaneous_information [file anie0054-8533-sd1.pdf]

Angewandte Chemie
Eine Zeitschrift der Gesellschaft Deutscher Chemiker

Supporting Information

Catalytic Intramolecular Aminofluorination, Oxyfluorination, and Carbofluorination with a Stable and Versatile Hypervalent Fluoroiodine Reagent**

*Weiming Yuan and Kálmán J. Szabó**

anie_201503373_sm_miscellaneous_information.pdf

Contents:

General Information 2

Experimental Procedures and Spectral Data 2

References 13

NMR spectra of products 3a-3n, 5a-5c and 7a-7d 14

General Information

Hypervalent iodine **1**[1] and alkenes[2] were prepared according to literature procedures. All the other chemicals were obtained from commercial sources and used as received. ^{1}H NMR, ^{13}C NMR and ^{19}F NMR spectra were recorded in $CDCl_3$ (internal standard: 7.26 ppm, ^{1}H; 77.0 ppm, ^{13}C) using 400 MHz spectrometers. High resolution mass data (HRMS) were obtained using ESI technique. For column chromatography, silica gel (35-70 microns) was used. Unless otherwise stated the reactions were conducted under argon.

Experimental Procedures and Spectral Data

Method A for catalytic fluorocyclization

Fluoroiodine reagent **1** (92.0 mg, 0.33 mmol), the corresponding alkenes **2** (0.3 mmol), $Zn(BF_4)_2XH_2O$ (3.6 mg, 0.015 mmol) were mixed in DCM (0.5 mL). This reaction mixture was stirred at room temperature. After completion of the reaction, the resulting mixture was concentrated and purified by column chromatography on silca gel.

Method B for catalytic fluorocyclization

Fluoroiodine reagent **1** (126.0 mg, 0.45 mmol), the corresponding alkenes **2** (0.3 mmol), $Cu(MeCN)_4BF_4$ (9.4 mg, 0.03 mmol) were mixed in DCM (0.5 mL). This reaction mixture was stirred at 40 ^{o}C. After completion of the reaction, the resulting mixture was concentrated and purified by column chromatography on silca gel.

3-Fluoro-3,5,5-trimethyl-1-tosylpiperidine (3a) [3]

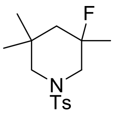

This compound was prepared according to method A. Compound **3a** was isolated as a white solid by chromatography using pentane:Et_2O (5:1) eluent system (67.4 mg, 75%). ^{1}H-NMR (400 MHz, $CDCl_3$) δ 0.90 (s, 3H); 1.08-1.23 (m, 4H); 1.30 (d, J = 20.6 Hz, 3H); 1.66-1.79 (m, 1H); 2.12 (d, J = 11.5 Hz, 1H); 2.32 (dd, $J_{H\text{-}F}$ = 31.6 Hz, $J_{H\text{-}H}$ = 12.6 Hz, 1H); 2.42 (s, 3H); 3.31 (dt, J = 11.6, 1.9 Hz, 1H); 3.67-3.76 (m, 1H); 7.31 (d, J = 8.2 Hz, 2H); 7.65 (d, J = 8.4 Hz, 2H); ^{19}F-NMR (376 MHz, $CDCl_3$) δ -145.00 (br); ^{13}C-NMR (100 MHz, $CDCl_3$) δ 21.5; 25.6 (d, J_{CF} = 14.7 Hz); 25.7 (d, J_{CF} = 15.4 Hz); 29.0; 31.1; 46.8 (d, J_{CF} = 21.0 Hz); 53.9 (d, J_{CF} = 23.5 Hz); 56.5; 91.2 (d, J_{CF} = 176.8 Hz); 127.5; 129.6; 134.0; 143.4; HRMS (ESI): m/z calcd. for $[C_{15}H_{22}FNNaO_2S]^+$ 322.1247, found 322.1247; Mp: 130-131 ^{o}C.

The spectral data is in agreement with the literature values of ref [3].

5-Fluoro-5-methyl-3,3-diphenyl-1-tosylpiperidine (3b) [3]

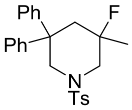

This compound was prepared according to method A. Compound **3b** was isolated as a white solid by chromatography using pentane:Et_2O (5:1) eluent system (92.8 mg, 73%). ^{1}H-NMR (400 MHz, $CDCl_3$) δ 1.17 (d, J = 22.8 Hz, 3H); 2.45 (s, 3H); 2.49-2.63 (m, 1H); 2.70 (t, J = 13.2 Hz, 1H); 3.02 (t, J = 10.3 Hz, 1H); 3.08-3.20 (m, 1H); 3.43 (d, J = 12.7 Hz, 1H); 3.71 (d, J = 12.6 Hz, 1H); 7.13-7.25 (m, 2H); 7.26-7.46 (m, 10H); 7.70 (d, J = 8.3 Hz, 2H); ^{19}F-NMR (376 MHz, $CDCl_3$) δ -137.98 (br); ^{13}C-NMR (100 MHz, $CDCl_3$) δ 21.5; 25.1 (d, J_{CF} = 24.2 Hz); 44.9 (d, J_{CF} = 20.7 Hz); 45.5 (d, J_{CF} = 6.0 Hz); 53.8; 54.6 (d, J_{CF} = 29.4 Hz); 91.5 (d, J_{CF} = 174.6 Hz); 126.2; 126.4; 126.8; 127.1; 127.7; 128.3; 128.4; 129.8; 132.3; 143.9; 145.1; 145.4; HRMS (ESI): m/z calcd. for $[C_{25}H_{26}FNNaO_2S]^+$ 446.1560, found 446.1571; Mp: 160-162 ^{o}C.

The spectral data is in agreement with the literature values of ref [3].

5-Fluoro-5-methyl-1-(methylsulfonyl)-3,3-diphenylpiperidine (3c)

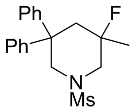

This compound was prepared according to method A. Compound **3c** was isolated as a white solid by chromatography using pentane:Et_2O (3:1) eluent system (85.5 mg, 82%). ^{1}H-NMR (400 MHz, $CDCl_3$) δ 1.28 (d, *J* = 22.1 Hz, 3H); 2.50 (dd, *J* = 23.8, 14.1 Hz, 1H); 2.67 (s, 3H); 2.90-3.06 (m, 1H); 3.25 (dd, *J* = 20.4, 12.5 Hz, 1H); 3.43 (t, *J* = 11.4 Hz, 1H); 3.78 (d, *J* = 13.0 Hz, 1 H); 4.08 (d, *J* = 13.1 Hz, 1H); 7.16-7.45 (m, 10H); ^{19}F-NMR (376 MHz, $CDCl_3$) δ -139.61 (br); ^{13}C-NMR (100 MHz, $CDCl_3$) δ 25.0 (d, J_{CF} = 23.5 Hz); 35.9; 44.6 (d, J_{CF} = 21.0 Hz); 45.6 (d, J_{CF} = 4.4 Hz); 53.6; 54.4 (d, J_{CF} = 27.5 Hz); 91.4 (d, J_{CF} = 175.2 Hz); 126.3; 126.5; 126.6; 127.1; 128.2; 128.5; 144.7; 145.4; HRMS (ESI): *m*/*z* calcd. for $[C_{19}H_{22}FNNaO_2S]^+$ 370.1247, found 370.1255; Mp: 153-154 ^{o}C.

5-Fluoro-5-methyl-3,3-diphenyl-1-(*p*-nitro-benzenesulfonyl)piperidine (3d)

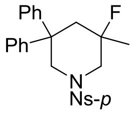

This compound was prepared according to method A. Compound **3d** was isolated as a white solid by chromatography using pentane:Et_2O (3:1) eluent system (114.4 mg, 84%). ^{1}H-NMR (400 MHz, $CDCl_3$) δ 1.22 (d, *J* = 22.1 Hz, 3H); 2.47 (dd, *J* = 21.5, 14.1 Hz, 1H); 2.73-2.87 (m, 1H); 3.02-3.27 (m, 2H); 3.56-3.80 (m, 2H); 7.18-7.37 (m, 10H); 7.95 (d, *J* = 8.8 Hz, 2H); 8.35 (d, *J* = 8.9 Hz, 2H); ^{19}F-NMR (376 MHz, $CDCl_3$) δ -139.39 (br); ^{13}C-NMR (100 MHz, $CDCl_3$) δ 25.1 (d, J_{CF} = 23.5 Hz); 44.6 (d, J_{CF} = 20.5 Hz); 45.6 (d, J_{CF} = 5.2 Hz); 53.7; 54.4 (d, J_{CF} = 28.7 Hz); 91.1 (d, J_{CF} = 176.2 Hz); 124.4; 126.4; 126.7; 126.8; 126.9; 128.4; 128.6; 128.9; 141.7; 144.7; 145.0; 150.2; HRMS (ESI): *m*/*z* calcd. for $[C_{24}H_{23}FN_2NaO_4S]^+$ 477.1255, found 477.1247; Mp: 180-182 ^{o}C.

5-Cyclohexyl-3-fluoro-3-methyl-1-tosylpiperidine (3e)

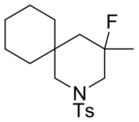

This compound was prepared according to method A. Compound **3e** was isolated as a white solid by chromatography using pentane:Et_2O (5:1)

eluent system (85.5 mg, 84%). ^{1}H-NMR (400 MHz, $CDCl_3$) δ 0.97-1.15 (m, 1H); 1.15-1.23 (m, 2H); 1.30 (d, J = 20.5 Hz, 3H); 1.35-1.63 (m, 7H); 1.69-1.80 (m, 1H); 1.84-1.95 (m, 1H); 2.06 (d, J = 11.9 Hz, 1H); 2.29-2.47 (m, 4H); 3.57-3.67 (m, 1H); 3.68-3.79 (m, 1H); 7.32 (d, J = 8.3 Hz, 2H); 7.67 (d, J = 8.3 Hz, 2H); ^{19}F-NMR (376 MHz, $CDCl_3$) δ -143.41 (m); ^{13}C-NMR (100 MHz, $CDCl_3$) δ 21.4; 21.5; 25.9 (d, J_{CF} = 24.3 Hz); 26.1; 33.1; 33.2; 33.7; 37.7; 45.4 (d, J_{CF} = 20.8 Hz); 53.8; 54.5 (d, J_{CF} = 24.3 Hz); 91.5 (d, J_{CF} = 176.2 Hz); 127.5; 129.6; 134.2; 143.4; HRMS (ESI): *m/z* calcd. for $[C_{18}H_{26}FNNaO_2S]^+$ 362.1560, found 362.1569; Mp: 114-115 ^{o}C.

5-Fluoro-3,3-diphenyl-1-tosylpiperidine (3f)[3]

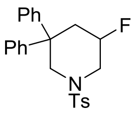

This compound was prepared according to method A. Compound **3f** was isolated as a white solid by chromatography using pentane:Et_2O (5:1) eluent system (87.2 mg, 71%). ^{1}H-NMR (400 MHz, $CDCl_3$) δ 2.14-2.25 (m, 1H); 2.28-2.38 (m, 1H); 2.39-2.55 (m, 4H); 2.91-3.07 (m, 1H); 3.99-4.15 (m, 1H); 4.43-4.70 (m, 2H); 7.13-7.44 (m, 10H); 7.50 (d, J = 7.8 Hz, 2H); 7.68 (d, J = 8.1 Hz, 2H); ^{19}F-NMR (376 MHz, $CDCl_3$) δ -185.46 (d, J = 47.9 Hz); ^{13}C-NMR (100 MHz, $CDCl_3$) δ 21.5; 41.0 (d, J_{CF} = 18.5 Hz); 46.5 (d, J_{CF} = 11.0 Hz); 49.9 (d, J_{CF} = 31.2 Hz); 53.8; 85.6 (d, J_{CF} = 173.9 Hz); 126.4; 126.5; 126.9; 127.69; 127.73; 128.58; 128.66; 129.9; 132.1; 143.1; 144.0; 145.4; HRMS (ESI): *m/z* calcd. for $[C_{24}H_{24}FNNaO_2S]^+$ 432.1404, found 432.1398; Mp: 167-168 ^{o}C.

The spectral data is in agreement with the literature values of ref [3].

5-Ethyl -5-fluoro-3,3-diphenyl-1-tosylpiperidine (3g)

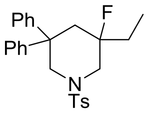

This compound was prepared according to method A. Compound **3g** was isolated as a white solid by chromatography using pentane:Et_2O (5:1) eluent system (94.5 mg, 72%). ^{1}H-NMR (400 MHz, $CDCl_3$) δ 0.82 (t, J =

7.4 Hz, 3H); 1.34-1.54 (m, 2H); 2.36-2.57 (m, 4H); 2.61-2.75 (m, 1H); 2.98-3.17 (m, 2H); 3.47-3.66 (m, 2H); 7.13-7.24 (m, 2H); 7.25-7.43 (m, 10H); 7.69 (d, J = 8.3 Hz, 2H); ^{19}F-NMR (376 MHz, $CDCl_3$) δ -151.29 (m); ^{13}C-NMR (100 MHz, $CDCl_3$) δ 6.8 (d, J_{CF} = 5.1 Hz); 21.5; 30.4 (d, J_{CF} = 22.2 Hz); 42.8 (d, J_{CF} = 20.6 Hz); 45.4 (d, J_{CF} = 5.9 Hz); 52.7 (d, J_{CF} = 28.7 Hz); 54.1; 93.0 (d, J_{CF} = 177.8 Hz); 126.2; 126.4; 126.9; 127.1; 127.7; 128.2; 128.4; 129.8; 132.4; 143.9; 145.47; 145.53; HRMS (ESI): *m*/*z* calcd. for $[C_{26}H_{28}FNNaO_2S]^+$ 460.1717, found 460.1716; Mp: 168-169 °C.

5-Fluoro-5-pentyl-3,3-diphenyl-1-tosylpiperidine (3h)

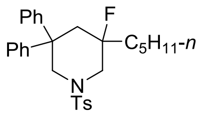

This compound was prepared according to method A. Compound **3h** was isolated as a white solid by chromatography using pentane:Et_2O (5:1) eluent system (89.1 mg, 62%). ^{1}H-NMR (400 MHz, $CDCl_3$) δ 0.85 (t, J = 7.3 Hz, 3H); 0.95-1.52 (m, 8H); 2.39-2.73 (m, 5H); 2.95-3.22 (m, 2H); 3.43-3.71 (m, 2H); 7.12-7.44 (m, 12H); 7.70 (d, J = 8.2 Hz, 2H); ^{19}F-NMR (376 MHz, $CDCl_3$) δ -148.59 (br); ^{13}C-NMR (100 MHz, $CDCl_3$) δ 13.9; 21.5; 22.0 (d, J_{CF} = 3.7 Hz); 22.3; 31.7; 37.6 (d, J_{CF} = 22.1 Hz); 42.7 (d, J_{CF} = 20.5 Hz); 45.4 (d, J_{CF} = 5.9 Hz); 53.4 (d, J_{CF} = 29.1 Hz); 54.1; 93.0 (d, J_{CF} = 177.4 Hz); 126.2; 126.4; 126.9; 127.1; 127.7; 128.2; 128.4; 129.8; 132.4; 143.9; 145.4; 145.6; HRMS (ESI): *m*/*z* calcd. for $[C_{29}H_{34}FNNaO_2S]^+$ 502.2186, found 502.2172; Mp: 190-191 °C.

5-Fluoro-5-methyl-3-phenyl-1-tosylpiperidine (3i)

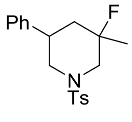

This compound was prepared according to method A. Compound **3i** was isolated as a white solid by chromatography using pentane:Et_2O (5:1) eluent system (64.6 mg, 62%). Spectral data for the separated major diastereomer: ^{1}H-NMR (400 MHz, $CDCl_3$) δ 1.58 (d, J = 23.2 Hz, 3H); 1.65-1.84 (m, 1H); 1.99-2.14 (m, 2H); 2.28 (dd, J = 10.7, 6.8 Hz, 1H); 2.36 (s, 3H); 2.84-3.02 (m, 1H); 3.72-3.78 (m, 1H); 3.79-3.89 (m, 1H); 7.07-7.14 (m, 2H); 7.15-7.30 (m, 5H);

7.54 (d, J = 8.3 Hz, 2H); ^{19}F-NMR (376 MHz, $CDCl_3$) δ -138.50 (m); ^{13}C-NMR (100 MHz, $CDCl_3$) δ 21.5; 23.4 (d, J_{CF} = 24.2 Hz); 40.2 (d, J_{CF} = 11.2 Hz); 42.2 (d, J_{CF} = 21.3 Hz); 52.5; 54.2 (d, J_{CF} = 34.6 Hz); 92.3 (d, J_{CF} = 172.0 Hz); 127.1; 127.4; 127.5; 128.8; 129.8; 133.0; 140.4; 143.8; HRMS (ESI): *m*/*z* calcd. for $[C_{19}H_{22}FNNaO_2S]^+$ 370.1247, found 370.1247; Mp: 154-155 ^{o}C.

5-Fluoro-5,6-dimethyl-3,3-diphenyl-1-tosylpiperidine (3j)

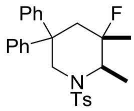

This compound was prepared according to method A. Compound **3j** was isolated as a white solid by chromatography using pentane:Et_2O (5:1) eluent system (82.7 mg, 63%). ^{1}H-NMR (400 MHz, $CDCl_3$) δ 1.15 (d, J = 7.0 Hz, 3H); 1.35 (d, J = 21.2 Hz, 3H); 2.29-2.50 (m, 4H); 2.85-2.96 (m, 1H); 3.39 (d, J = 13.6 Hz, 1H); 3.94 (dq, J_{H-F} = 13.3 J_{H-H} = 7.1 Hz, 1H); 4.73 (dd, J = 13.7, 1.7 Hz, 1H); 7.10-7.24 (m, 8H); 7.26-7.32 (m, 2H); 7.40 (d, J = 7.8 Hz, 2H); 7.62 (d, J = 8.3 Hz, 2H); ^{19}F-NMR (376 MHz, $CDCl_3$) δ -139.81 (m); ^{13}C-NMR (100 MHz, $CDCl_3$) δ 12.9 (d, J_{CF} = 8.1 Hz); 21.5; 25.4 (d, J_{CF} = 24.2 Hz); 40.0 (d, J_{CF} = 19.2 Hz); 45.3; 46.4; 56.3 (d, J_{CF} = 22.7 Hz); 94.0 (d, J_{CF} = 178.6 Hz); 125.6; 126.1; 126.5; 127.39; 127.42; 127.8; 128.6; 129.4; 136.6; 143.3; 144.9; 147.4; HRMS (ESI): *m*/*z* calcd. for $[C_{26}H_{28}FNNaO_2S]^+$ 460.1717, found 460.1722; Mp: 174-175 ^{o}C.

The stereochemistry was determined on the basis of the coupling constant between H(6) and F(5), which is 13.3 Hz. This coupling constant indicates a *cis* geometry for H(6) and F(5). In the case of *trans* geometry of H(6) and F(5) a larger coupling constant is expected (about 36 Hz, see ref [3] SI). Since H(6) and F(5) are *cis*, the two methyl groups Me(5) and Me(6) should also be in *cis* position. On the basis of this, the relative configuration of the Me(5) and Me(6) groups of **3k** was assigned as *trans*.

5-Fluoro-5,6-dimethyl-3,3-diphenyl-1-tosylpiperidine (3k)

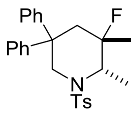

This compound was prepared according to method A. Compound **3k** was isolated as a white solid by chromatography using pentane:Et_2O (5:1) eluent system (85.3 mg, 65%). ^{1}H-NMR (400 MHz, $CDCl_3$) δ 0.85 (dd, *J* = 6.8, 3.0 Hz, 3 H); 0.91 (d, *J* = 25.1 Hz, 3H); 2.42 (s, 3 H); 2.56-2.65 (m, 1H); 2.78 (t, *J* = 13.7 Hz, 1H); 2.99 (d, *J* = 13.4 Hz, 1H); 4.00 (q, *J* = 6.84 Hz, 1H); 4.78 (d, *J* = 13.6 Hz, 1H); 7.15-7.24 (m, 4H); 7.25-7.34 (m, 6H); 7.58 (d, *J* = 7.8 Hz, 2H); 7.74 (d, *J* = 8.3 Hz, 2H); ^{19}F-NMR (376 MHz, $CDCl_3$) δ -134.11 (m); ^{13}C-NMR (100 MHz, $CDCl_3$) δ 8.3 (d, J_{CF} = 5.2 Hz); 21.5; 25.5 (d, J_{CF} = 24.4 Hz); 39.9 (d, J_{CF} = 22.9 Hz); 46.4; 46.6 (d, J_{CF} = 12.1 Hz); 56.2 (d, J_{CF} = 27.3 Hz); 93.6 (d, J_{CF} = 174.1 Hz); 126.1; 126.2; 126.7; 127.5; 127.6; 128.4; 128.7; 129.8; 136.8; 143.8; 144.4; 146.7; HRMS (ESI): *m*/*z* calcd. for $[C_{26}H_{28}FNNaO_2S]^+$ 460.1717, found 460.1714; Mp: 172-173 ^{o}C.

3-Fluoro-3-methyl-1-tosylpyrrolidine (3l) [4]

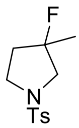

This compound was prepared according to method A. Compound **3l** was isolated as a white solid by chromatography using pentane:Et_2O (5:1) eluent system (54.0 mg, 70%). ^{1}H-NMR (400 MHz, $CDCl_3$) δ 1.45 (d, *J* = 20.5 Hz, 3H); 1.75-1.95 (m, 1H); 2.03-2.15 (m, 1H); 2.43 (s, 3H); 3.23-3.40 (m, 2H); 3.43-3.61 (m, 2H); 7.32 (d, *J* = 8.0 Hz, 2H); 7.71 (d, *J* = 8.4 Hz, 2H); ^{19}F-NMR (376 MHz, $CDCl_3$) δ -140.89 (m); ^{13}C-NMR (100 MHz, $CDCl_3$) δ 21.5; 21.8 (d, J_{CF} = 21.5 Hz); 37.7 (d, J_{CF} = 23.6 Hz); 46.9; 58.3 (d, J_{CF} = 25.7 Hz); 100.3 (d, J_{CF} = 176.0 Hz); 127.5; 129.6; 133.7; 143.6; HRMS (ESI): *m*/*z* calcd. for $[C_{12}H_{16}FNNaO_2S]^+$ 280.0778, found 280.0788; Mp: 105-107 ^{o}C.

The spectral data is in agreement with the literature values of ref [4].

3-Fluoro-3-methyl-1-tosylpiperidine (3m)

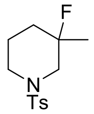

This compound was prepared according to method A. Compound **3m** was isolated as a white solid by chromatography using pentane:Et_2O (5:1) eluent system (59.4 mg, 73%). ^{1}H-NMR (400 MHz, $CDCl_3$) δ 1.32-1.54 (m, 4H); 1.55-1.65 (m, 1H); 1.72-1.92 (m, 2H); 2.43 (s, 3H); 2.66-2.83 (m, 2H); 3.23-3.41 (m, 2H); 7.32 (d, J = 8.0 Hz, 2H); 7.65 (d, J = 8.3 Hz, 2H); ^{19}F-NMR (376 MHz, $CDCl_3$) δ -148.97 (br); ^{13}C-NMR (100 MHz, $CDCl_3$) δ 21.2 (d, J_{CF} = 4.41 Hz); 21.5; 24.3 (d, J_{CF} = 23.5 Hz); 34.5 (d, J_{CF} = 22.7 Hz); 45.5; 53.9 (d, J_{CF} = 26.4 Hz); 90.6 (d, J_{CF} = 174.6 Hz); 127.6; 129.6; 133.7; 143.5; HRMS (ESI): m/z calcd. for $[C_{13}H_{18}FNNaO_2S]^+$ 294.0934, found 294.0949; Mp: 104-105 ^oC.

3-Fluoro-3-methyl-1-tosylazepane (3n)

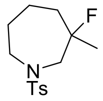

This compound was prepared according to method A. Compound **3n** was isolated as a white solid by chromatography using pentane:Et_2O (5:1) eluent system (59.9 mg, 70%). ^{1}H-NMR (400 MHz, $CDCl_3$) δ 1.46 (d, J = 21.9 Hz, 3H); 1.51-2.01 (m, 6H); 2.42 (s, 3H); 2.86-3.00 (m, 1H); 3.15-3.28 (m, 1H); 3.42-3.56 (m, 2H); 7.30 (d, J = 8.2 Hz, 2H); 7.67 (d, J = 8.3 Hz, 2H); ^{19}F-NMR (376 MHz, $CDCl_3$) δ -139.15 (m); ^{13}C-NMR (100 MHz, $CDCl_3$) δ 21.0 (d, J_{CF} = 5.2 Hz); 21.5; 26.1 (d, J_{CF} = 24.3 Hz); 30.0; 39.1 (d, J_{CF} = 23.5 Hz); 50.9; 57.5 (d, J_{CF} = 33.8 Hz); 96.9 (d, J_{CF} = 170.2 Hz); 127.1; 129.7; 136.0; 143.3; HRMS (ESI): m/z calcd. for $[C_{14}H_{20}FNNaO_2S]^+$ 308.1091, found 308.1096; Mp: 95-96 ^oC.

5-Fluoro-5-methyl-3,3-diphenyltetrahydro-2*H*-pyran (5a)

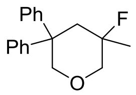

This compound was prepared according to method A. Compound **5a** was isolated as a white solid by chromatography using pentane:Et_2O (15:1) eluent system (50.3 mg, 62%). ^{1}H-NMR (400 MHz, $CDCl_3$) δ 1.22 (d, J = 21.7 Hz, 3H); 2.59 (dd, J = 26.4, 13.5 Hz, 1H); 2.82-2.96 (m, 1H); 3.57 (dd, J = 24.0, 12.0 Hz,

1H); 3.79-3.94 (m, 2H); 4.41 (dd, J = 12.3, 1.9 Hz, 1H); 7.15-7.45 (m, 10H); ^{19}F-NMR (376 MHz, $CDCl_3$) δ -145.57 (br); ^{13}C-NMR (100 MHz, $CDCl_3$) δ 24.1 (d, J_{CF} = 24.2 Hz); 44.4 (d, J_{CF} = 19.8 Hz); 45.7 (d, J_{CF} = 2.2 Hz); 74.23 (d, J_{CF} = 25.7 Hz); 74.32; 90.8 (d, J_{CF} = 174.1 Hz); 125.9; 126.3; 127.0; 127.5; 128.0; 128.4; 145.6; 145.8; HRMS (ESI): m/z calcd. for $[C_{18}H_{19}FNaO]^+$ 293.1312, found 293.1301; Mp: 138-139 ^oC.

5-Cyclohexyl-3-fluoro-3-methyltetrahydro-2*H*-pyran (5b)

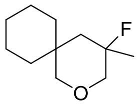

This compound was prepared according to method A. Compound **5b** was isolated as a liquid by chromatography using pentane:Et_2O (15:1) eluent system (36.3 mg, 65%). ^{1}H-NMR (400 MHz, $CDCl_3$) δ 1.12-1.17 (m, 2H); 1.23 (d, J = 20.1 Hz, 3H); 1.34-1.54 (m, 7H); 1.64-1.74 (m, 2H); 1.99-2.11 (m, 1H); 2.99 (d, J = 11.4 Hz, 1H); 3.23 (dd, J = 35.1, 12.5 Hz, 1H); 3.78-3.93 (m, 2H); ^{19}F-NMR (376 MHz, $CDCl_3$) δ -146.74 (m); ^{13}C-NMR (100 MHz, $CDCl_3$) δ 21.5 (d, J_{CF} = 16.9 Hz); 24.1; 24.3; 26.4; 32.8 (d, J_{CF} = 5.2 Hz); 36.8; 43.9 (d, J_{CF} = 20.7 Hz); 74.6; 74.8; 76.0; 91.8 (d, J_{CF} = 172.4 Hz); HRMS (ESI): m/z calcd. for $[C_{11}H_{19}FNaO]^+$ 209.1312, found 209.1321.

6-Cyclohexyl-3-fluoro-3-methyloxepane (5c)

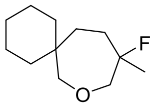

This compound was prepared according to method A. Compound **5c** was isolated as a liquid by chromatography using pentane:Et_2O (15:1) eluent system (36.1 mg, 60%). ^{1}H-NMR (400 MHz, $CDCl_3$) δ 1.22-1.48 (m, 14H); 1.66-1.96 (m, 3H); 3.29 (d, J = 12.3 Hz, 1H); 3.44 (dd, J = 30.0, 13.6 Hz, 1H); 3.62 (d, J = 12.4 Hz, 1H); 3.89 (dd, J = 18.6, 13.5 Hz, 1H); ^{19}F-NMR (376 MHz, $CDCl_3$) δ -142.33 (m); ^{13}C-NMR (100 MHz, $CDCl_3$) δ 21.6 (d, J_{CF} = 1.5 Hz); 24.9 (d, J_{CF} = 25.1 Hz); 26.4; 30.9; 32.9 (d, J_{CF} = 23.5 Hz); 33.6; 35.9; 37.5; 80.5; 80.7; 82.0;

97.7 (d, J_{CF} = 170.2 Hz); HRMS (ESI): *m*/*z* calcd. for $[C_{12}H_{21}FNaO]^+$ 223.1469, found 223.1473.

Dimethyl 3-fluoro-3-methylcyclopentane-1,1-dicarboxylate (7a)

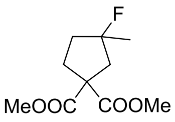

This compound was prepared according to method B. Compound **7a** was isolated as an oil by chromatography using pentane:Et_2O (10:1) eluent system (36.0 mg, 55%). ^{1}H-NMR (400 MHz, $CDCl_3$) δ 1.49 (d, *J* = 21.0 Hz, 3H); 1.66-1.86 (m, 1H); 2.00-2.14 (m, 1H); 2.17-2.27 (m, 1H); 2.30-2.46 (m, 1H); 2.56-2.74 (m, 2H); 3.72 (s, 3H); 3.74 (s, 3H); ^{19}F-NMR (376 MHz, $CDCl_3$) δ -135.03 (m); ^{13}C-NMR (100 MHz, $CDCl_3$) δ 23.8 (d, J_{CF} = 26.4 Hz); 32.7; 38.4 (d, J_{CF} = 23.5 Hz); 46.4 (d, J_{CF} = 24.3 Hz); 52.9 (d, J_{CF} = 11.0 Hz); 59.6; 103.6 (d, J_{CF} = 171.0 Hz); 171.8; 172.8; HRMS (ESI): *m*/*z* calcd. for $[C_{10}H_{15}FNaO_4]^+$ 240.0847, found 240.0870.

Diethyl 3-fluoro-3-methylcyclopentane-1,1-dicarboxylate (7b)

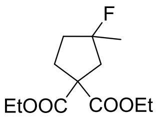

This compound was prepared according to method B. Compound **7b** was isolated as an oil by chromatography using pentane:Et_2O (10:1) eluent system (44.3 mg, 60%). ^{1}H-NMR (400 MHz, $CDCl_3$) δ 1.20-1.27 (m, 6H); 1.48 (d, *J* = 21.0 Hz, 3H); 1.66-1.85 (m, 1H); 2.00-2.13 (m, 1H); 2.14-2.24 (m, 1H); 2.30-2.46 (m, 1H); 2.55-2.71 (m, 2H); 4.13-4.23 (m, 4H); ^{19}F-NMR (376 MHz, $CDCl_3$) δ -134.83 (m); ^{13}C-NMR (100 MHz, $CDCl_3$) δ 14.0; 23.9 (d, J_{CF} = 26.0 Hz); 32.6; 38.4 (d, J_{CF} = 24.2 Hz); 46.3 (d, J_{CF} = 24.6 Hz); 59.7; 61.6 (d, J_{CF} = 14.7 Hz); 103.6 (d, J_{CF} = 171.1 Hz); 171.3; 172.3; HRMS (ESI): *m*/*z* calcd. for $[C_{12}H_{19}FNaO_4]^+$ 269.1160, found 269.1162.

Diethyl 4-fluoro-2,2,4-trimethylcyclopentane-1,1-dicarboxylate (7c)

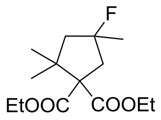

This compound was prepared according to method B. Compound **7c** was isolated as an oil by chromatography using pentane:Et_2O (10:1) eluent system (62.5 mg, 76%). ^{1}H-NMR (400 MHz, $CDCl_3$) δ 1.19 (s, 3H); 1.24-1.35 (m, 9H); 1.54 (d, J = 22.0 Hz, 3H); 2.02-2.29 (m, 2H); 2.47 (dd, J = 18.1, 16.0 Hz, 1H); 2.84-2.99 (m, 1H); 4.11-4.30 (m, 4H); ^{19}F-NMR (376 MHz, $CDCl_3$) δ -118.28 (m); ^{13}C-NMR (100 MHz, $CDCl_3$) δ 14.0; 24.8; 25.6; 27.0; 27.3; 30.9; 45.5; 47.3 (d, J_{CF} = 27.2 Hz); 53.6 (d, J_{CF} = 22.9 Hz); 61.1 (d, J_{CF} = 24.2 Hz); 66.9; 101.6 (d, J_{CF} = 171.5 Hz); 170.1; 171.6; HRMS (ESI): *m*/*z* calcd. for $[C_{14}H_{23}FNaO_4]^+$ 297.1473, found 297.1479.

Diethyl 4-fluoro-2,4-dimethylcyclopentane-1,1-dicarboxylate (7d)

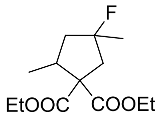

This compound was prepared according to method B. Compound **7d** was isolated as an oil by chromatography using pentane:Et_2O (10:1) eluent system (46.9 mg, 60%). The ^{1}H-NMR and ^{19}F-NMR data of major diastereomer: ^{1}H-NMR (400 MHz, $CDCl_3$) δ 1.02 (d, J = 7.1 Hz, 3H); 1.20-1.29 (m, 6H); 1.49 (d, J = 21.1 Hz, 3H); 1.90-2.30 (m, 2H); 2.40-2.78 (m, 2H); 3.04-3.20 (m, 1H); 4.07-4.28 (m, 4H); ^{19}F-NMR (376 MHz, $CDCl_3$) δ -130.68 (m); the ^{1}H-NMR and ^{19}F-NMR data of minor diastereomer: ^{1}H-NMR (400 MHz, $CDCl_3$) δ 1.10 (d, J = 7.2 Hz, 1.5H); 1.20-1.29 (m, 3H); 1.42 (d, J = 21.7 Hz, 1.5H); 1.90-2.30 (m, 1H); 2.40-2.78 (m, 1H); 2.90-3.04 (m, 0.5H); 4.07-4.28 (m, 2H); ^{19}F-NMR (376 MHz, $CDCl_3$) δ -124.74 (m); ^{13}C-NMR (100 MHz, $CDCl_3$) δ 13.96; 13.98; 14.06; 14.08; 15.9; 16.1; 24.1 (d, J_{CF} = 26.4 Hz); 27.0 (d, J_{CF} = 28.0 Hz); 38.8; 39.5; 46.3 (d, J_{CF} = 22.8 Hz); 46.4 (d, J_{CF} = 22.8 Hz); 46.5 (d, J_{CF} = 17.7 Hz); 47.2 (d, J_{CF} = 24.3 Hz); 61.27; 61.28 (d, J_{CF} = 18.7 Hz); 63.0; 101.4 (d, J_{CF} = 175.5 Hz); 102.3 (d, J_{CF} = 170.0 Hz); 170.0; 171.3; 171.6; 171.8; HRMS (ESI): *m*/*z* calcd. for $[C_{13}H_{21}FNaO_4]^+$ 283.1316, found 283.1325.

References

[1] a) G. C. Geary, E. G. Hope, K. Singh, A. M. Stuart, *Chem. Commun.* **2013**, *49*, 9263; b) V. Matoušek, E. Pietrasiak, R. Schwenk, A. Togni, *J. Org. Chem.* **2013**, *78*, 6763; c) P. Eisenberger, I. Kieltsch, R. Koller, K. Stanek, A. Togni, *Org. Synth.* **2011**, *88*, 168.

[2] a) W. Q. Kong, P. Feige, T. D. Haro, C. Nevado, *Angew. Chem. Int. Ed.* **2013**, *52*, 2469; b) A. P. Dobbs, S. J. J. Guesné, R. J. Parker, J. Skidmore, R. A. Stephenson, M. B. Hursthouse, *Org. Biomol. Chem.* **2010**, *8*, 1064; c) Y. Jeong, D-Y. Kim, Y. Choi, J-S. Ryu, *Org. Biomol. Chem.* **2011**, *9*, 374; d) J. Kempson, W. J. Pitts, J. Barbosa, J. Guo, O. Omotoso, A. Watson, K. Stebbins, G. C. Starling, J. H. Dodd, J. C. Barrish, R. Felix, K. Fischer, *Bioorg. Med. Chem. Lett.* **2005**, *15*, 1829.

[3] T. Wu, G. Y. Yin, G. S. Liu, *J. Am. Chem. Soc.* **2009**, *131*, 16354.

[4] J. Cui, Q. Jia, R-Z. Feng, S-S. Liu, T. He, C. Zhang, *Org. Lett.* **2014**, *16*, 1442.

NMR spectra of products 3a-3n, 5a-5c and 7a-7d

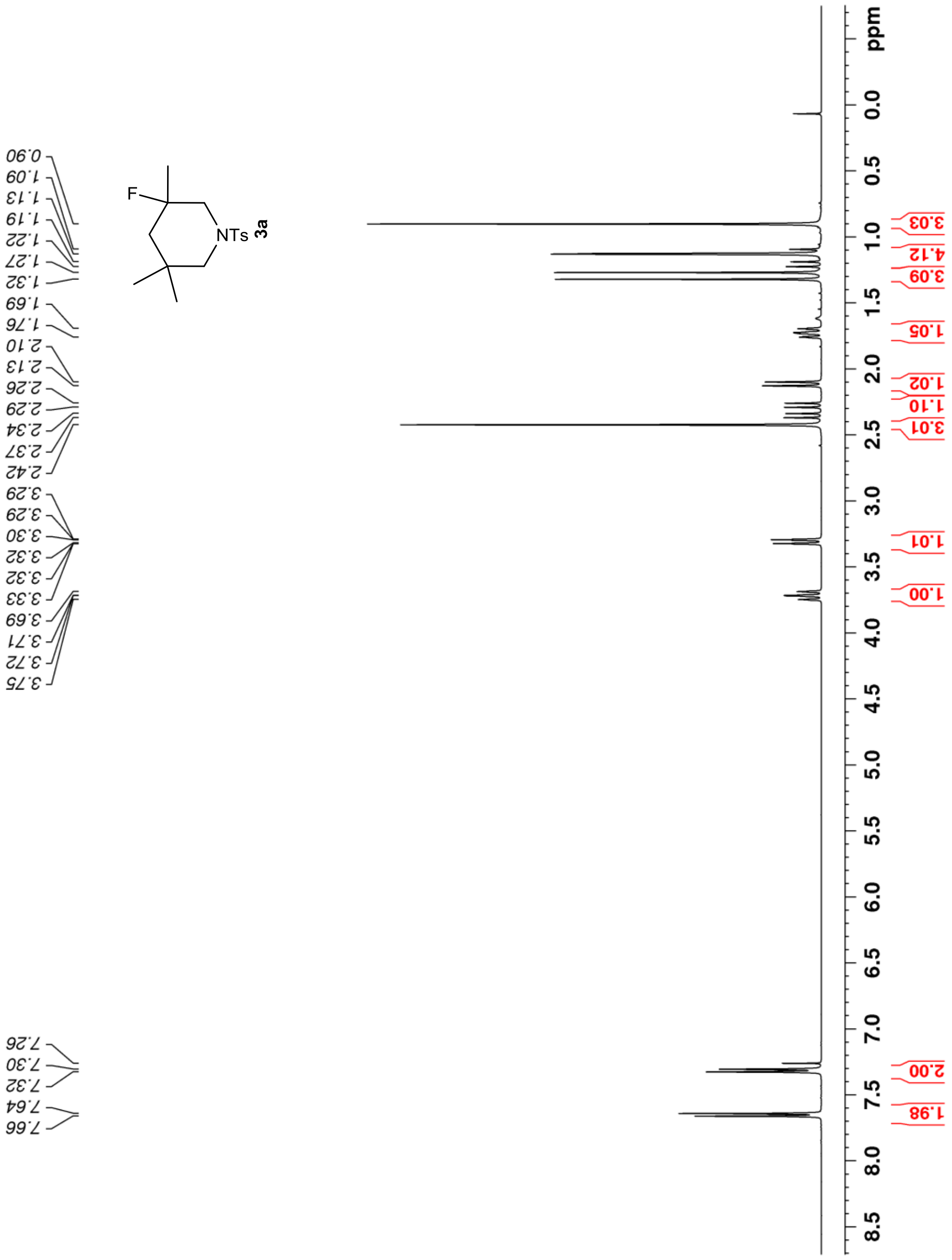

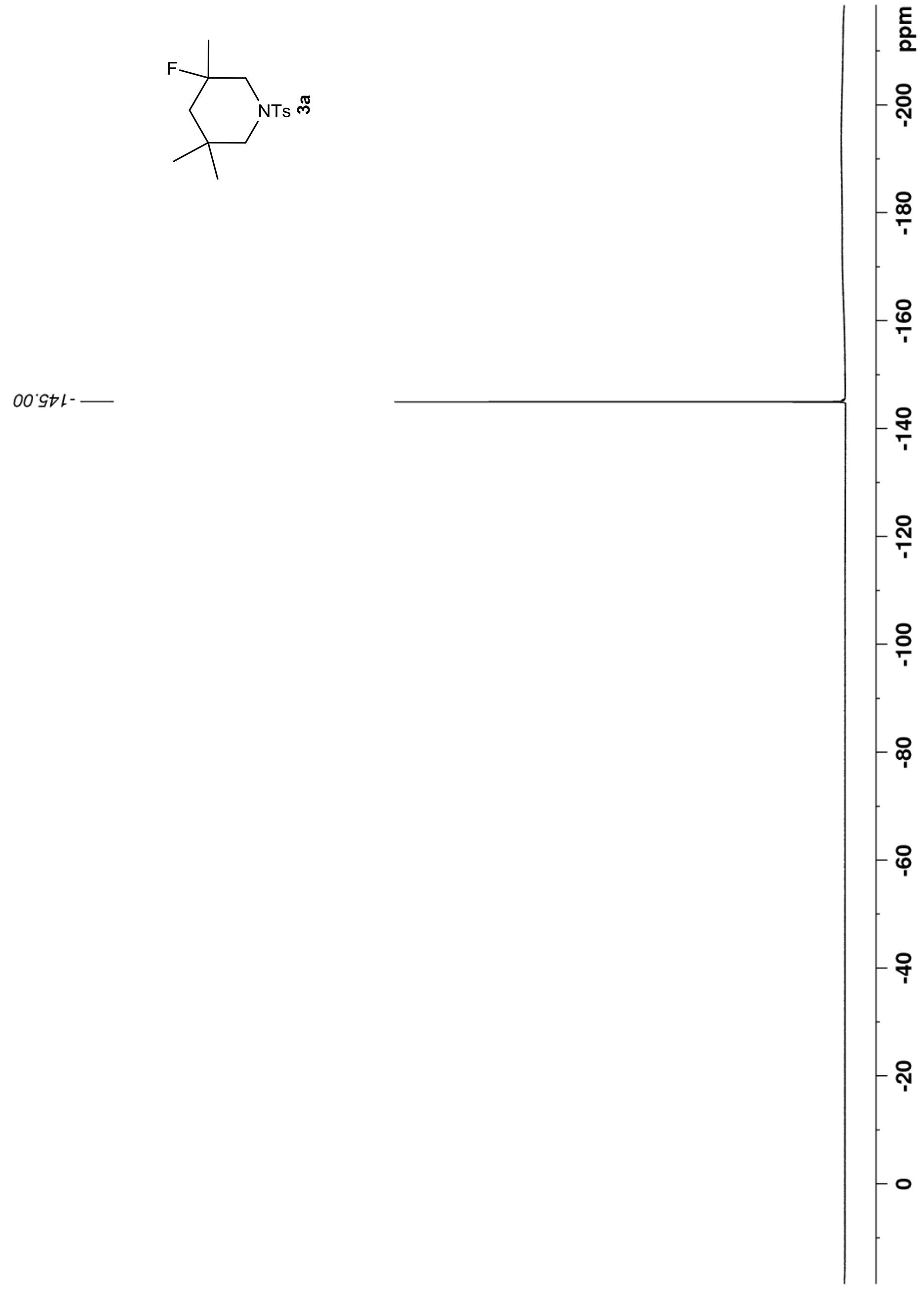
F
NTs
3a
-145.00
ppm
-200
-180
-160
-140
-120
-100
-80
-60
-40
-20
0

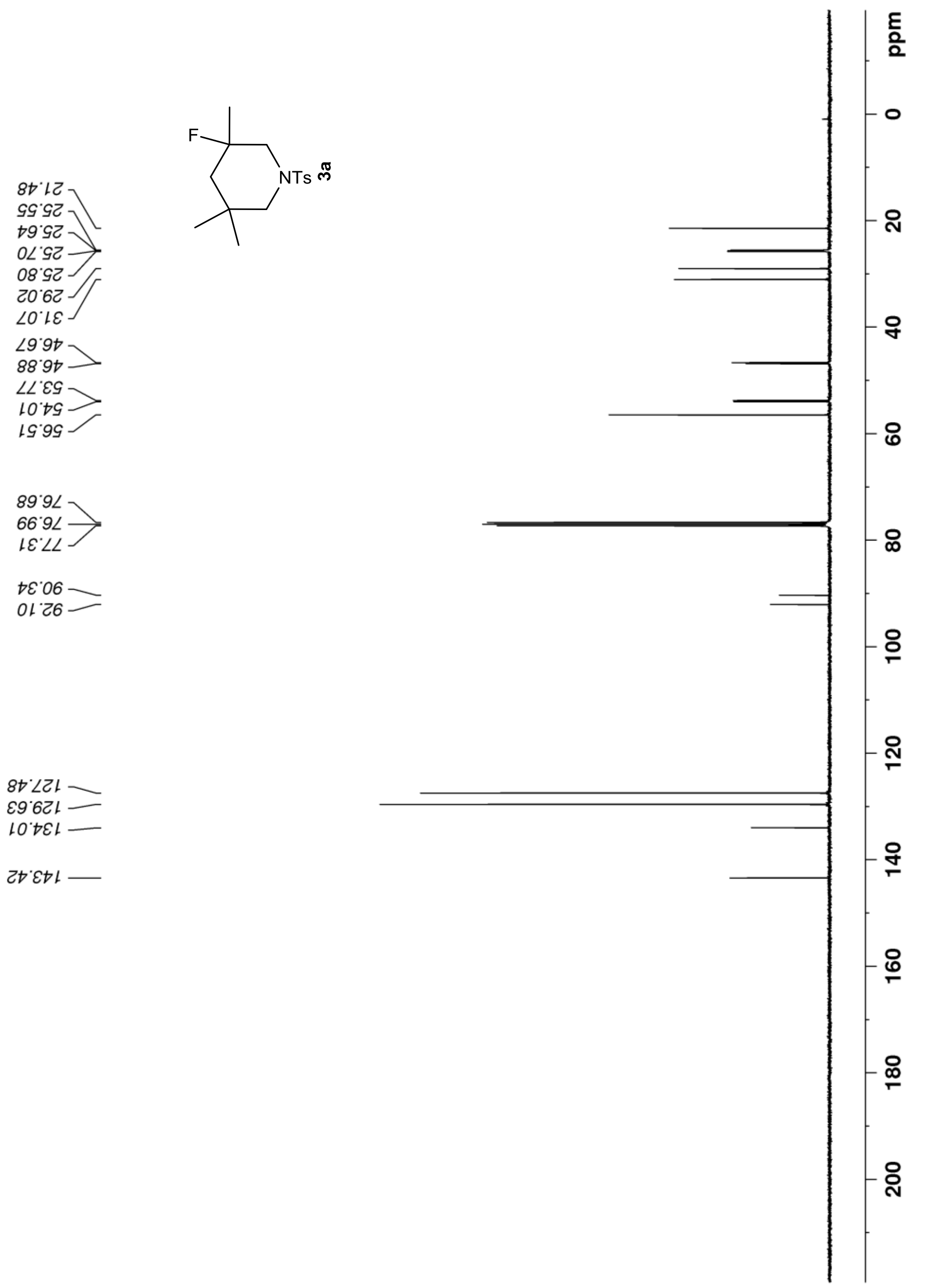
21.48
25.55
25.64
25.70
25.80
29.02
31.07
46.67
46.88
53.77
54.01
56.51
76.68
76.99
77.31
90.34
92.10
127.48
129.63
134.01
143.42
F
NTs
3a
ppm
0
20
40
60
80
100
120
140
160
180
200

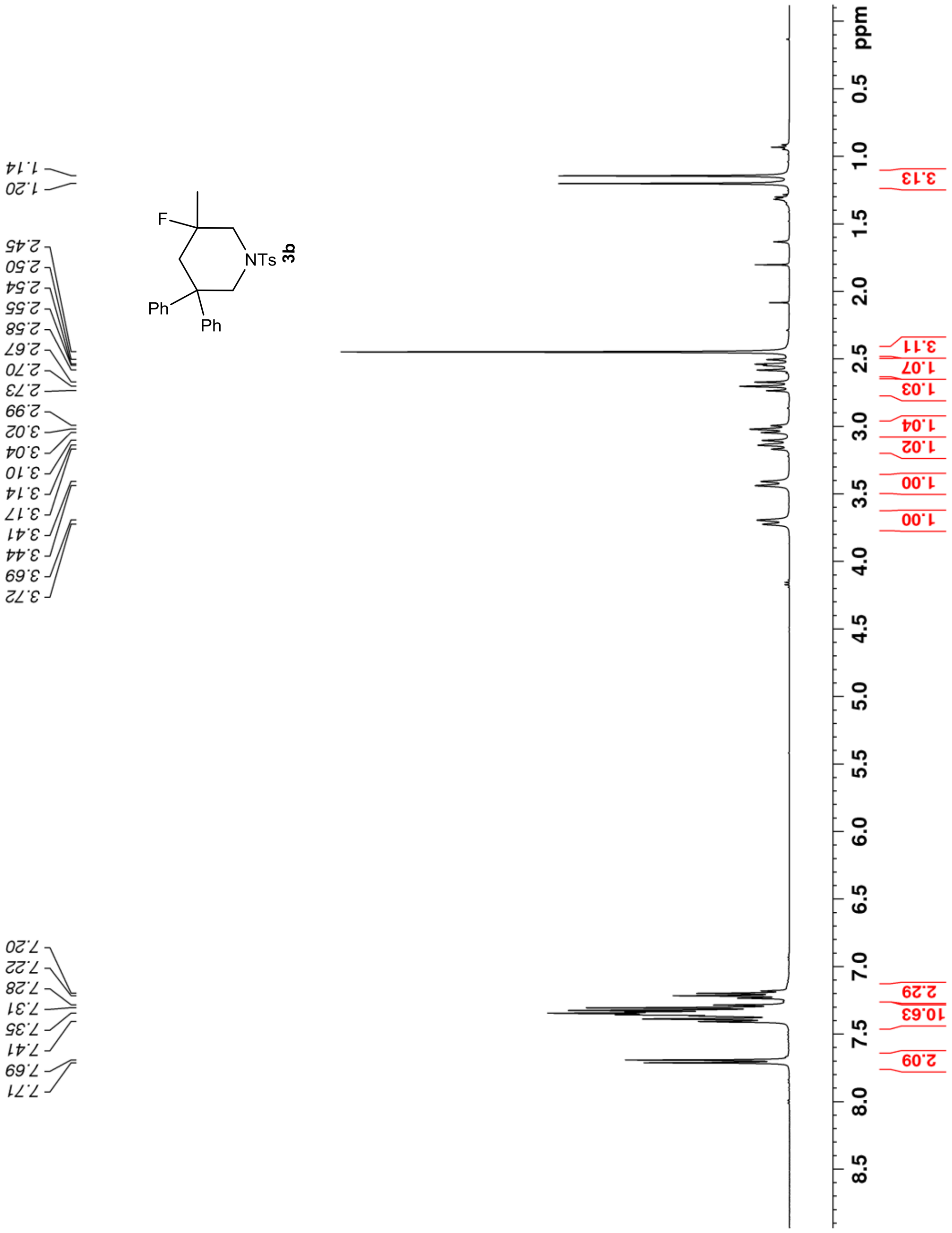

F
NTs
3b
Ph
Ph
1.14
1.20
2.45
2.50
2.54
2.55
2.58
2.67
2.70
2.73
2.99
3.02
3.04
3.10
3.14
3.17
3.41
3.44
3.69
3.72
7.20
7.22
7.28
7.31
7.35
7.41
7.69
7.71
3.13
3.11
1.07
1.03
1.04
1.02
1.00
1.00
2.29
10.63
2.09
ppm

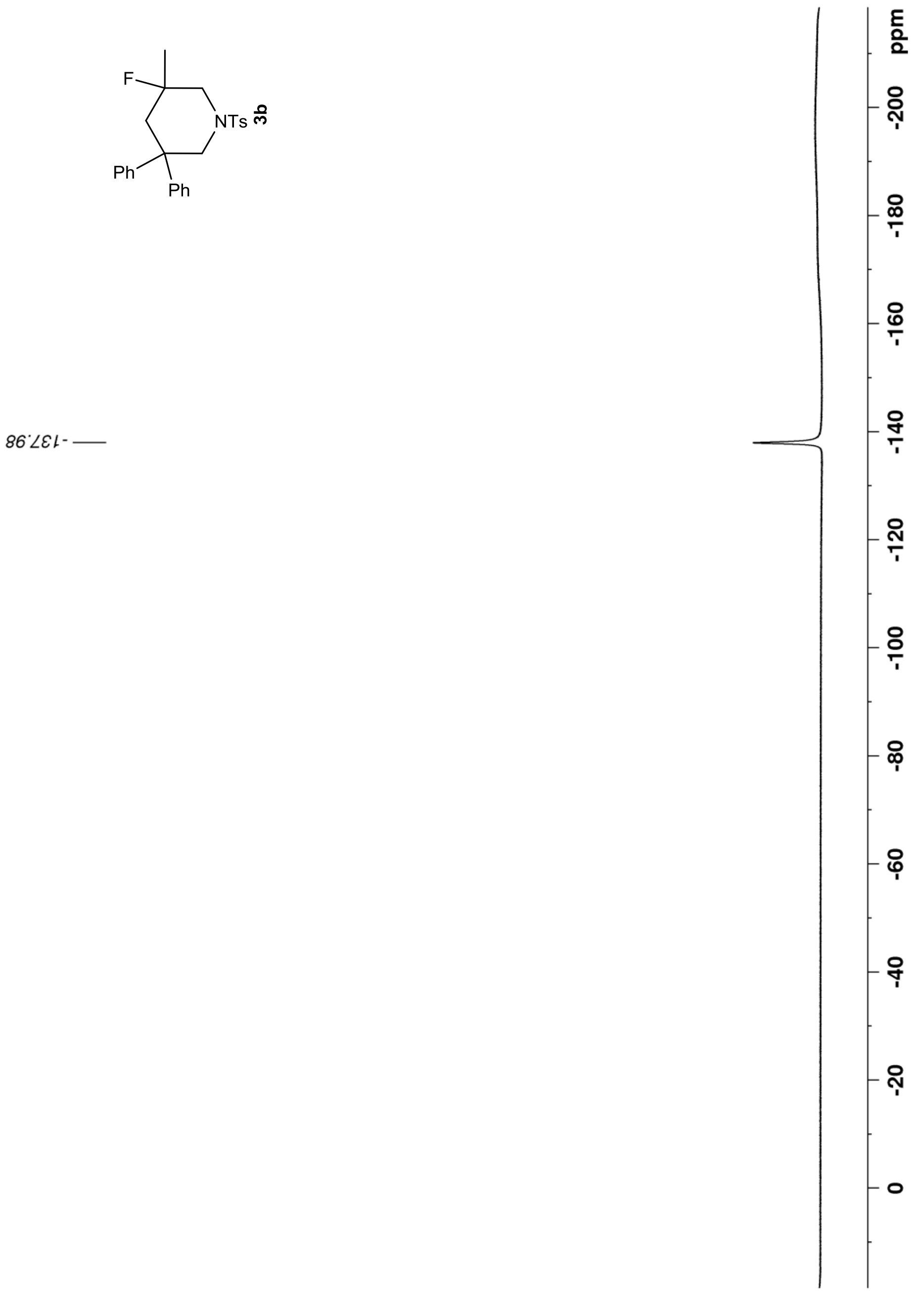
F
NTs
3b
Ph
Ph
-137.98
ppm
-200
-180
-160
-140
-120
-100
-80
-60
-40
-20
0

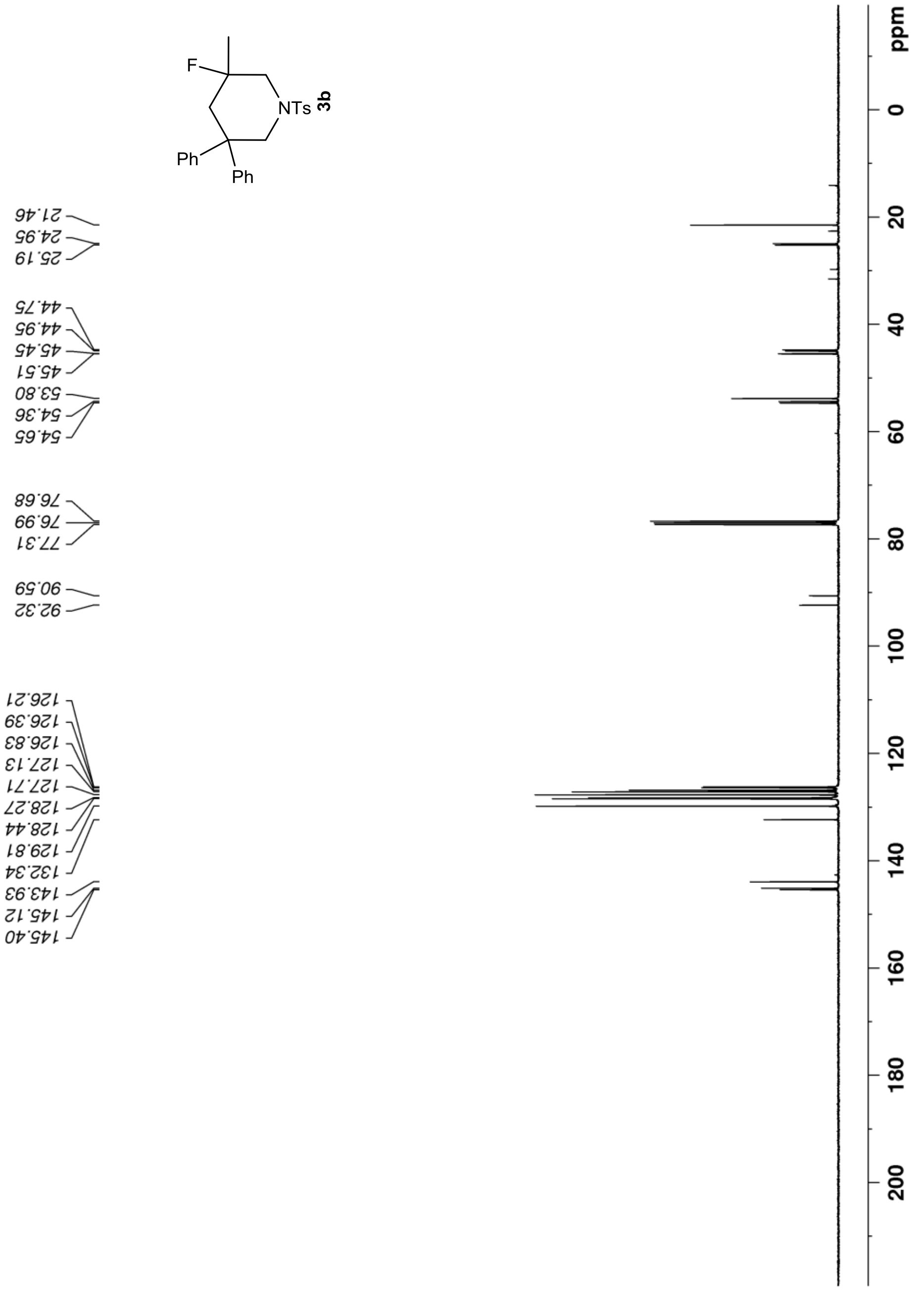
F
NTs
3b
Ph
Ph
21.46
24.95
25.19
44.75
44.95
45.45
45.51
53.80
54.36
54.65
76.68
76.99
77.31
90.59
92.32
126.21
126.39
126.83
127.13
127.71
128.27
128.44
129.81
132.34
143.93
145.12
145.40
ppm
0
20
40
60
80
100
120
140
160
180
200

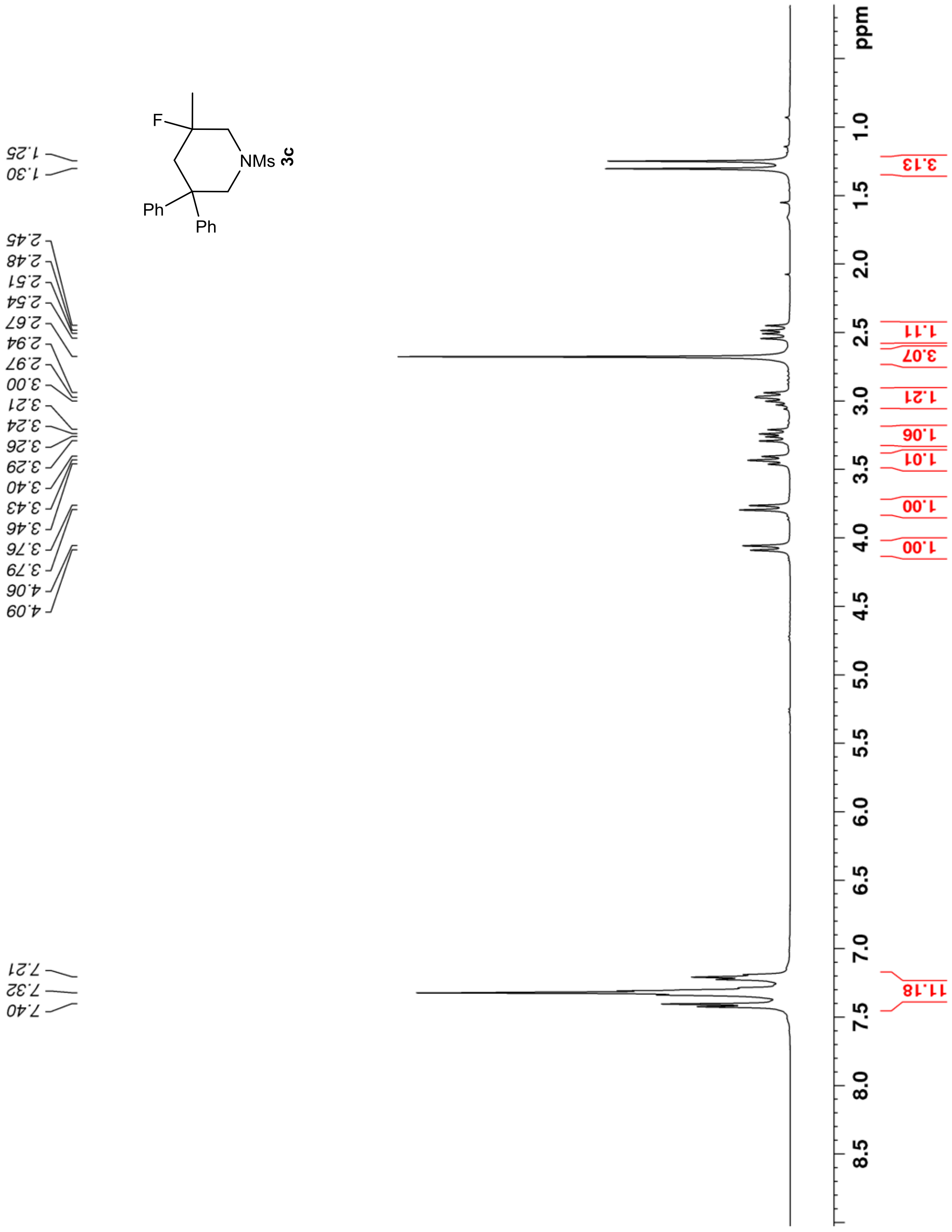
1.25
1.30
2.45
2.48
2.51
2.54
2.67
2.94
2.97
3.00
3.21
3.24
3.26
3.29
3.40
3.43
3.46
3.76
3.79
4.06
4.09
7.21
7.32
7.40
F
NMs
3c
Ph
Ph
3.13
1.11
3.07
1.21
1.06
1.01
1.00
1.00
11.18
ppm
1.0
1.5
2.0
2.5
3.0
3.5
4.0
4.5
5.0
5.5
6.0
6.5
7.0
7.5
8.0
8.5

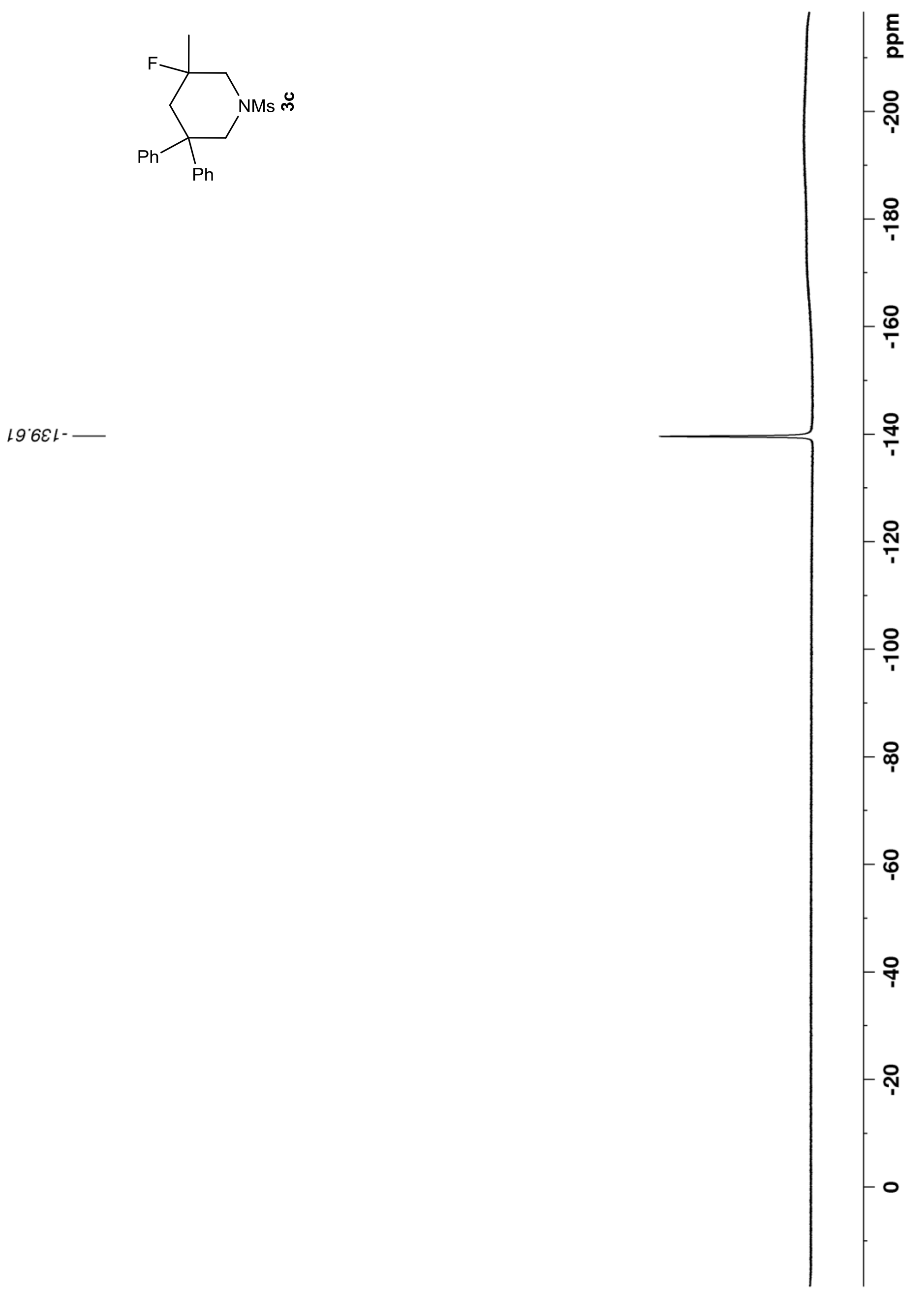

F
NMs
3c
Ph
Ph
-139.61
ppm
-200
-180
-160
-140
-120
-100
-80
-60
-40
-20
0

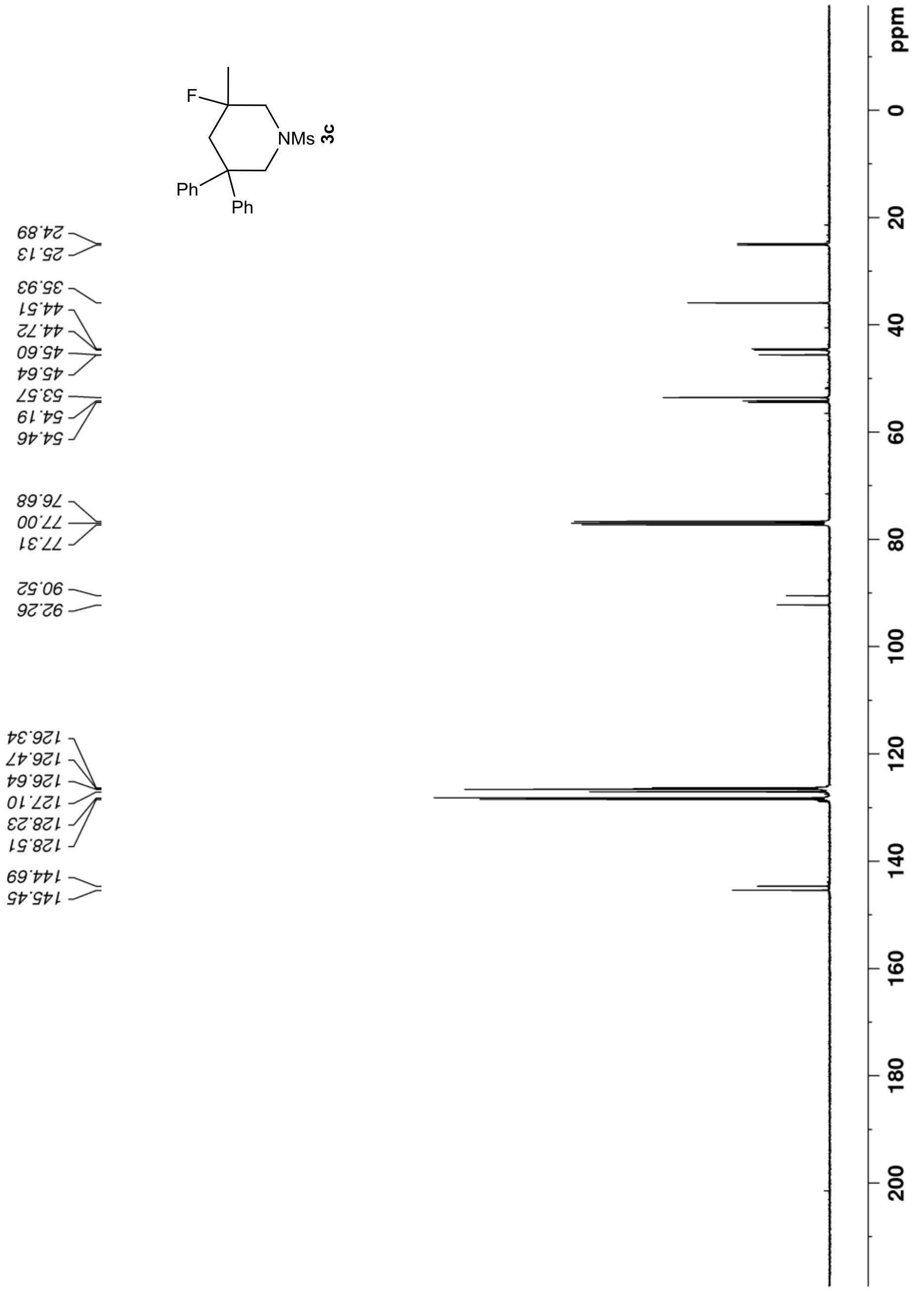
F
NMs
Ph
Ph
3c
24.89
25.13
35.93
44.51
44.72
45.60
45.64
53.57
54.19
54.46
76.68
77.00
77.31
90.52
92.26
126.34
126.47
126.64
127.10
128.23
128.51
144.69
145.45
ppm
0
20
40
60
80
100
120
140
160
180
200

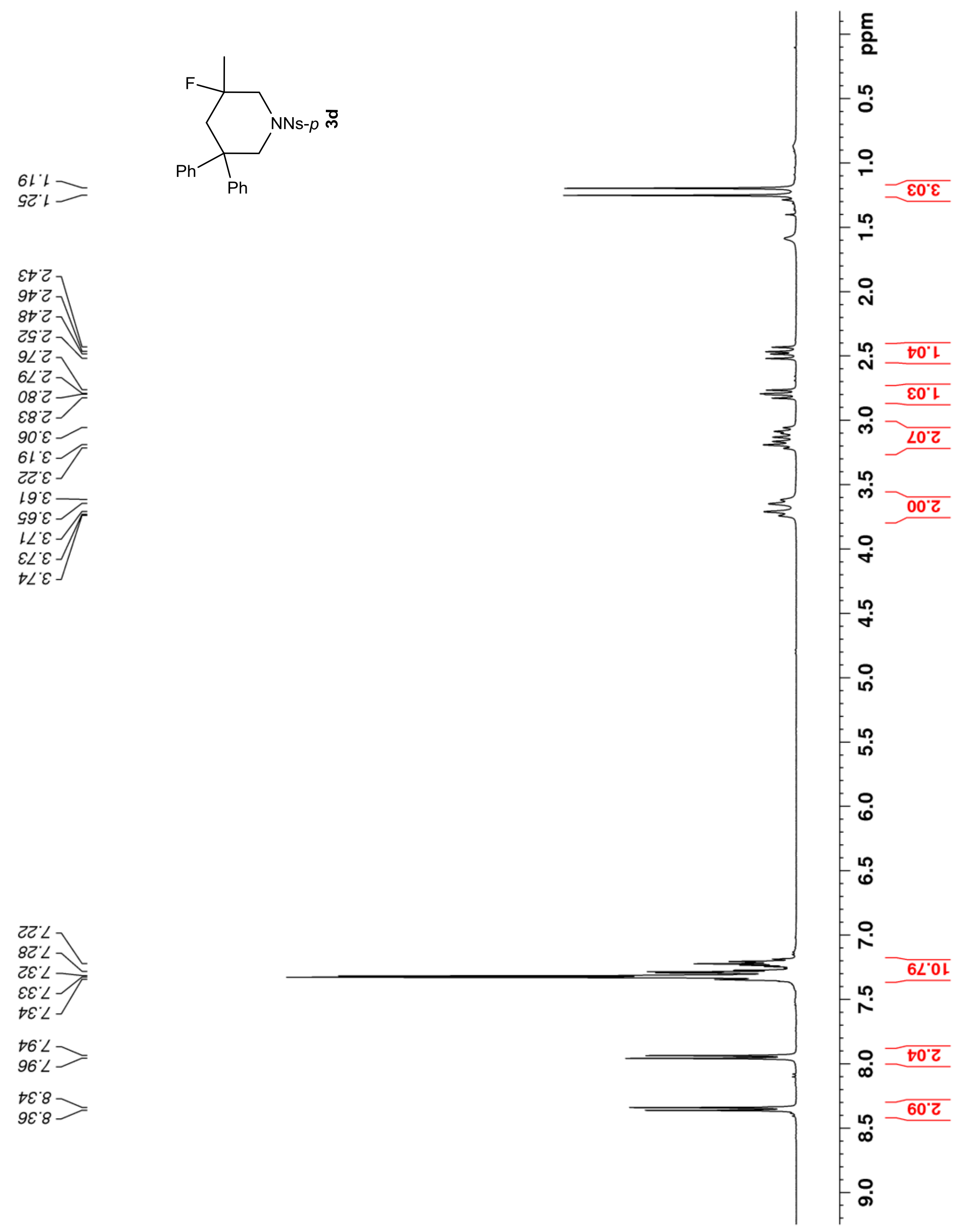
F
NNs-p
3d
Ph
Ph
1.19
1.25
2.43
2.46
2.48
2.52
2.76
2.79
2.80
2.83
3.06
3.19
3.22
3.61
3.65
3.71
3.73
3.74
7.22
7.28
7.32
7.33
7.34
7.94
7.96
8.34
8.36
3.03
1.04
1.03
2.07
2.00
10.79
2.04
2.09
ppm

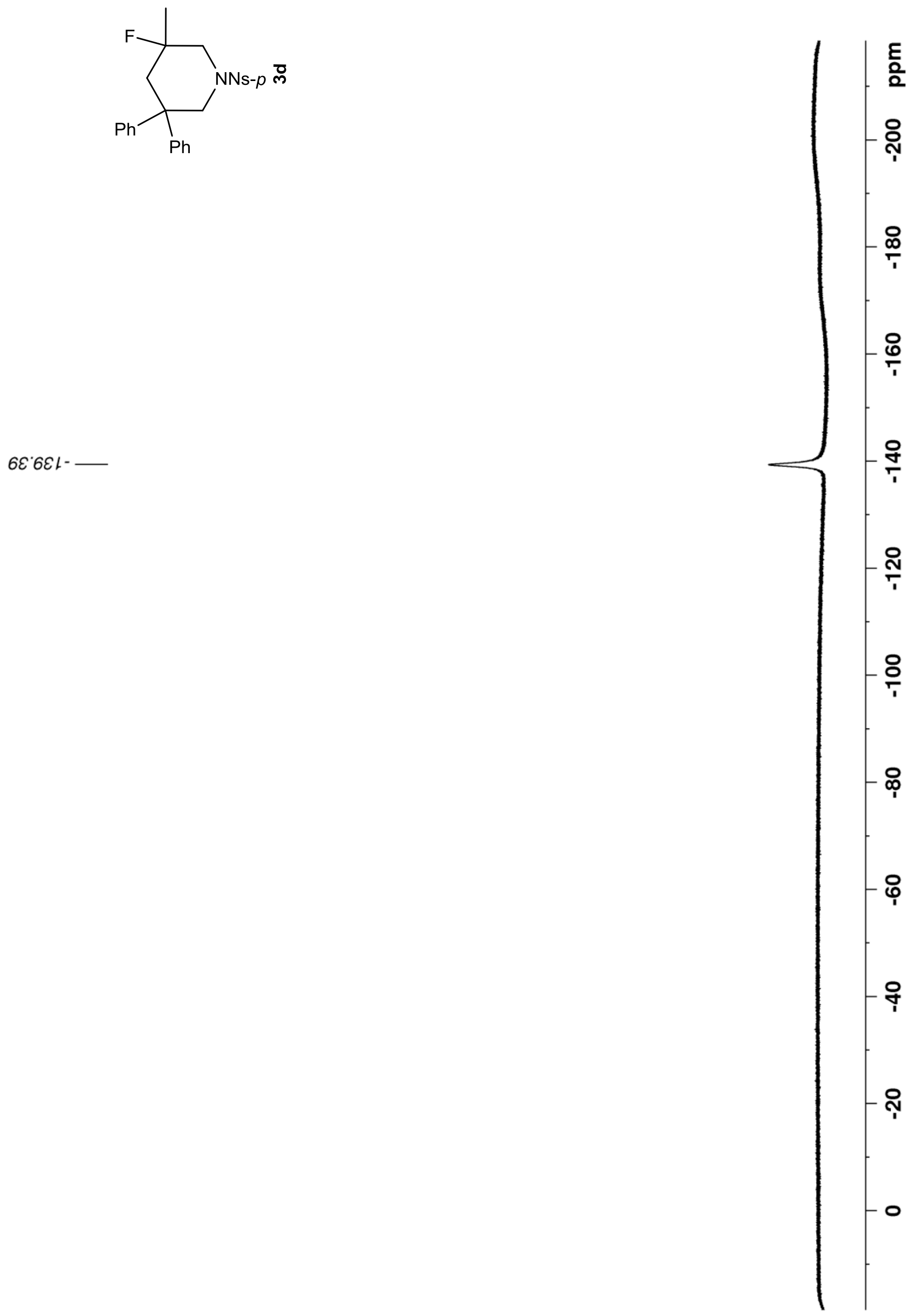
F
NNs-p
3d
Ph
Ph
-139.39
ppm
-200
-180
-160
-140
-120
-100
-80
-60
-40
-20
0

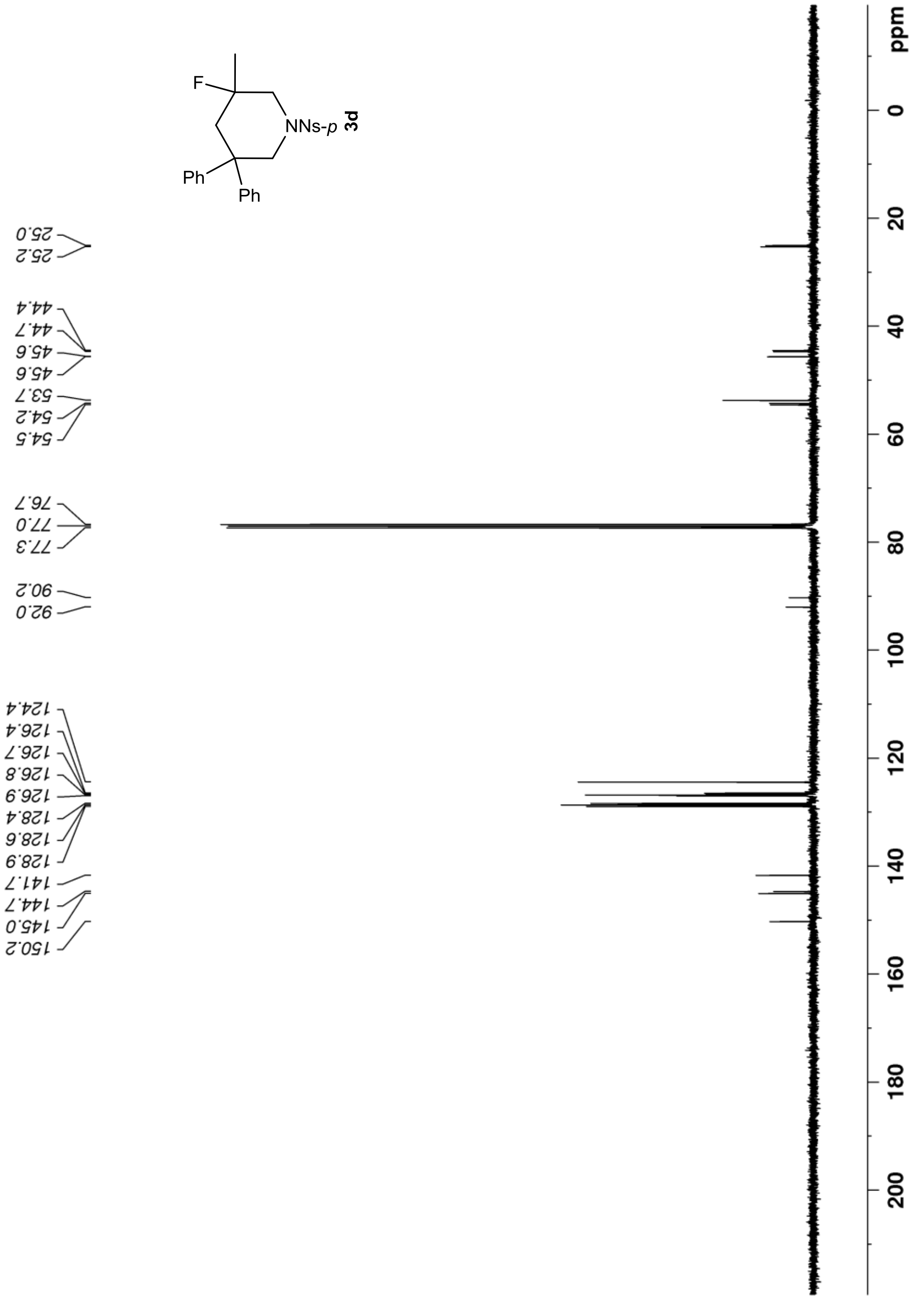

F
NNs-*p*
3d
Ph
Ph
25.0
25.2
44.4
44.7
45.6
45.6
53.7
54.2
54.5
76.7
77.0
77.3
90.2
92.0
124.4
126.4
126.7
126.8
126.9
128.4
128.6
128.9
141.7
144.7
145.0
150.2
ppm
0
20
40
60
80
100
120
140
160
180
200

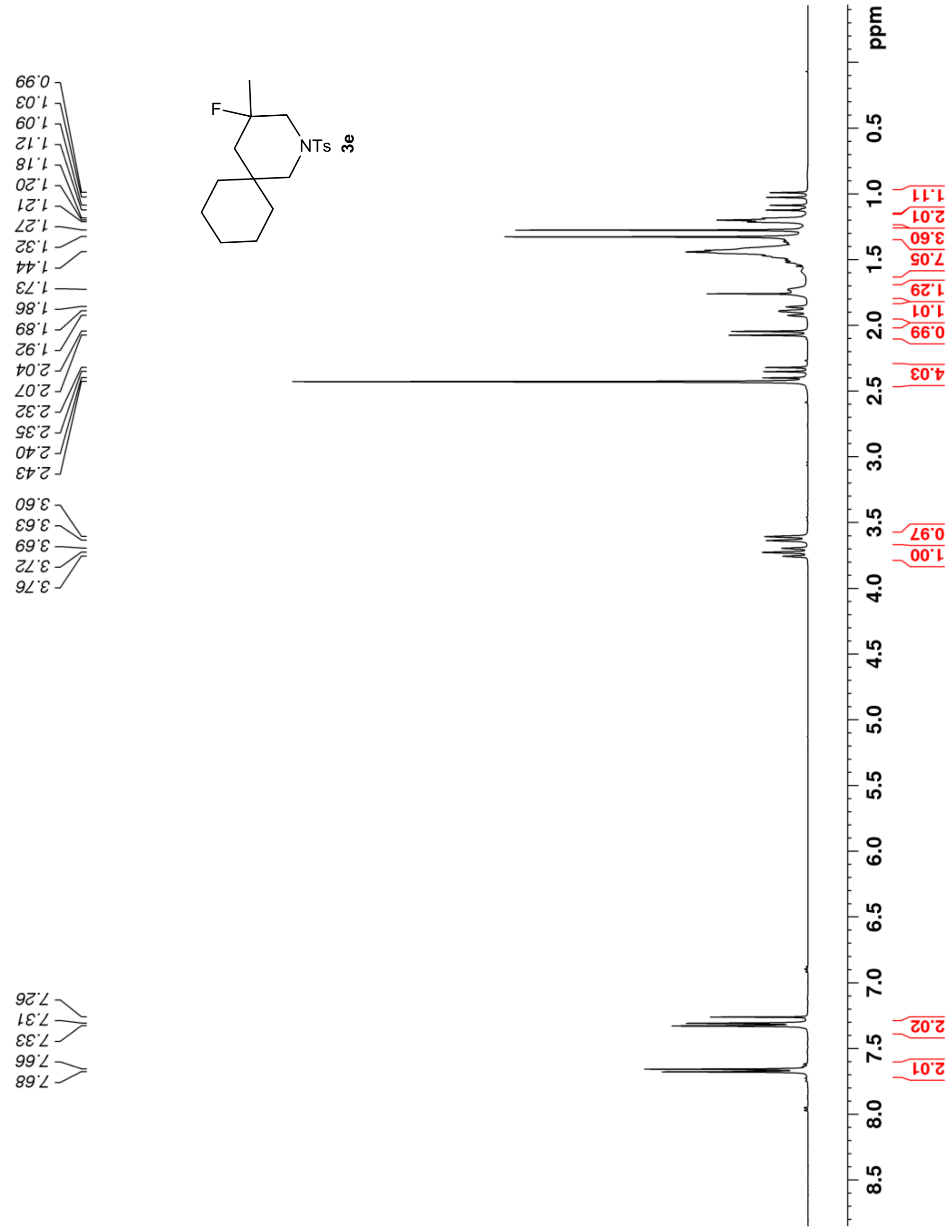
F
NTs
3e
0.99
1.03
1.09
1.12
1.18
1.20
1.21
1.27
1.32
1.44
1.73
1.86
1.89
1.92
2.04
2.07
2.32
2.35
2.40
2.43
3.60
3.63
3.69
3.72
3.76
7.26
7.31
7.33
7.66
7.68
1.11
2.01
3.60
7.05
1.29
1.01
0.99
4.03
0.97
1.00
2.02
2.01
ppm

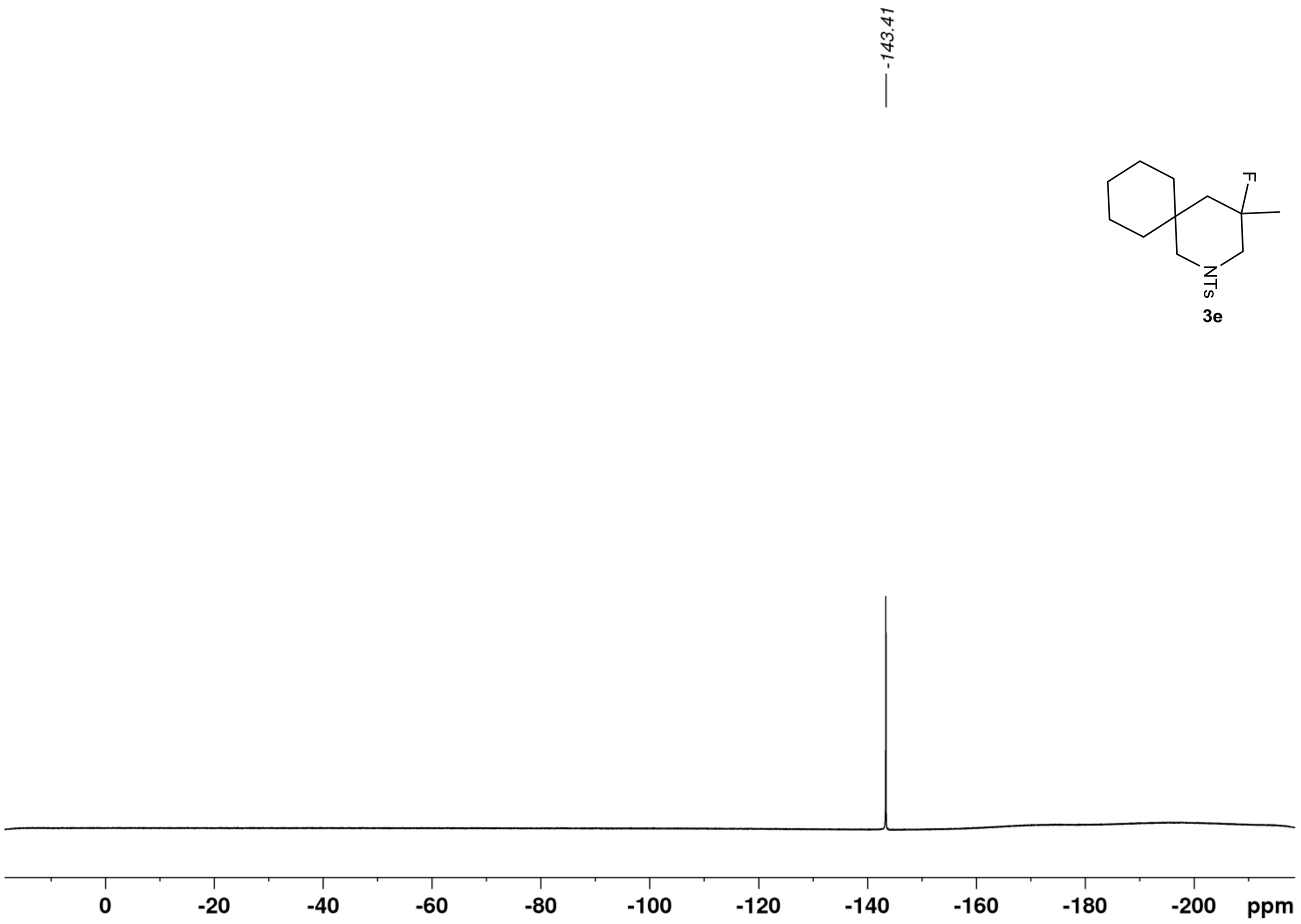

-143.41
F
NTs
3e
0
-20
-40
-60
-80
-100
-120
-140
-160
-180
-200
ppm

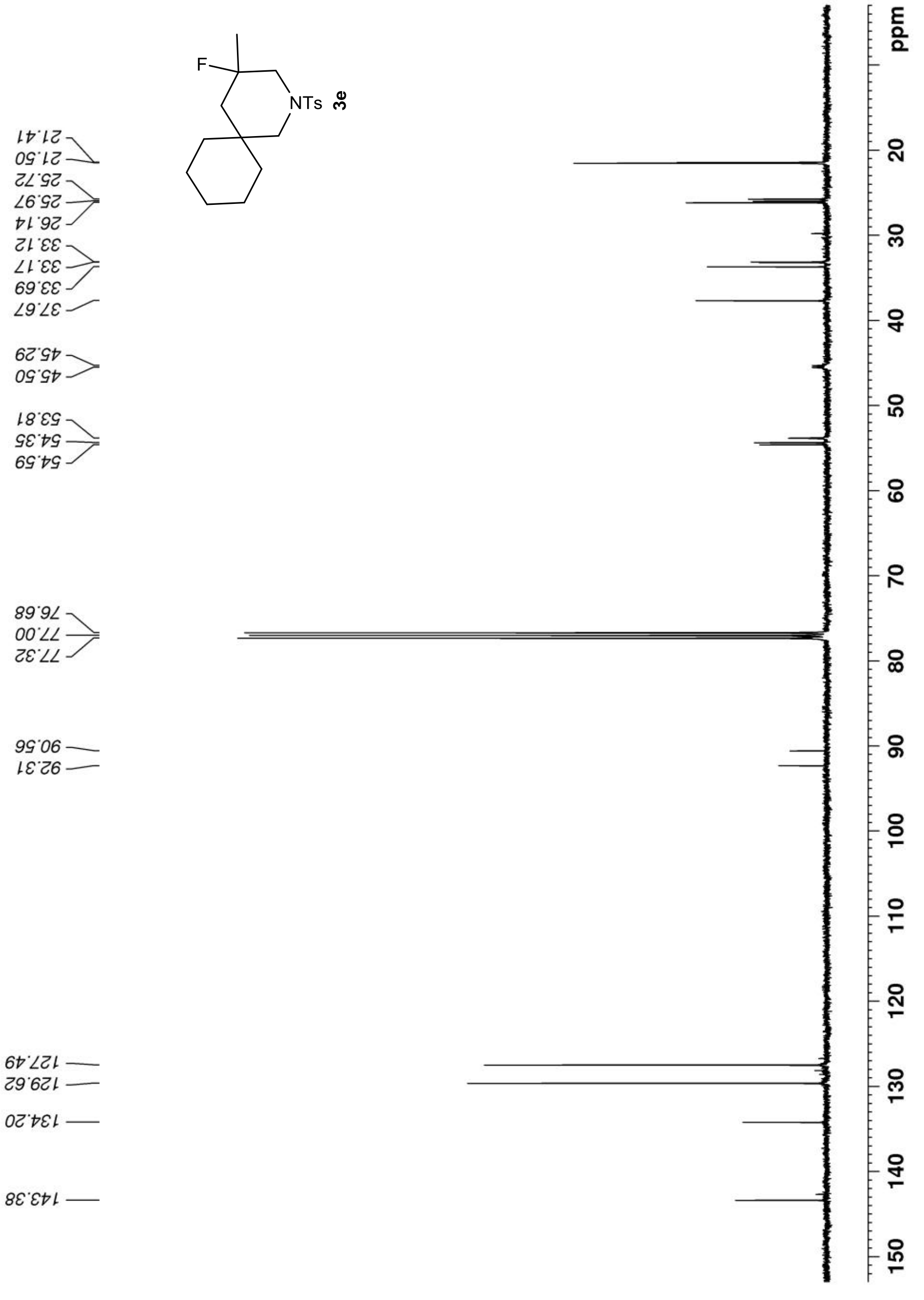
F
NTs
3e
21.41
21.50
25.72
25.97
26.14
33.12
33.17
33.69
37.67
45.29
45.50
53.81
54.35
54.59
76.68
77.00
77.32
90.56
92.31
127.49
129.62
134.20
143.38
ppm
20
30
40
50
60
70
80
90
100
110
120
130
140
150

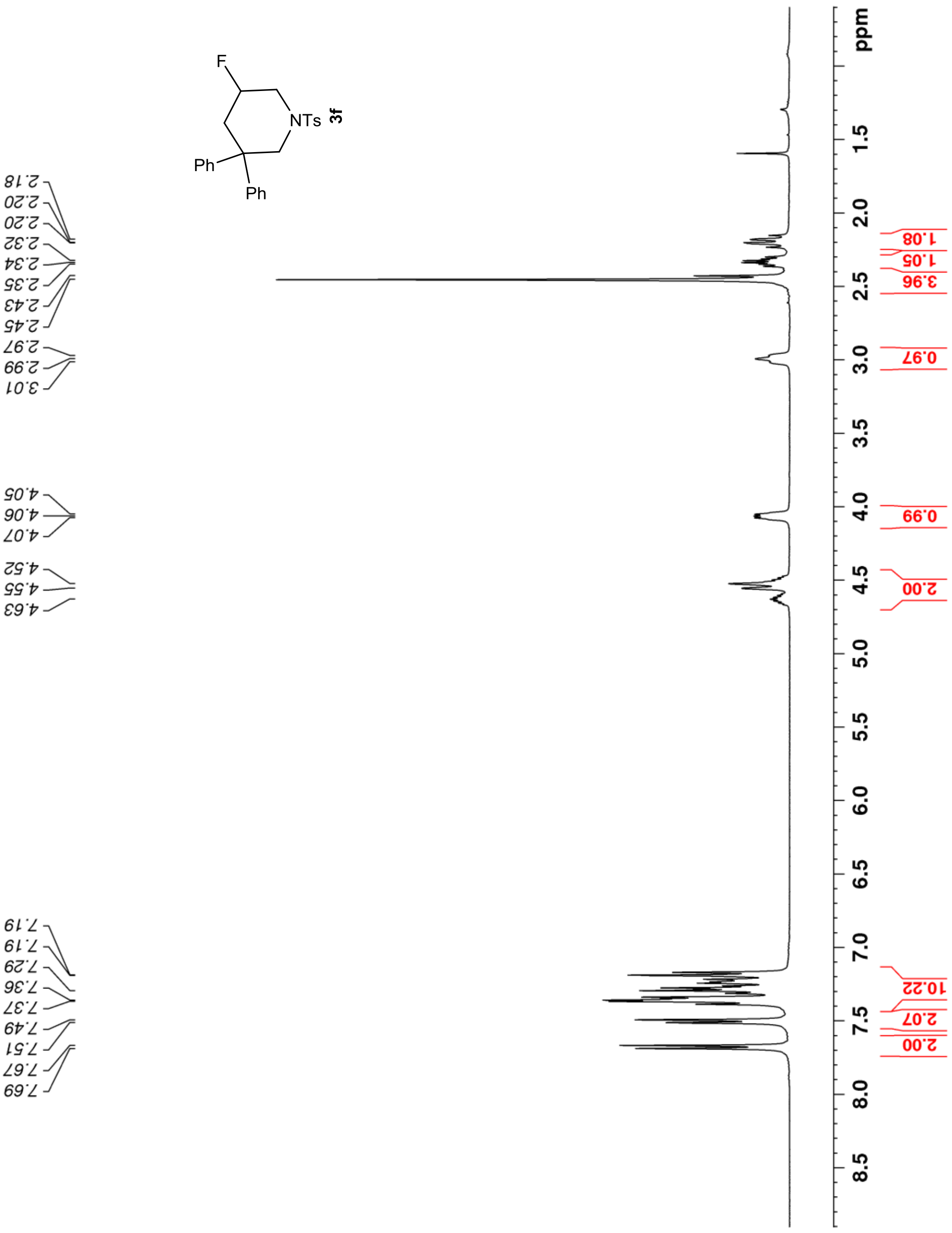
F
NTs
3f
Ph
Ph
2.18
2.20
2.20
2.32
2.34
2.35
2.43
2.45
2.97
2.99
3.01
4.05
4.06
4.07
4.52
4.55
4.63
7.19
7.19
7.29
7.36
7.37
7.49
7.51
7.67
7.69
1.08
1.05
3.96
0.97
0.99
2.00
10.22
2.07
2.00
ppm

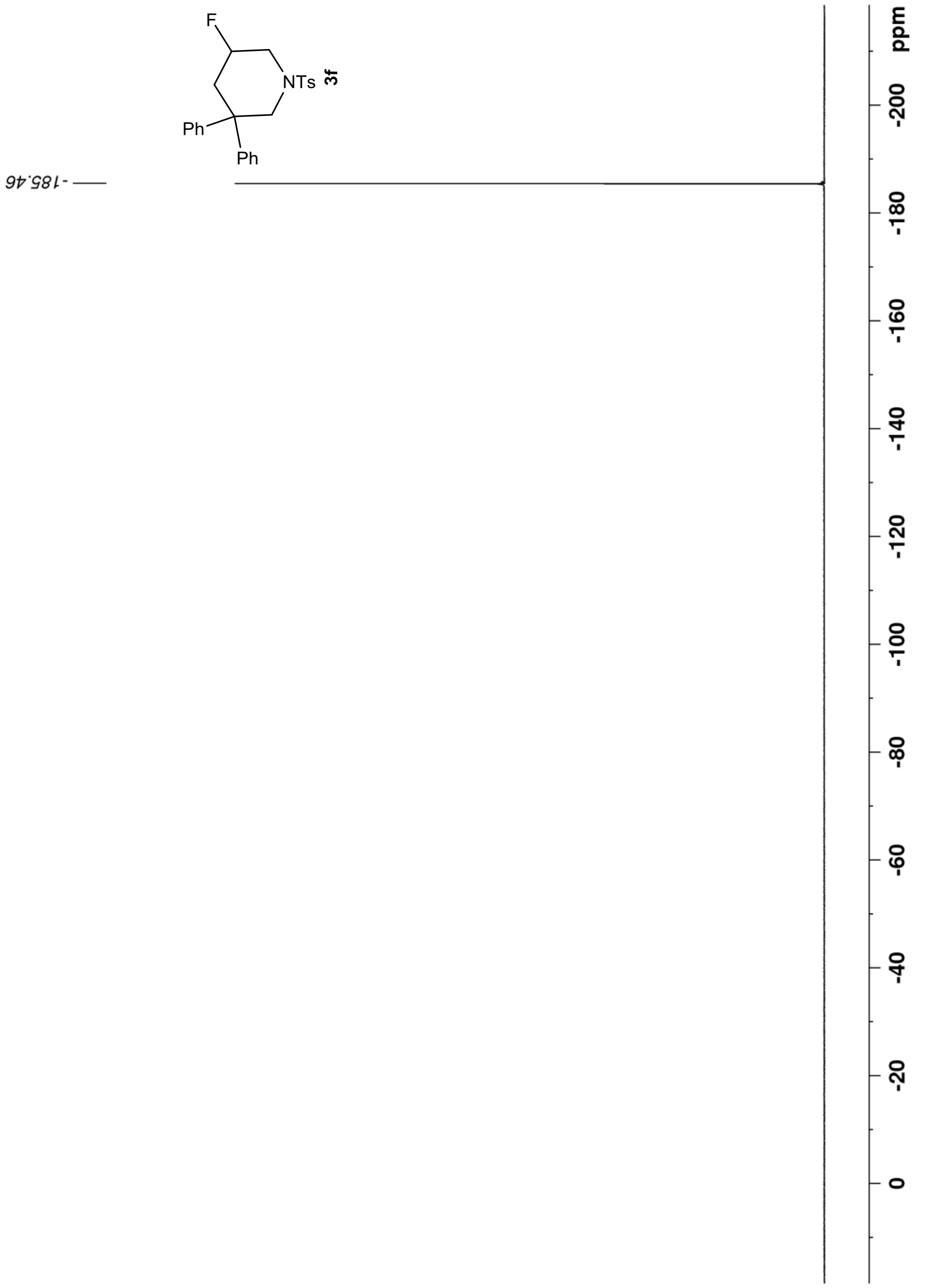
F
NTs
3f
Ph
Ph
-185.46
ppm
-200
-180
-160
-140
-120
-100
-80
-60
-40
-20
0

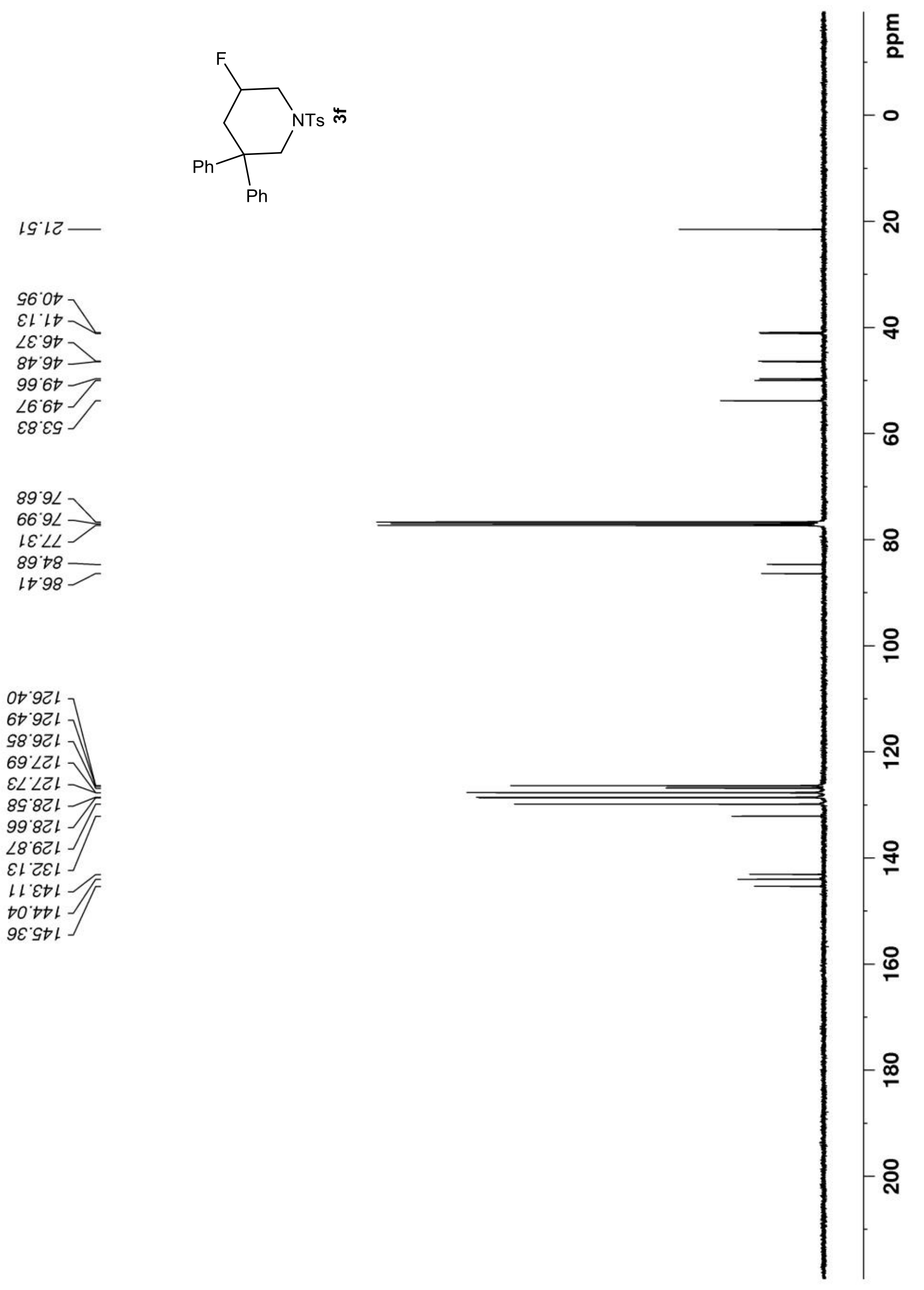
F
NTs
3f
Ph
Ph
21.51
40.95
41.13
46.37
46.48
49.66
49.97
53.83
76.68
76.99
77.31
84.68
86.41
126.40
126.49
126.85
127.69
127.73
128.58
128.66
129.87
132.13
143.11
144.04
145.36
ppm
0
20
40
60
80
100
120
140
160
180
200

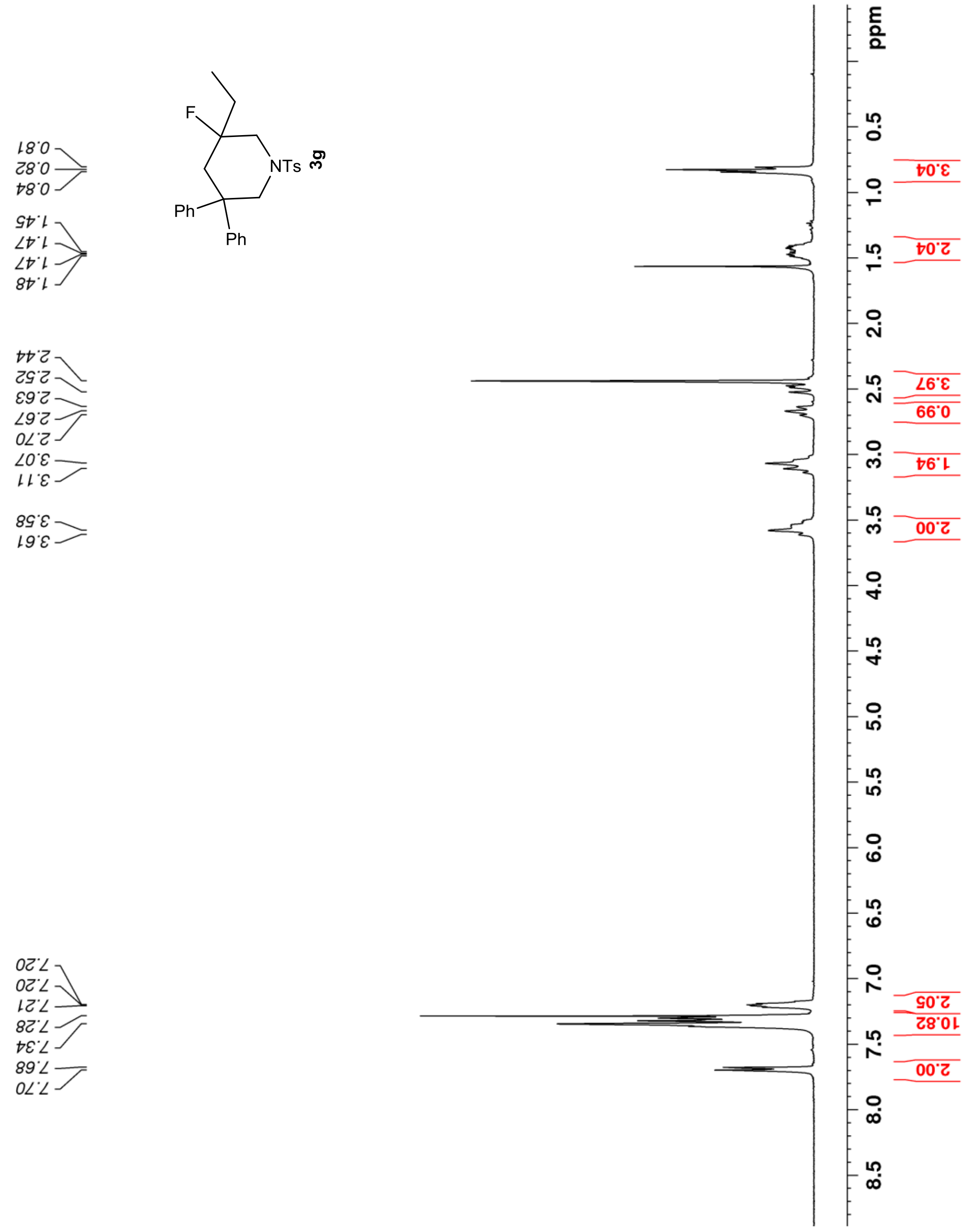
F
NTs
Ph
Ph
3g
ppm

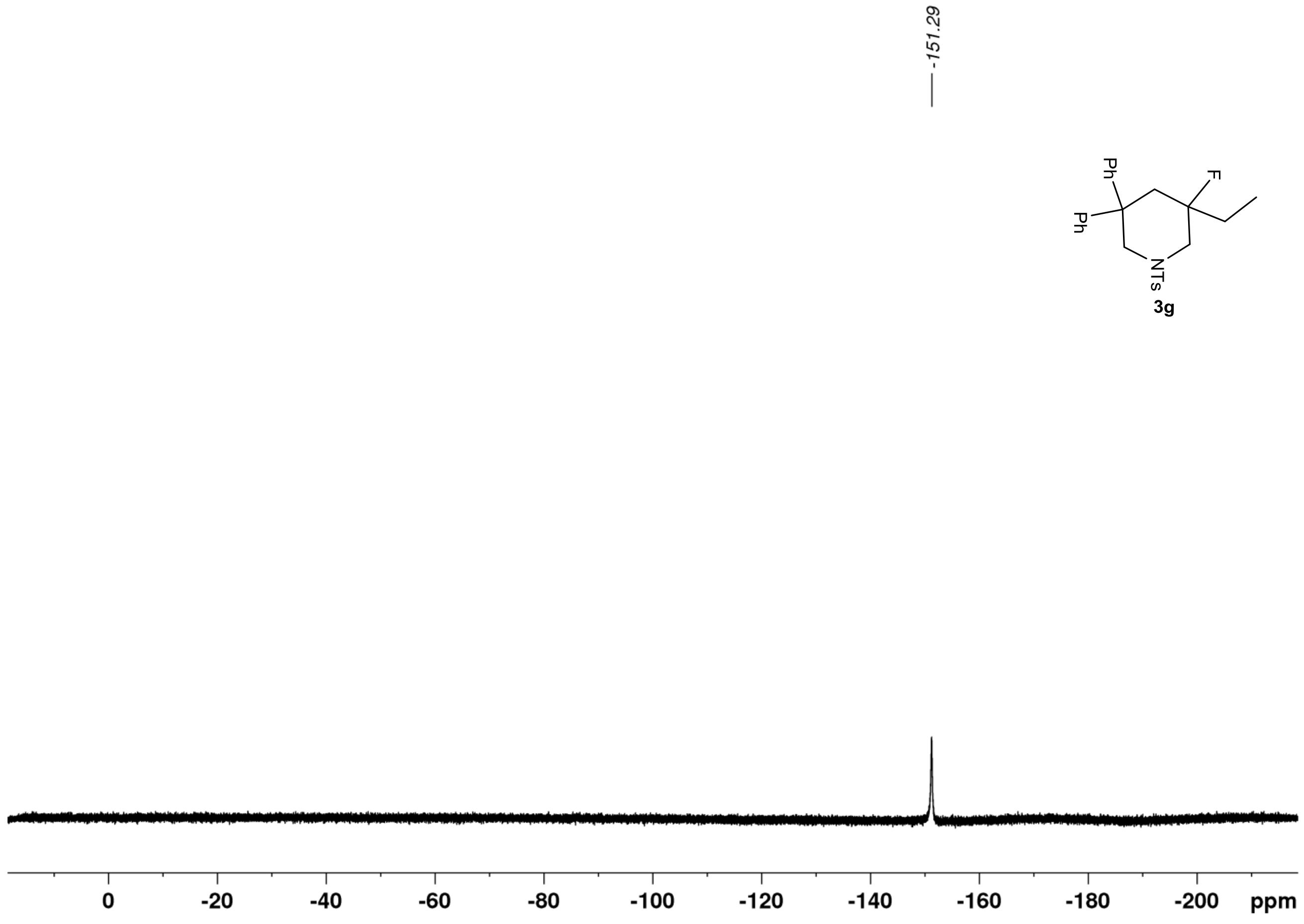
-151.29
F
Ph
Ph
NTs
3g
0
-20
-40
-60
-80
-100
-120
-140
-160
-180
-200
ppm

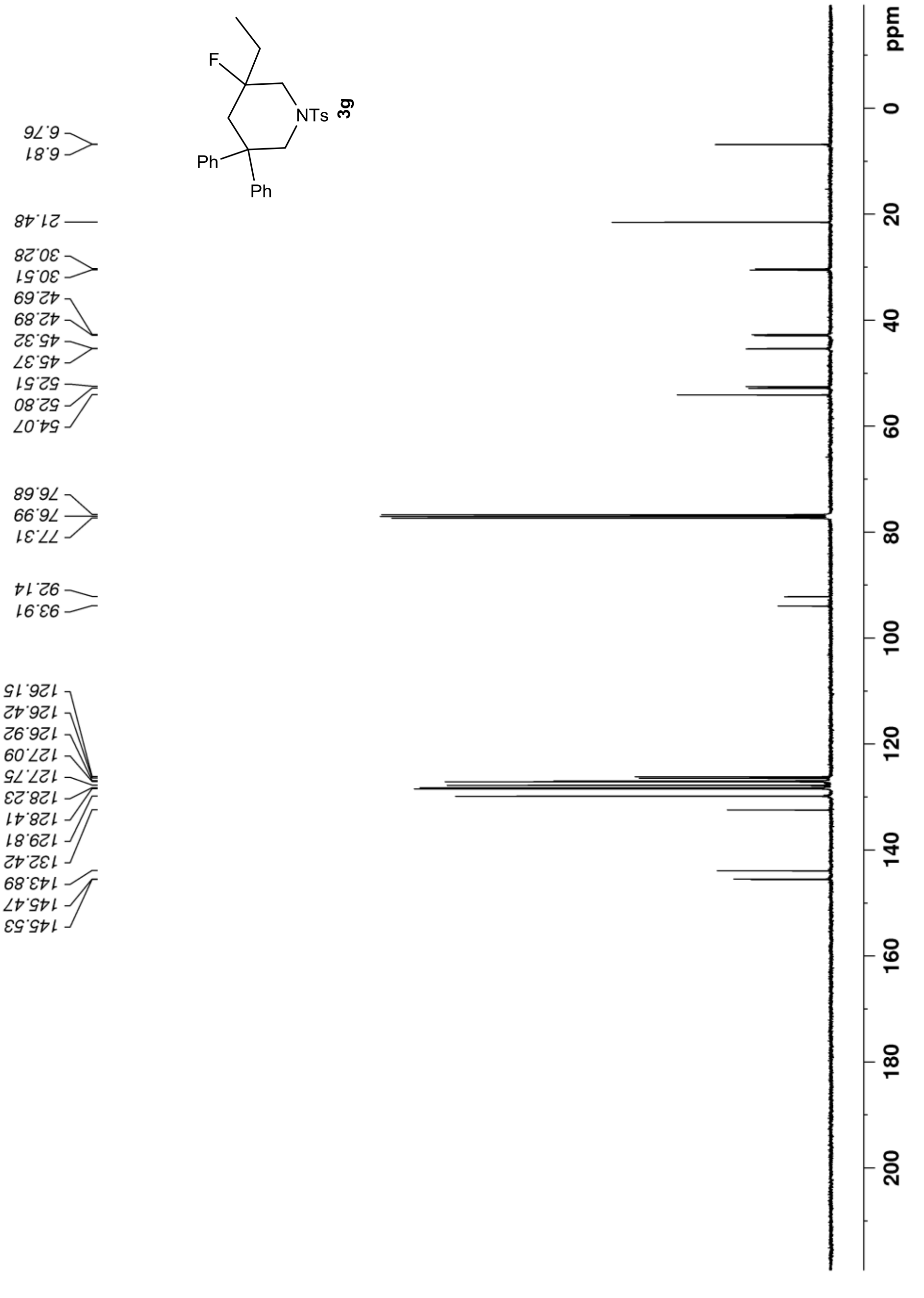
F
NTs
3g
Ph
Ph
6.76
6.81
21.48
30.28
30.51
42.69
42.89
45.32
45.37
52.51
52.80
54.07
76.68
76.99
77.31
92.14
93.91
126.15
126.42
126.92
127.09
127.75
128.23
128.41
129.81
132.42
143.89
145.47
145.53
ppm
0
20
40
60
80
100
120
140
160
180
200

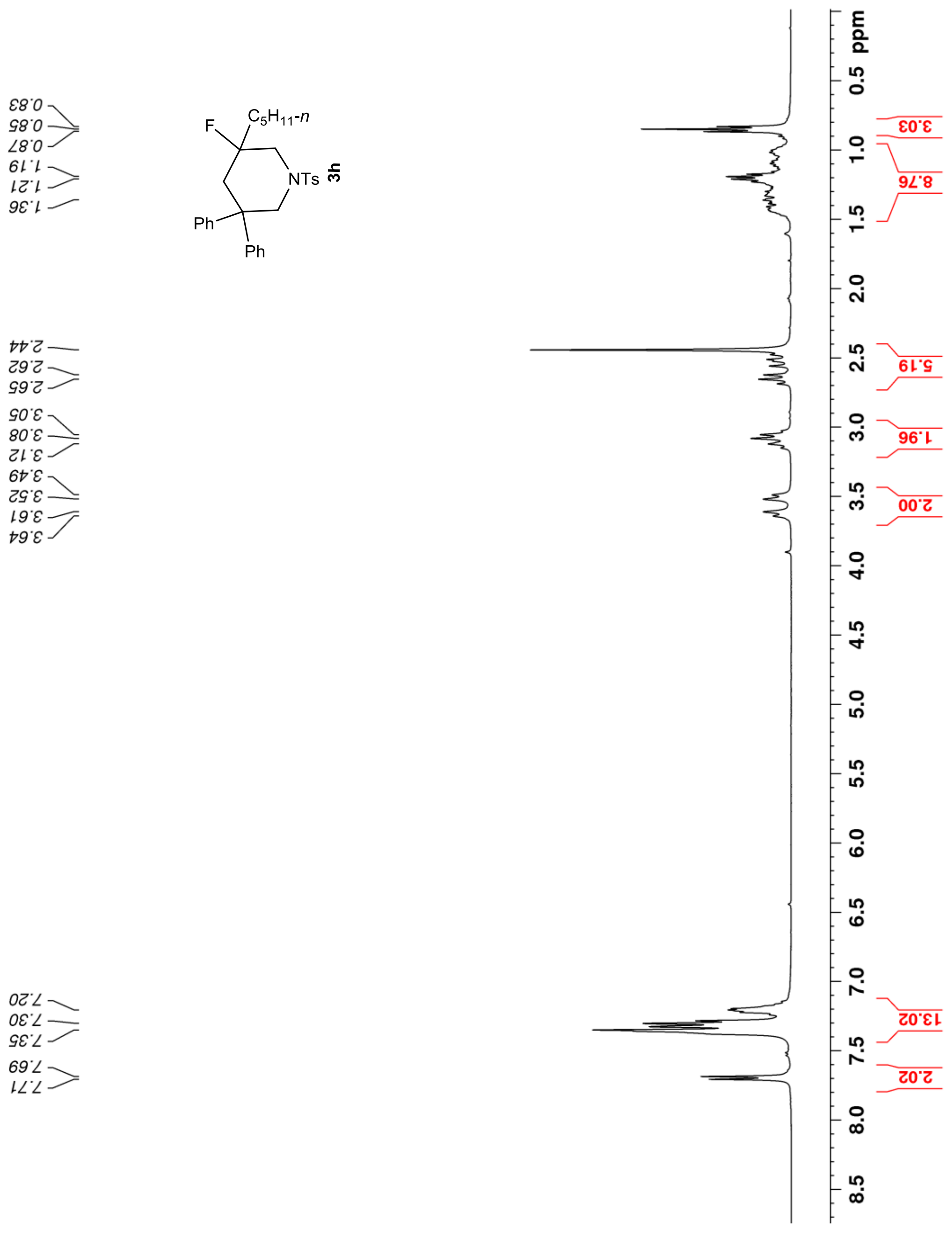

0.83
0.85
0.87
1.19
1.21
1.36
2.44
2.62
2.65
3.05
3.08
3.12
3.49
3.52
3.61
3.64
7.20
7.30
7.35
7.69
7.71
F
C_5H_{11}-n
NTs
3h
Ph
Ph
3.03
8.76
5.19
1.96
2.00
13.02
2.02
ppm

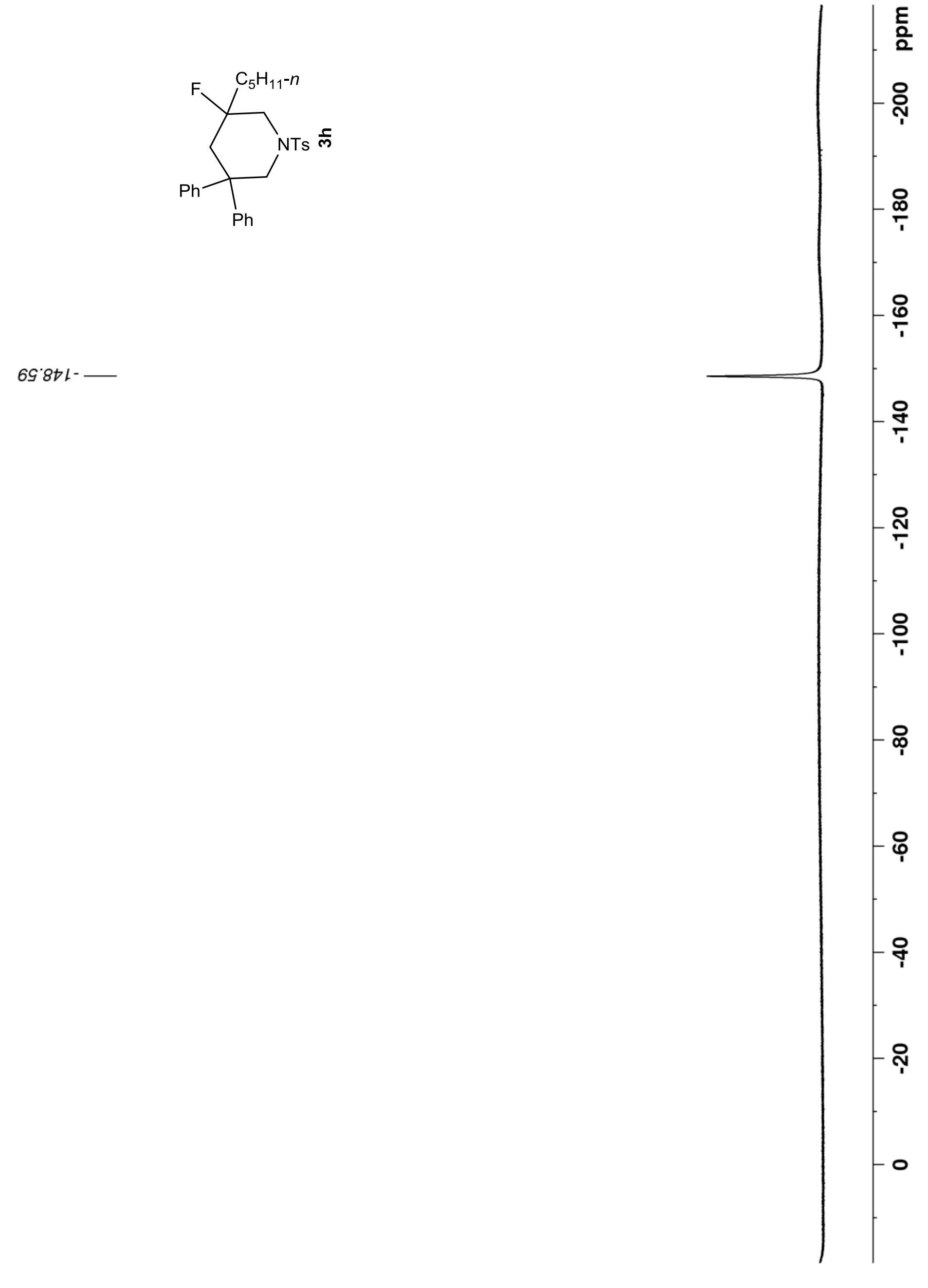
3h
-148.59
ppm
-200
-180
-160
-140
-120
-100
-80
-60
-40
-20
0

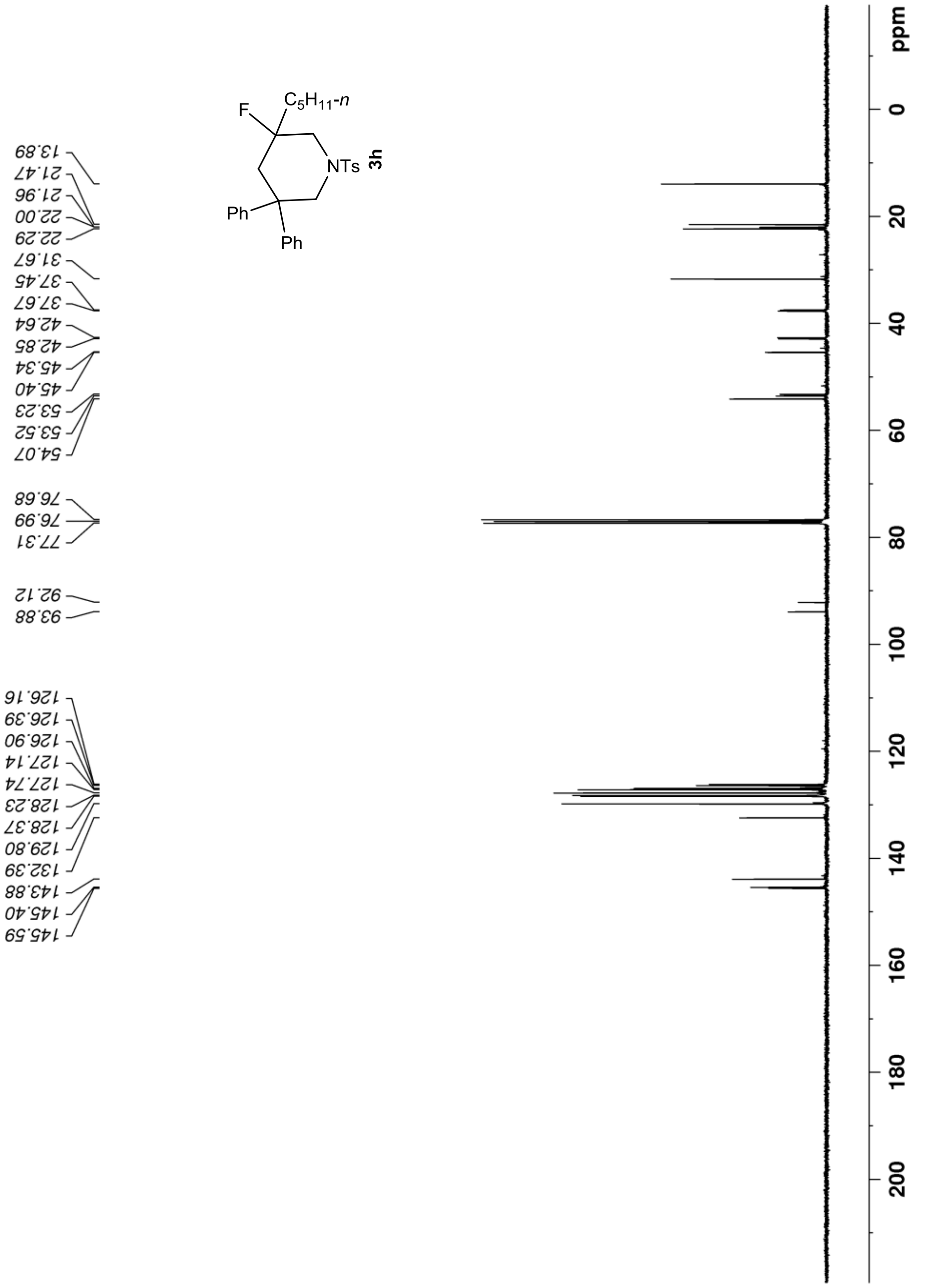
F
C_5H_{11}-n
NTs
Ph
Ph
3h
13.89
21.47
21.96
22.00
22.29
31.67
37.45
37.67
42.64
42.85
45.34
45.40
53.23
53.52
54.07
76.68
76.99
77.31
92.12
93.88
126.16
126.39
126.90
127.14
127.74
128.23
128.37
129.80
132.39
143.88
145.40
145.59
ppm
0
20
40
60
80
100
120
140
160
180
200

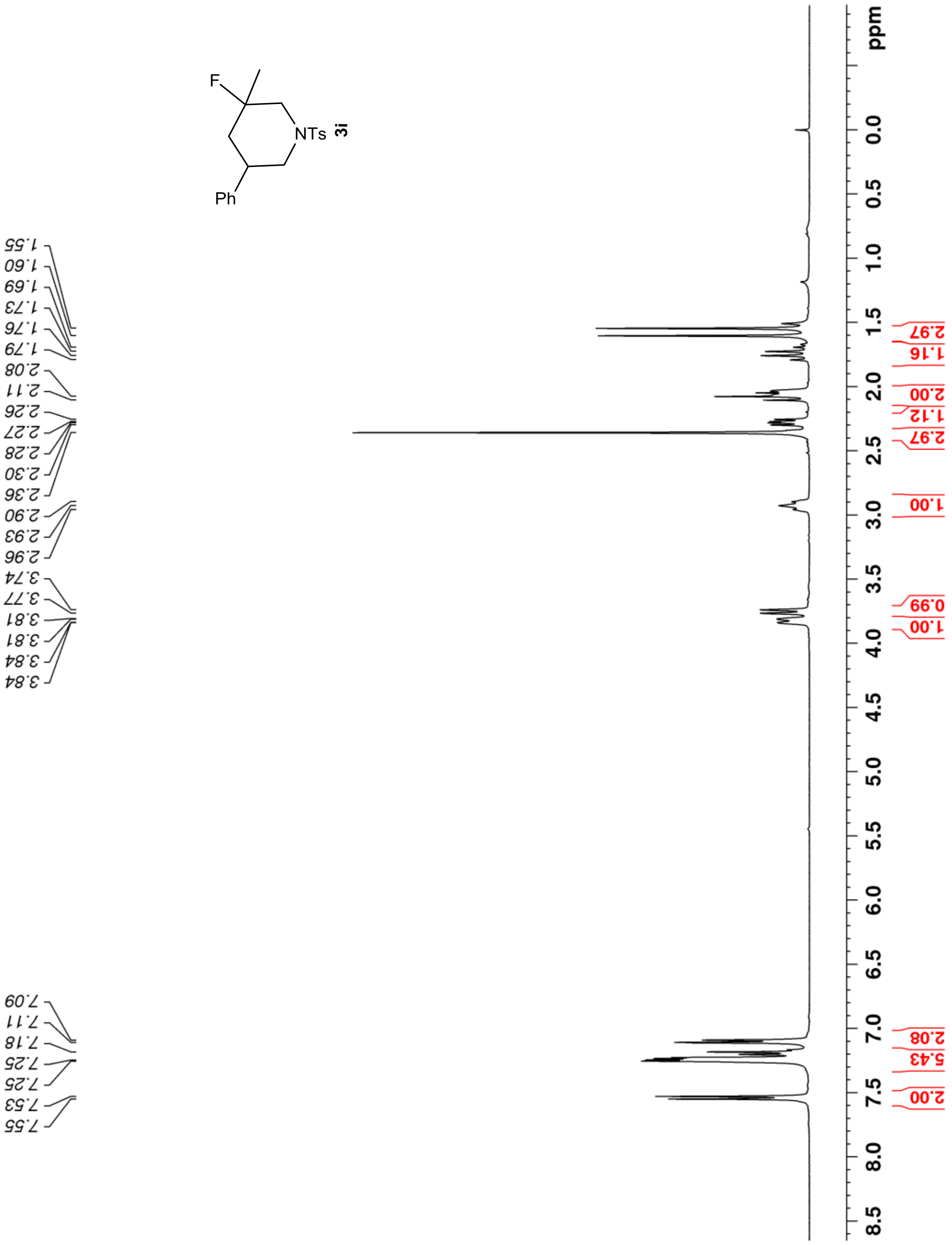
F
NTs
3i
Ph
1.55
1.60
1.69
1.73
1.76
1.79
2.08
2.11
2.26
2.27
2.28
2.30
2.36
2.90
2.93
2.96
3.74
3.77
3.81
3.81
3.84
3.84
7.09
7.11
7.18
7.25
7.25
7.53
7.55
2.97
1.16
2.00
1.12
2.97
1.00
0.99
1.00
2.08
5.43
2.00
ppm

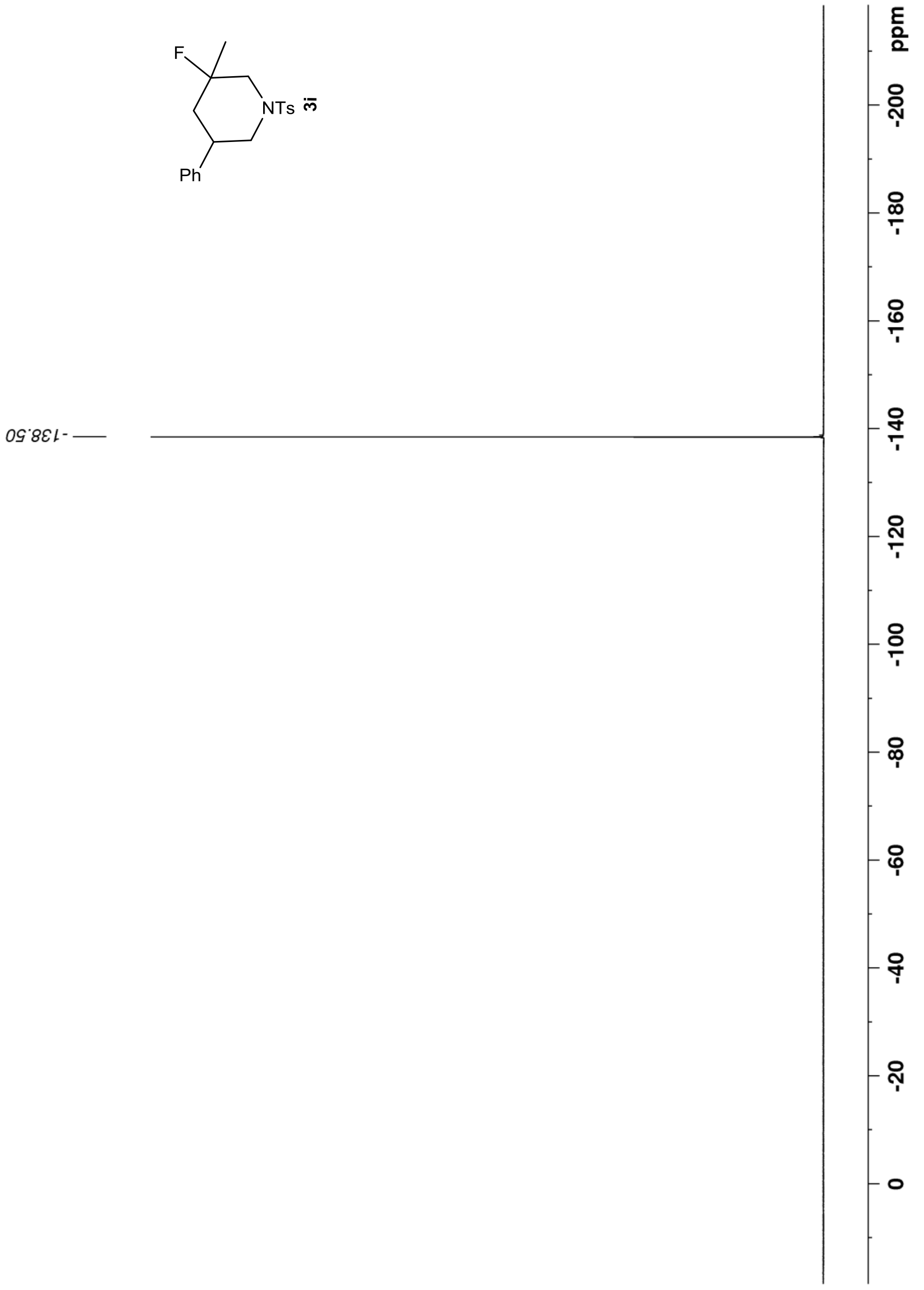
F
NTs
3i
Ph
-138.50
ppm
-200
-180
-160
-140
-120
-100
-80
-60
-40
-20
0

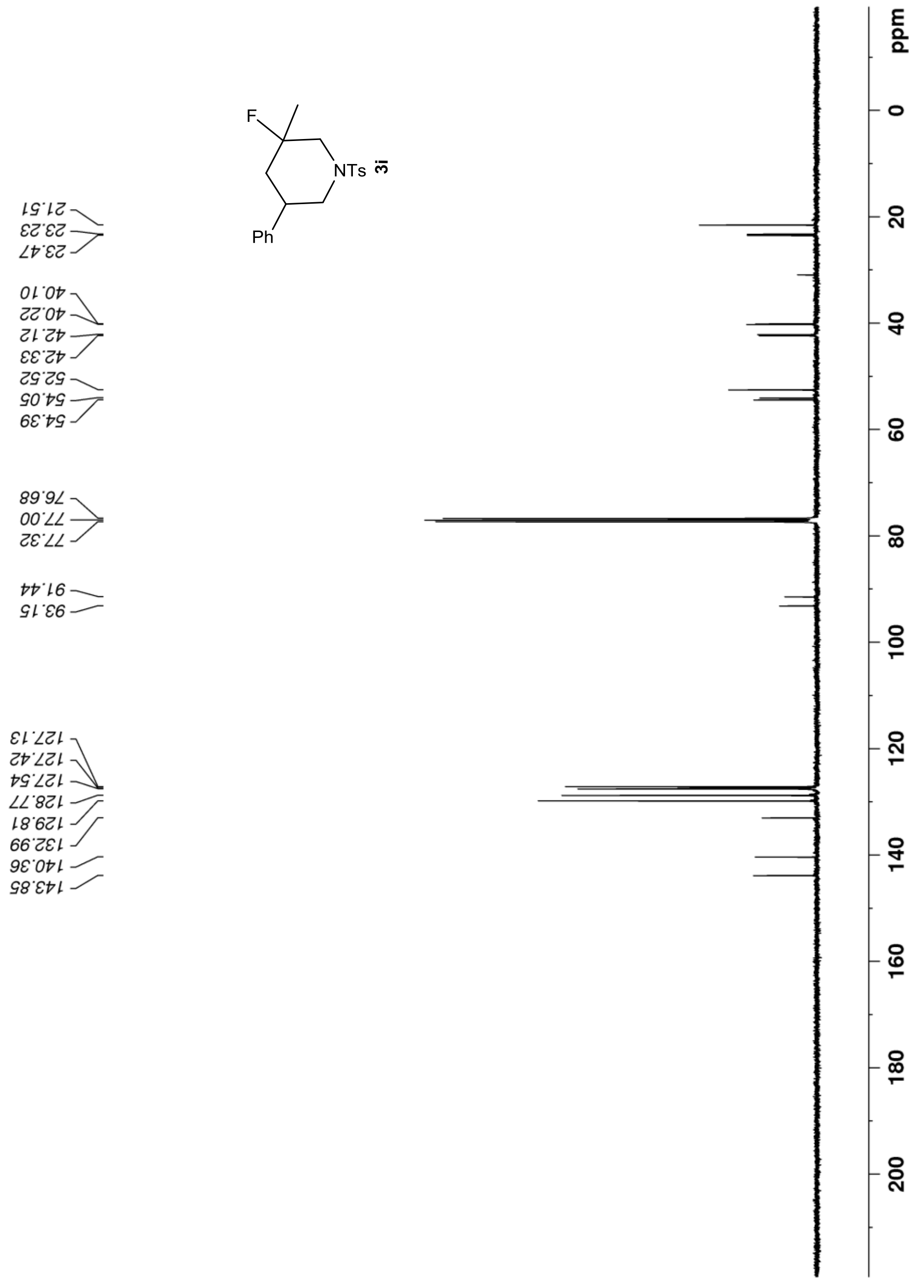
21.51
23.23
23.47
40.10
40.22
42.12
42.33
52.52
54.05
54.39
76.68
77.00
77.32
91.44
93.15
127.13
127.42
127.54
128.77
129.81
132.99
140.36
143.85
F
NTs
3i
Ph
ppm
0
20
40
60
80
100
120
140
160
180
200

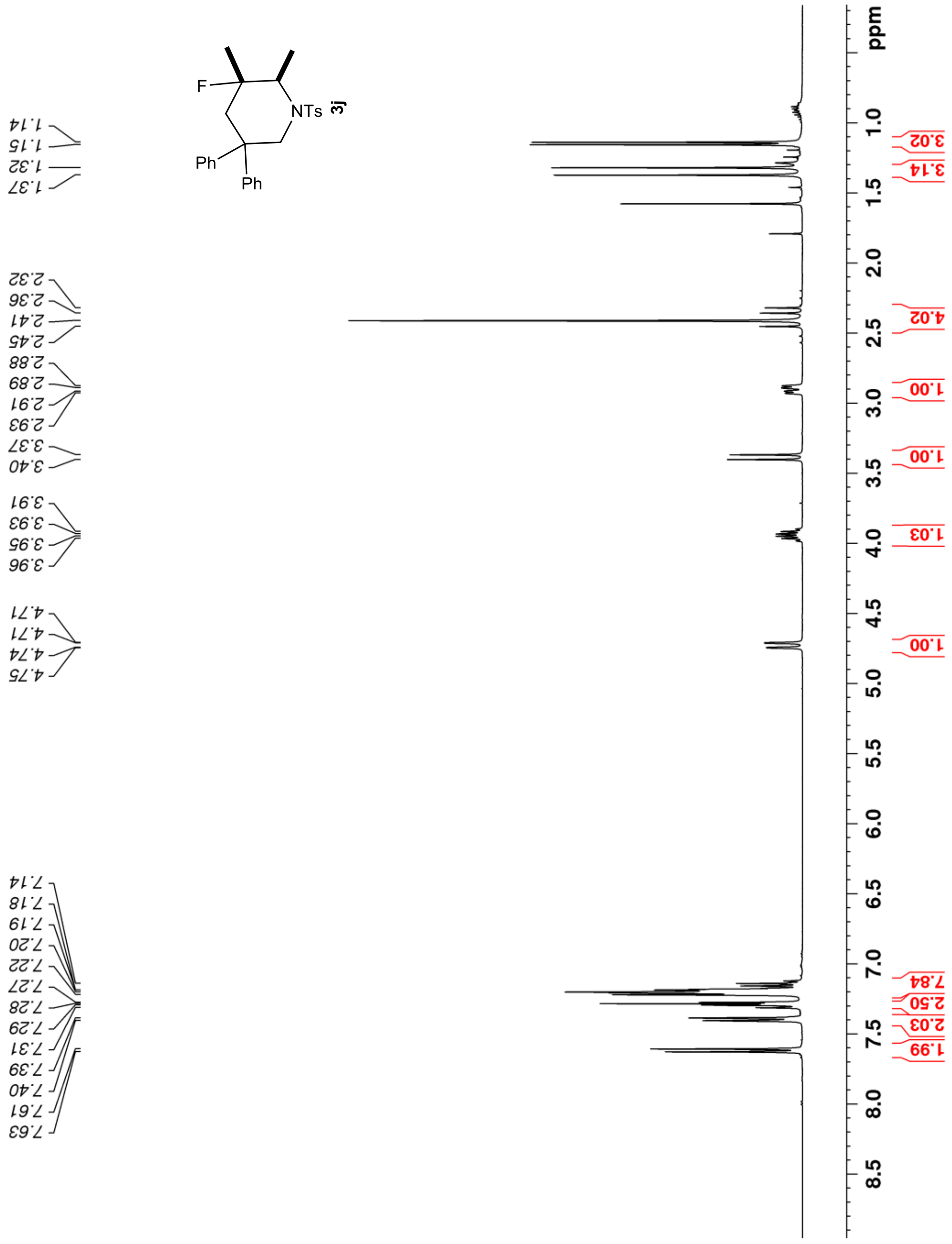
F
NTs
3j
Ph
Ph
ppm
1.14
1.15
1.32
1.37
2.32
2.36
2.41
2.45
2.88
2.89
2.91
2.93
3.37
3.40
3.91
3.93
3.95
3.96
4.71
4.71
4.74
4.75
7.14
7.18
7.19
7.20
7.22
7.27
7.28
7.29
7.31
7.39
7.40
7.61
7.63
3.02
3.14
4.02
1.00
1.00
1.03
1.00
7.84
2.50
2.03
1.99

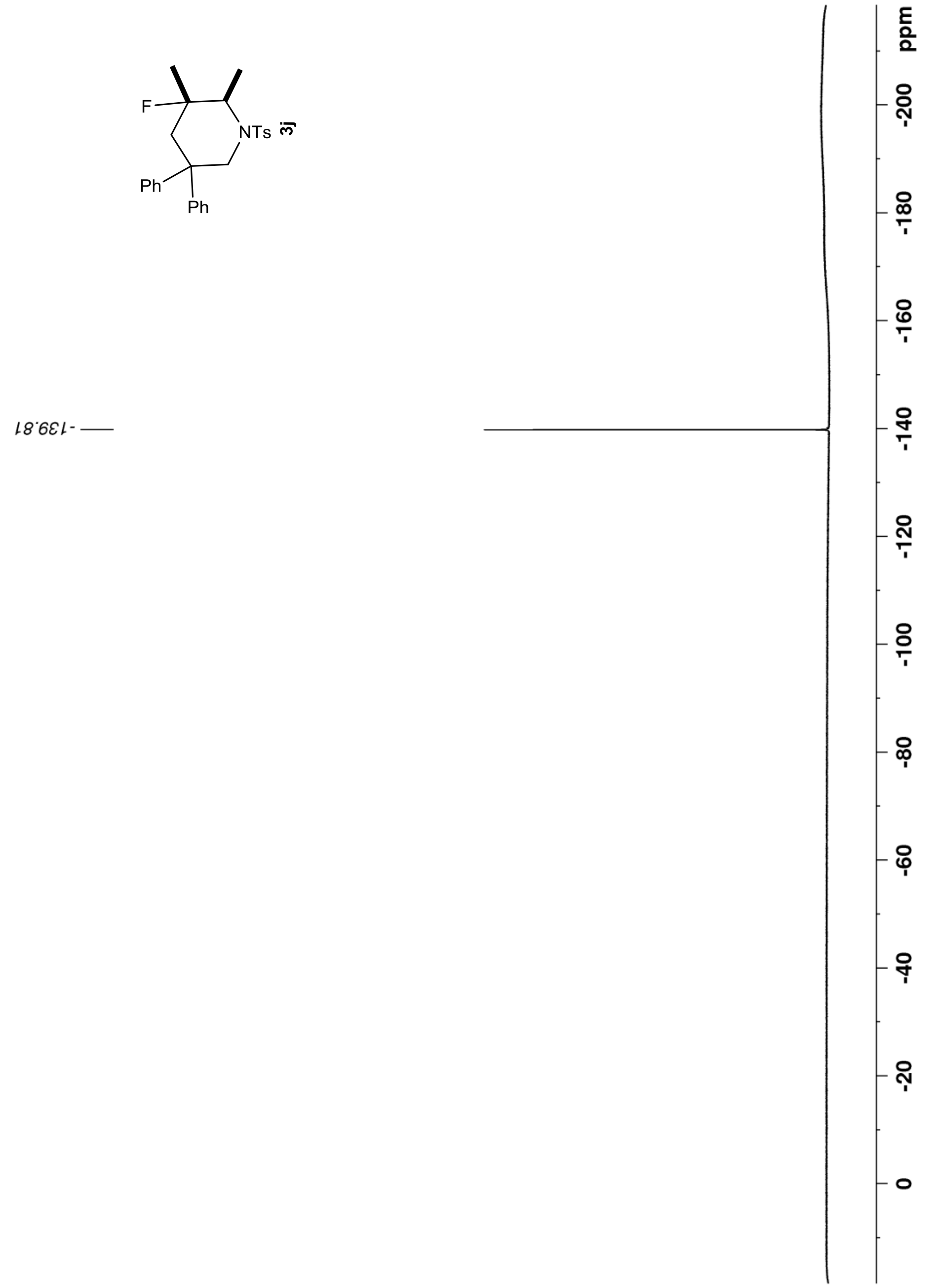

F
NTs
3j
Ph
Ph
-139.81
ppm
-200
-180
-160
-140
-120
-100
-80
-60
-40
-20
0

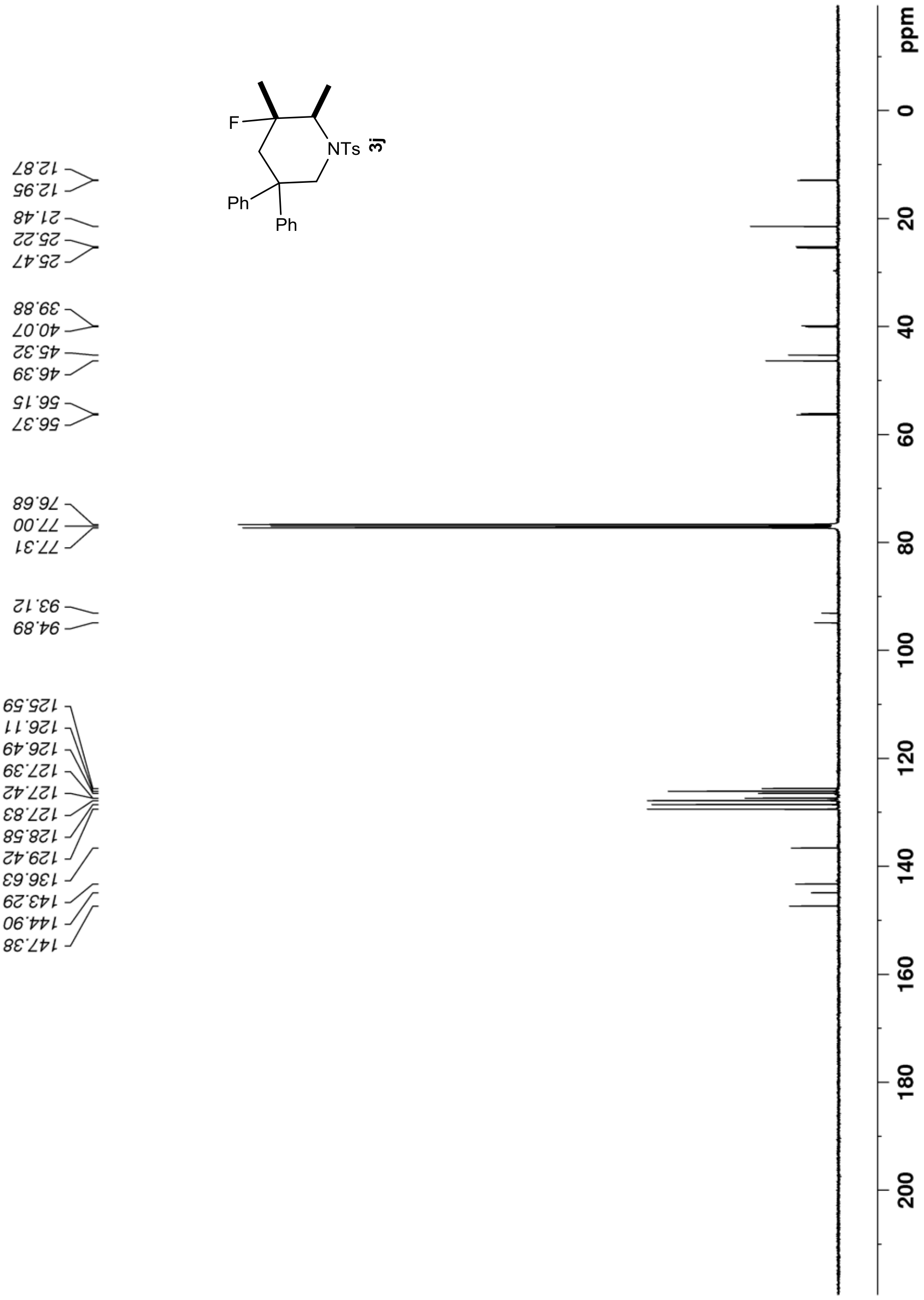
F
NTs
3j
Ph
Ph
12.87
12.95
21.48
25.22
25.47
39.88
40.07
45.32
46.39
56.15
56.37
76.68
77.00
77.31
93.12
94.89
125.59
126.11
126.49
127.39
127.42
127.83
128.58
129.42
136.63
143.29
144.90
147.38
ppm
0
20
40
60
80
100
120
140
160
180
200

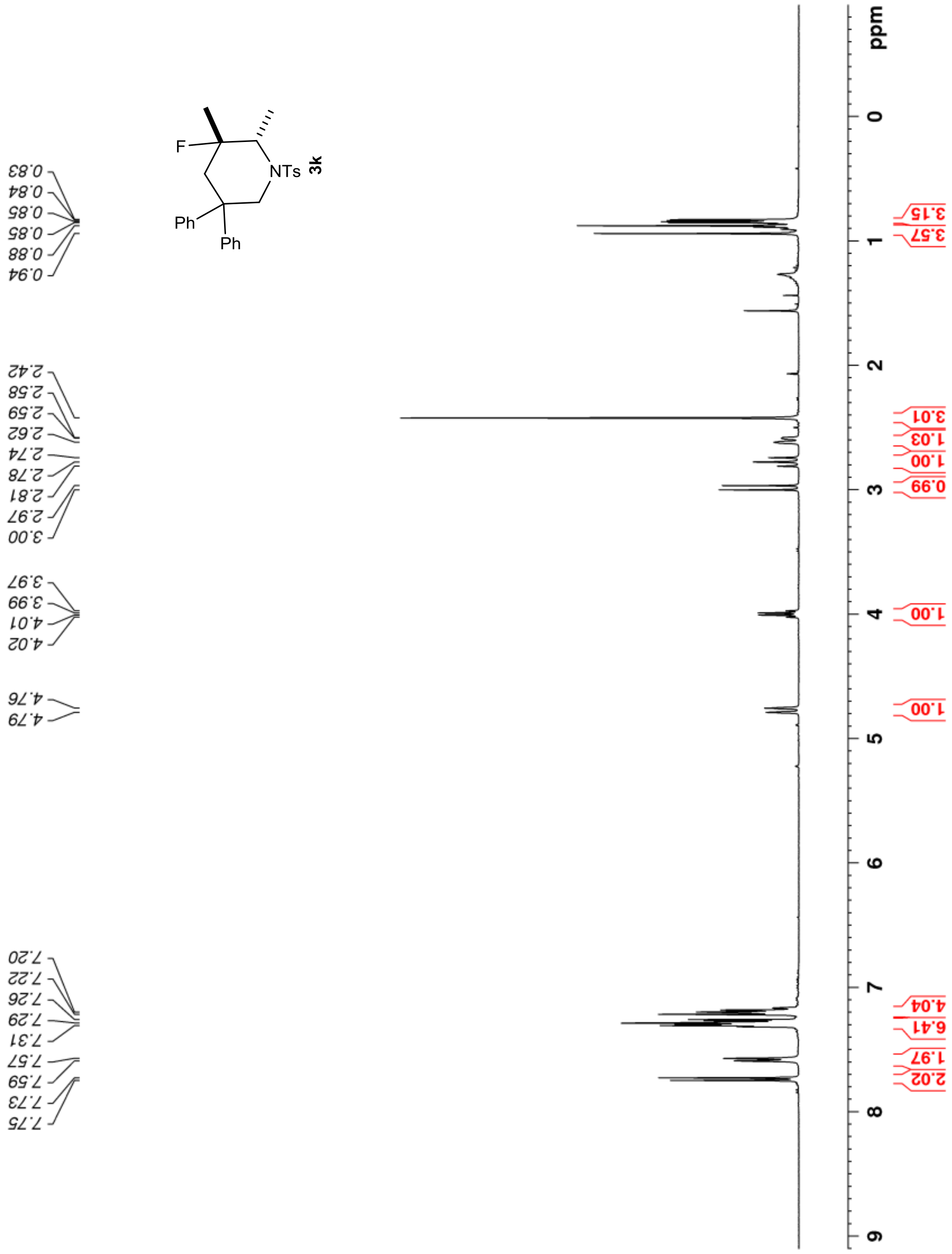

F
NTs
3k
Ph
Ph
0.83
0.84
0.85
0.85
0.88
0.94
2.42
2.58
2.59
2.62
2.74
2.78
2.81
2.97
3.00
3.97
3.99
4.01
4.02
4.76
4.79
7.20
7.22
7.26
7.29
7.31
7.57
7.59
7.73
7.75
3.15
3.57
3.01
1.03
1.00
0.99
1.00
1.00
4.04
6.41
1.97
2.02
ppm
0
1
2
3
4
5
6
7
8
9

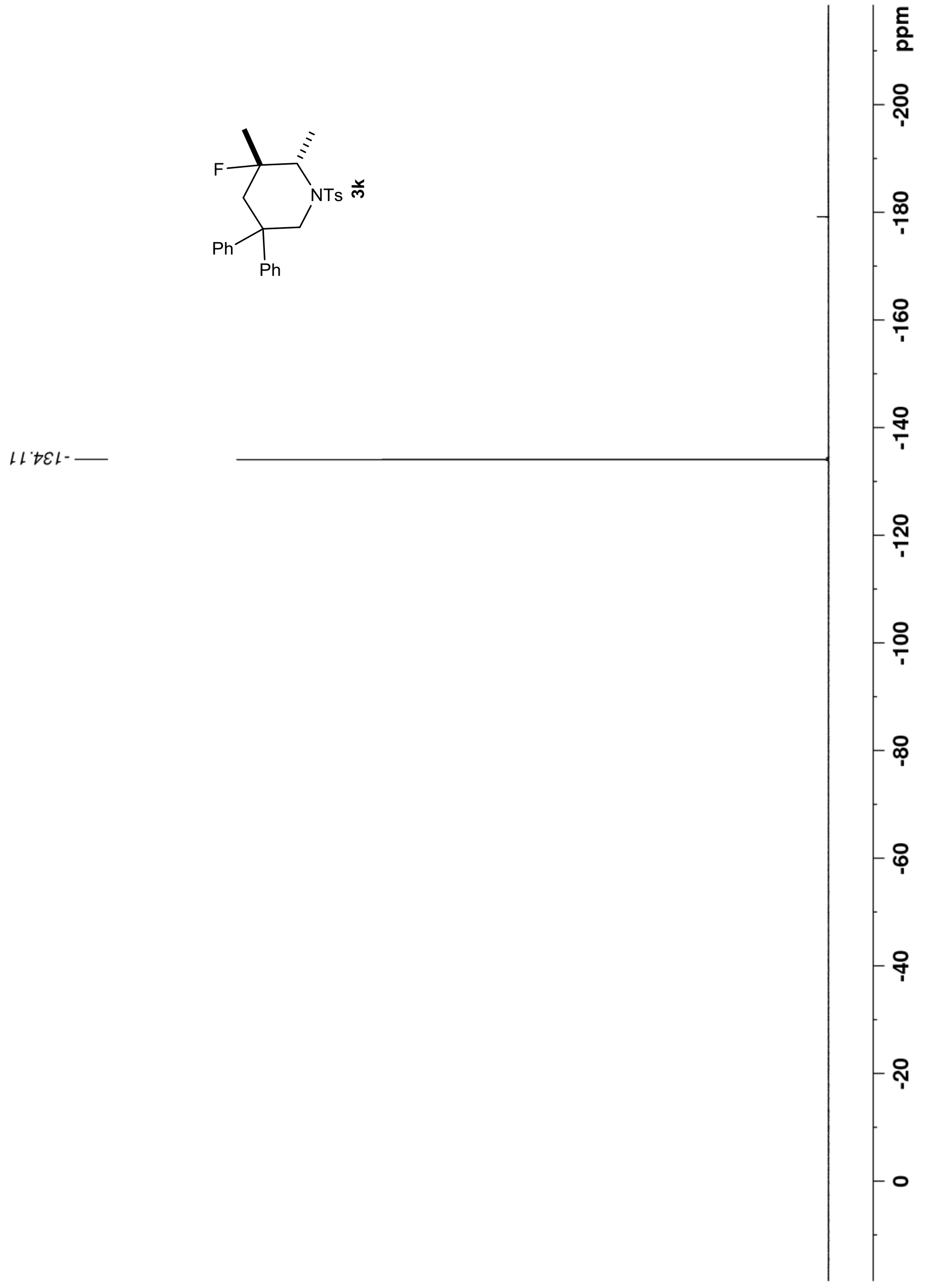

-134.11
F
NTs
3k
Ph
Ph
ppm
-200
-180
-160
-140
-120
-100
-80
-60
-40
-20
0

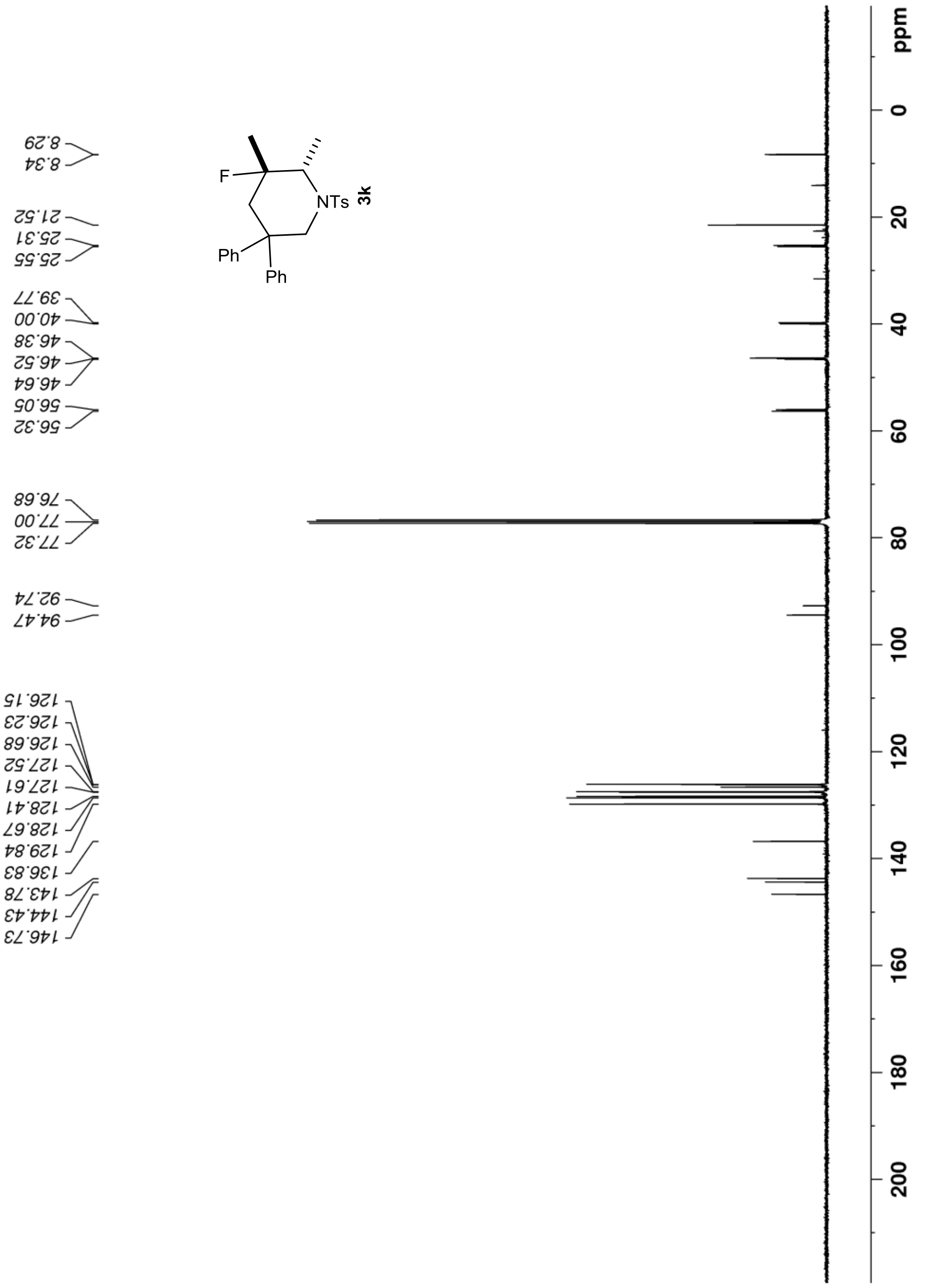

8.29
8.34
21.52
25.31
25.55
39.77
40.00
46.38
46.52
46.64
56.05
56.32
76.68
77.00
77.32
92.74
94.47
126.15
126.23
126.68
127.52
127.61
128.41
128.67
129.84
136.83
143.78
144.43
146.73
F
NTs
3k
Ph
Ph
ppm
0
20
40
60
80
100
120
140
160
180
200

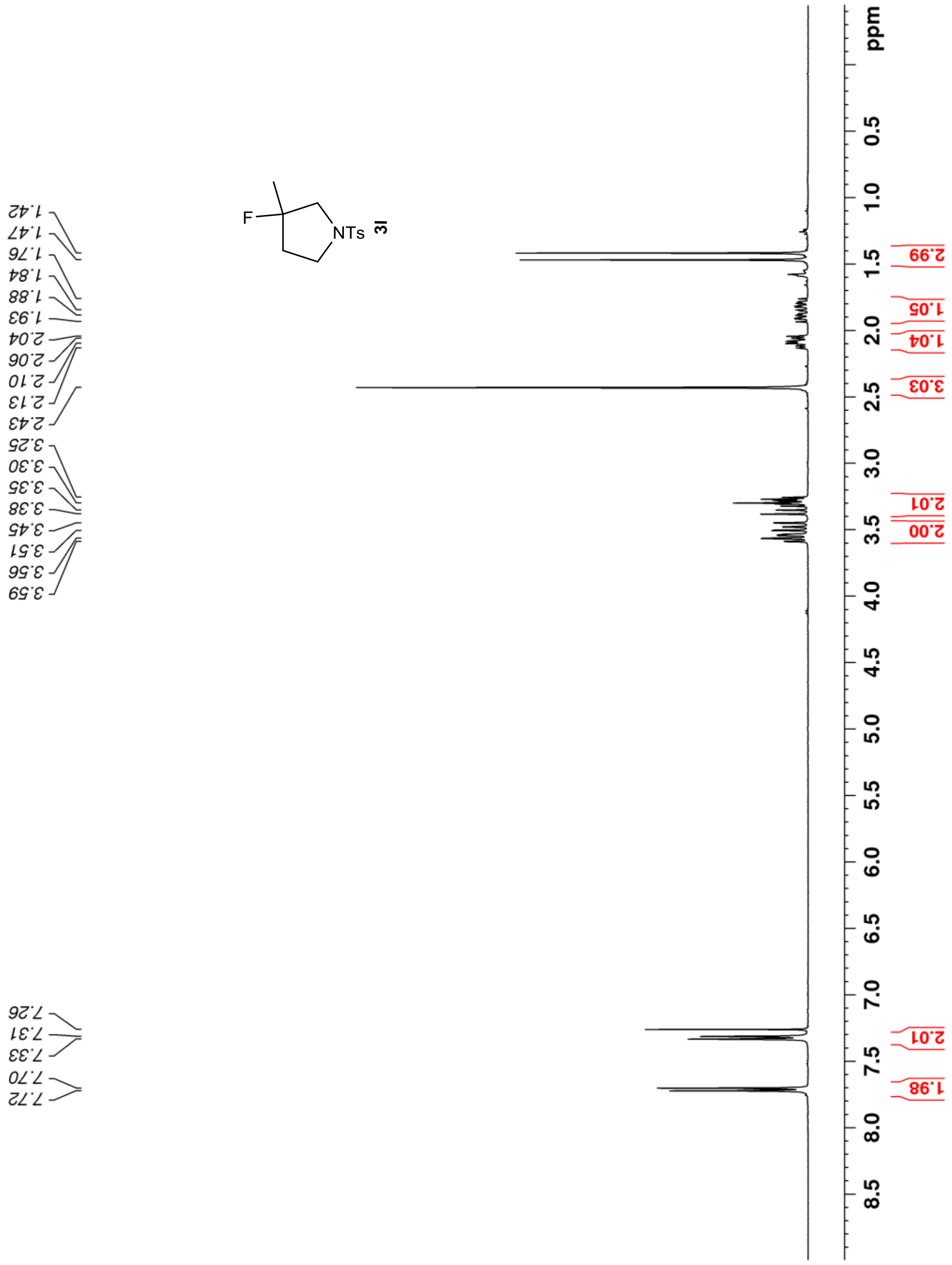
1.42
1.47
1.76
1.84
1.88
1.93
2.04
2.06
2.10
2.13
2.43
3.25
3.30
3.35
3.38
3.45
3.51
3.56
3.59
7.26
7.31
7.33
7.70
7.72
F
NTs
3l
2.99
1.05
1.04
3.03
2.01
2.00
2.01
1.98
ppm
0.5
1.0
1.5
2.0
2.5
3.0
3.5
4.0
4.5
5.0
5.5
6.0
6.5
7.0
7.5
8.0
8.5

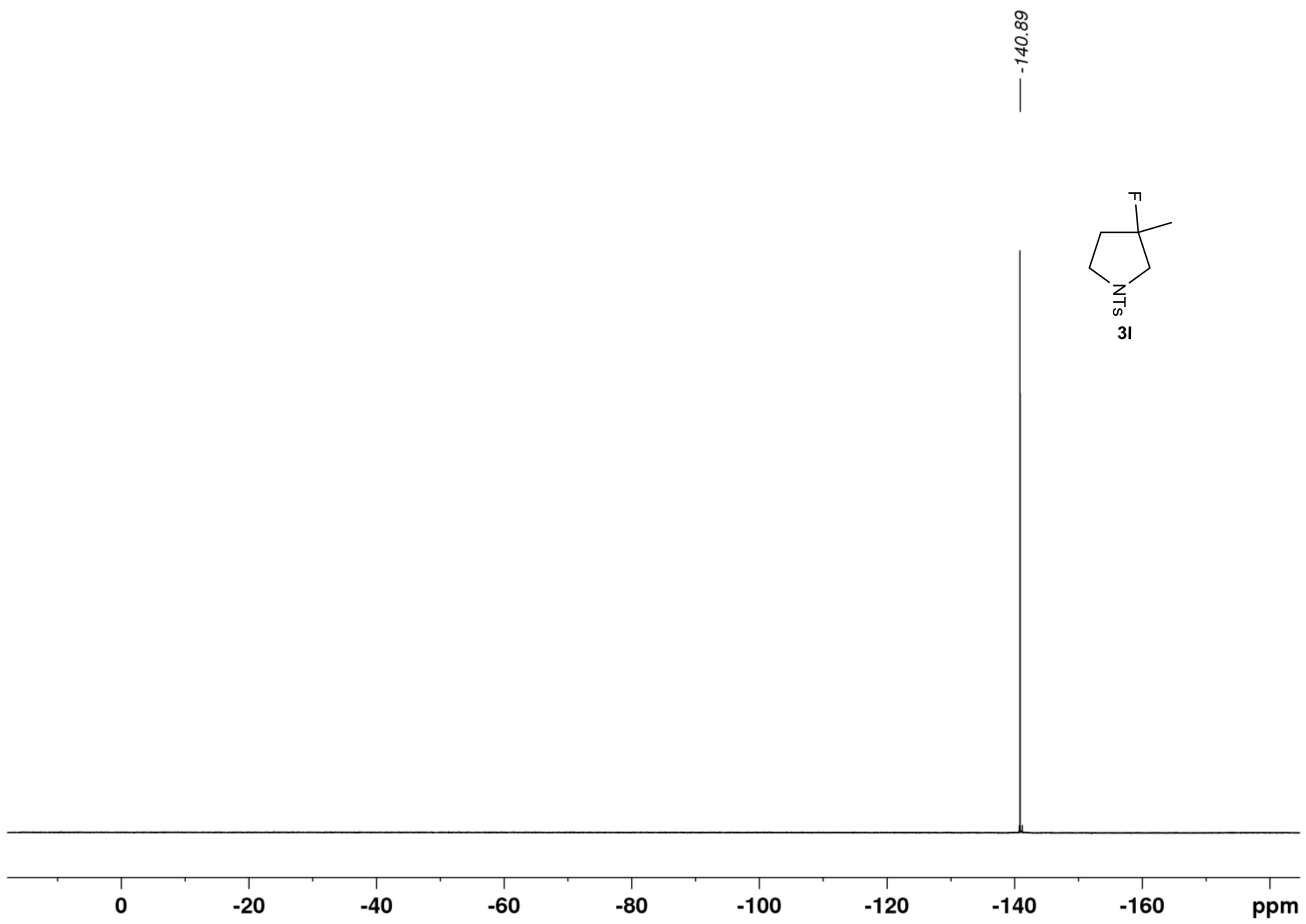
-140.89
F
NTs
3l
0
-20
-40
-60
-80
-100
-120
-140
-160
ppm

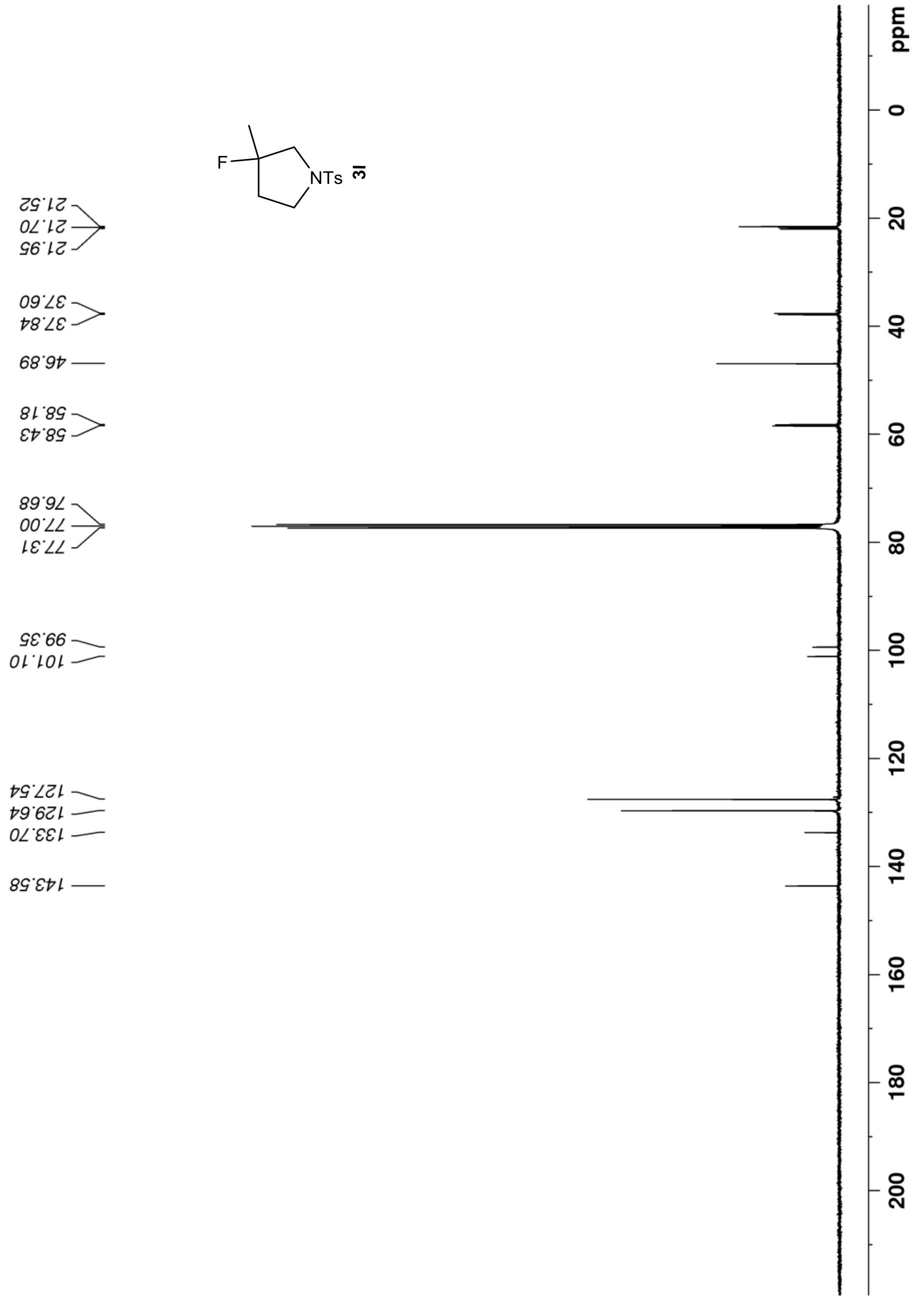

F
NTs
3l
21.52
21.70
21.95
37.60
37.84
46.89
58.18
58.43
76.68
77.00
77.31
99.35
101.10
127.54
129.64
133.70
143.58
ppm
0
20
40
60
80
100
120
140
160
180
200

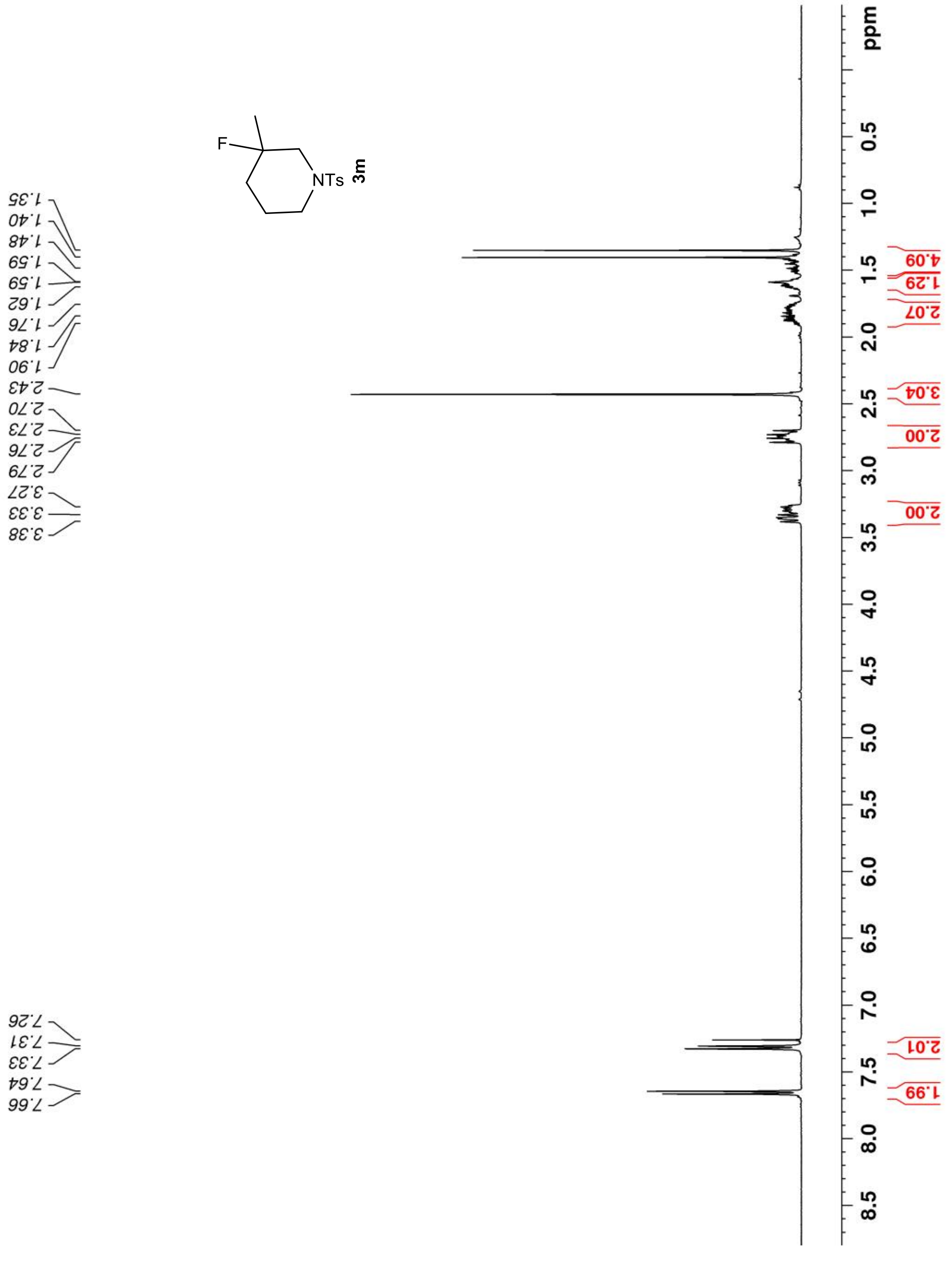
3m
NTs
F
1.35
1.40
1.48
1.59
1.59
1.62
1.76
1.84
1.90
2.43
2.70
2.73
2.76
2.79
3.27
3.33
3.38
7.26
7.31
7.33
7.64
7.66
4.09
1.29
2.07
3.04
2.00
2.00
2.01
1.99
ppm

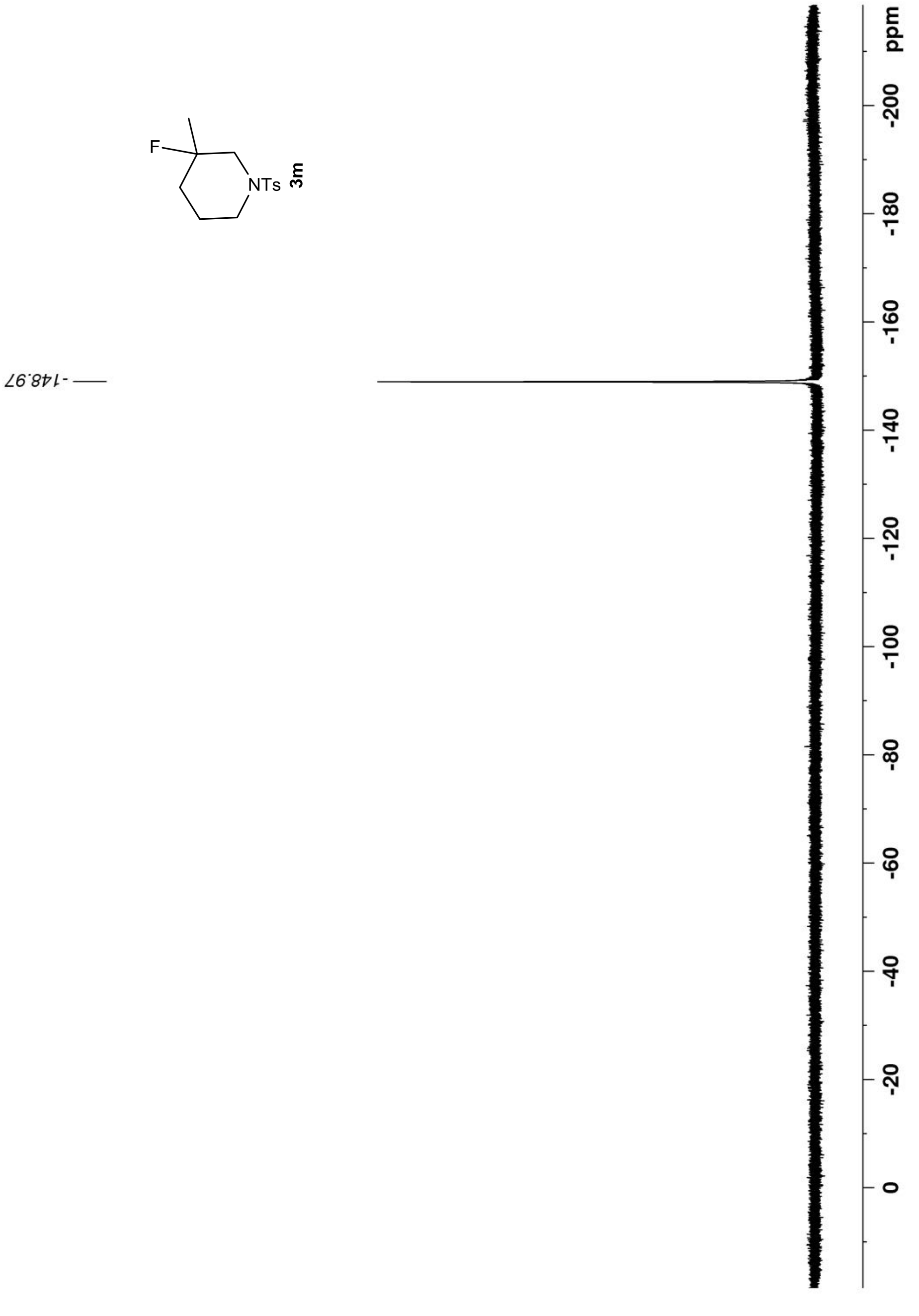
F
NTs
3m
-148.97
ppm
-200
-180
-160
-140
-120
-100
-80
-60
-40
-20
0

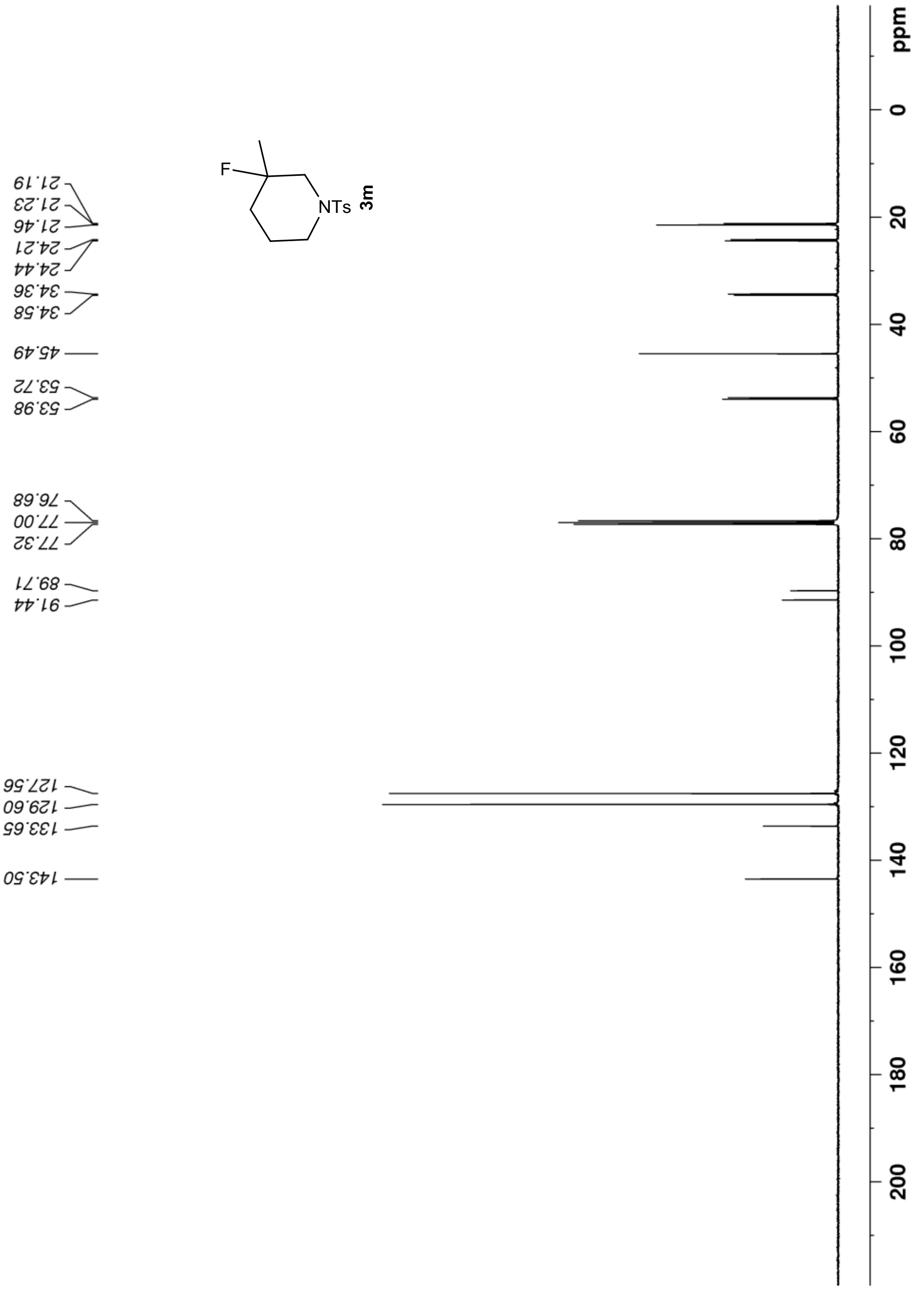
21.19
21.23
21.46
24.21
24.44
34.36
34.58
45.49
53.72
53.98
76.68
77.00
77.32
89.71
91.44
127.56
129.60
133.65
143.50
F
NTs
3m
ppm

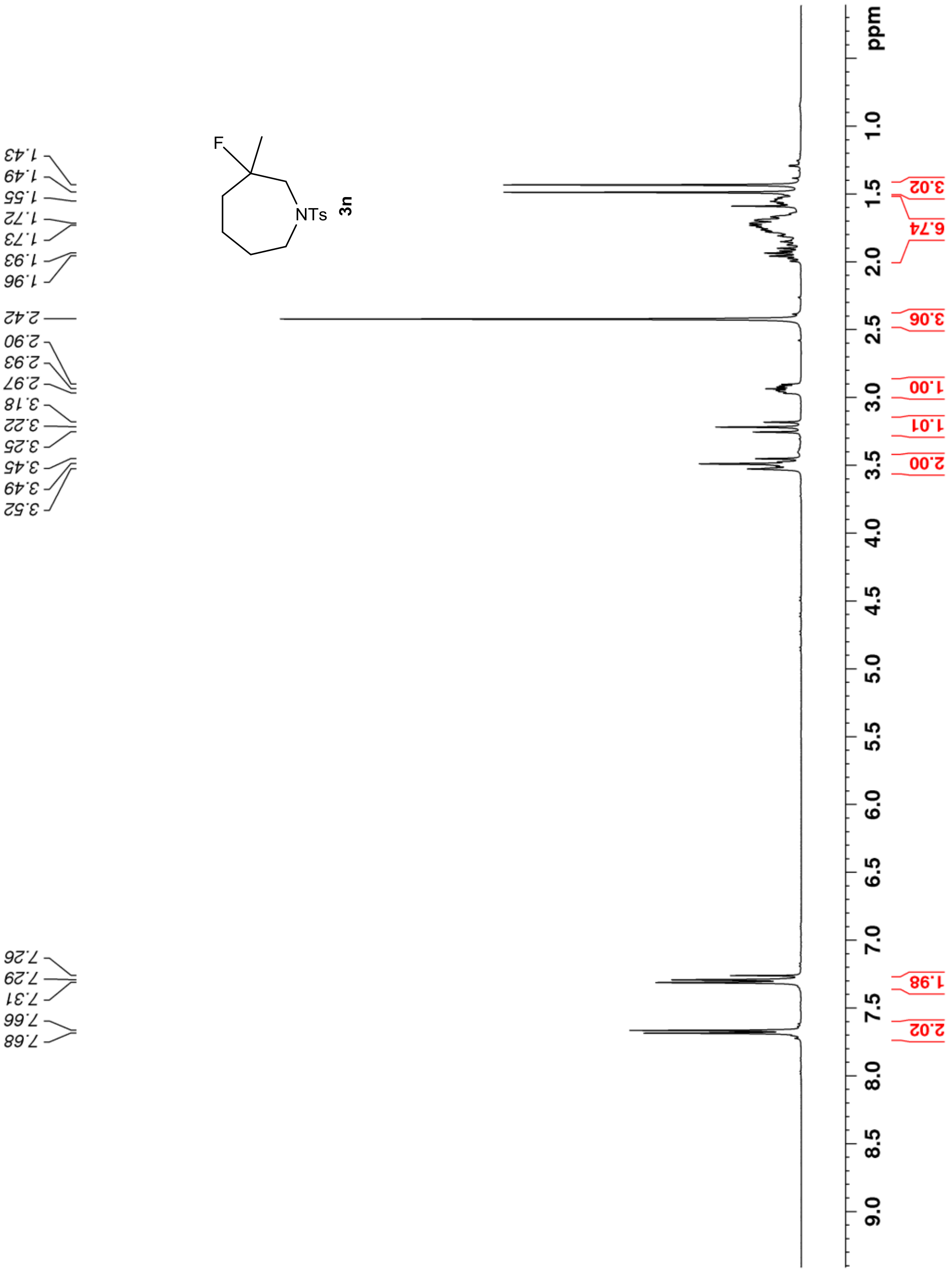
1.43
1.49
1.55
1.72
1.73
1.93
1.96
2.42
2.90
2.93
2.97
3.18
3.22
3.25
3.45
3.49
3.52
7.26
7.29
7.31
7.66
7.68
F
NTs
3n
3.02
6.74
3.06
1.00
1.01
2.00
1.98
2.02
ppm
1.0
1.5
2.0
2.5
3.0
3.5
4.0
4.5
5.0
5.5
6.0
6.5
7.0
7.5
8.0
8.5
9.0

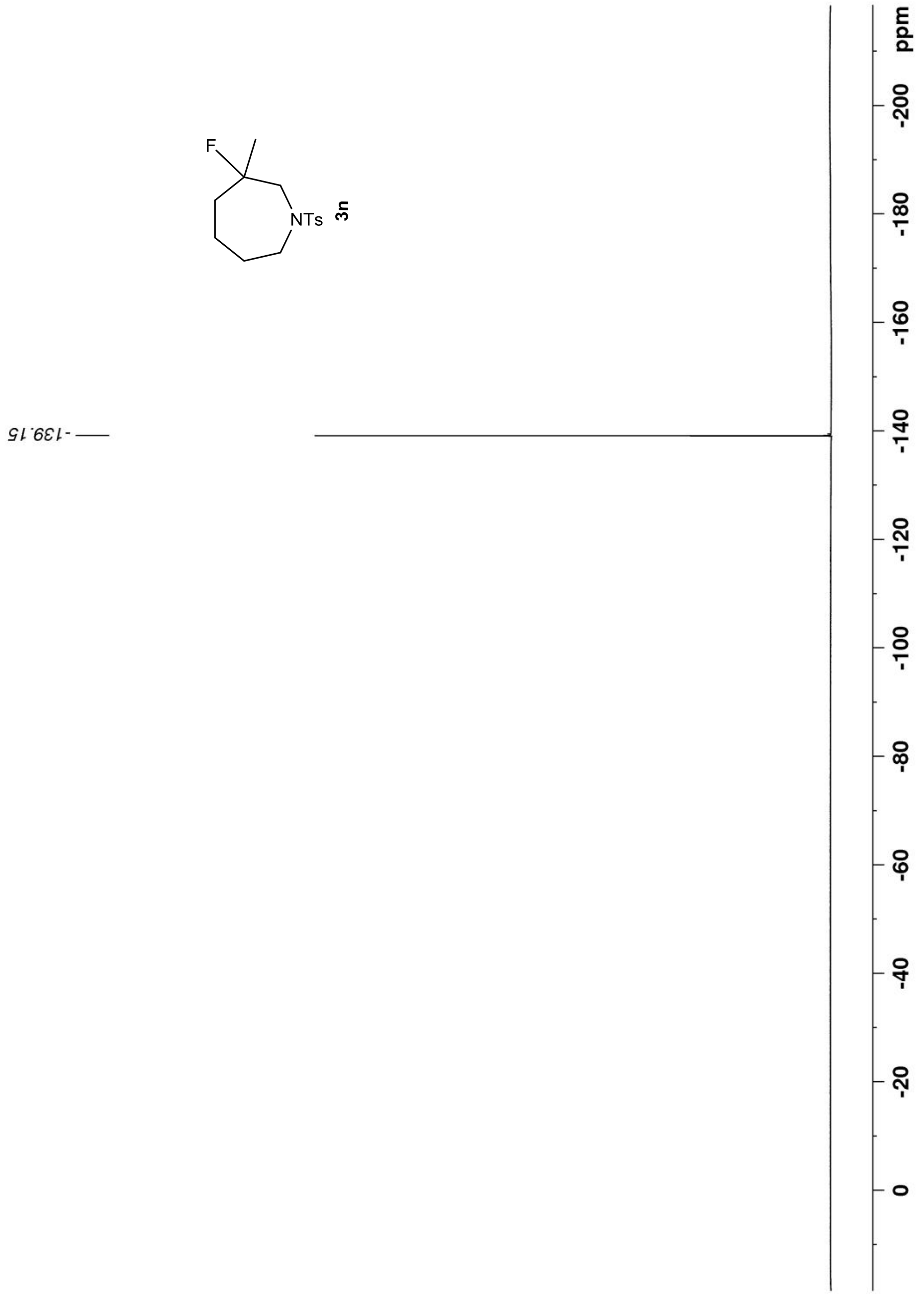
F
NTs
3n
-139.15
ppm
-200
-180
-160
-140
-120
-100
-80
-60
-40
-20
0

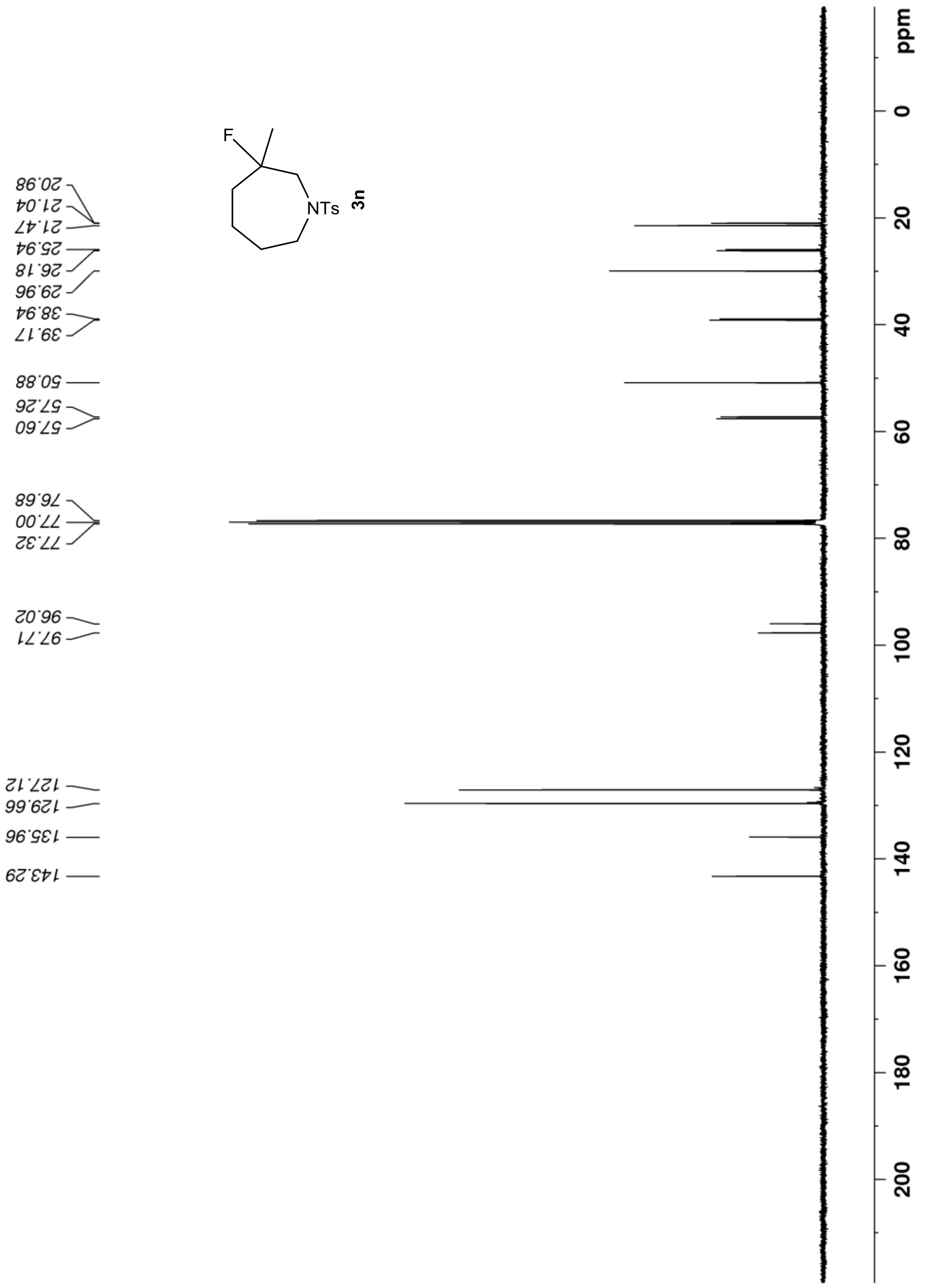

20.98
21.04
21.47
25.94
26.18
29.96
38.94
39.17
50.88
57.26
57.60
76.68
77.00
77.32
96.02
97.71
127.12
129.66
135.96
143.29
F
NTs
3n
ppm
0
20
40
60
80
100
120
140
160
180
200

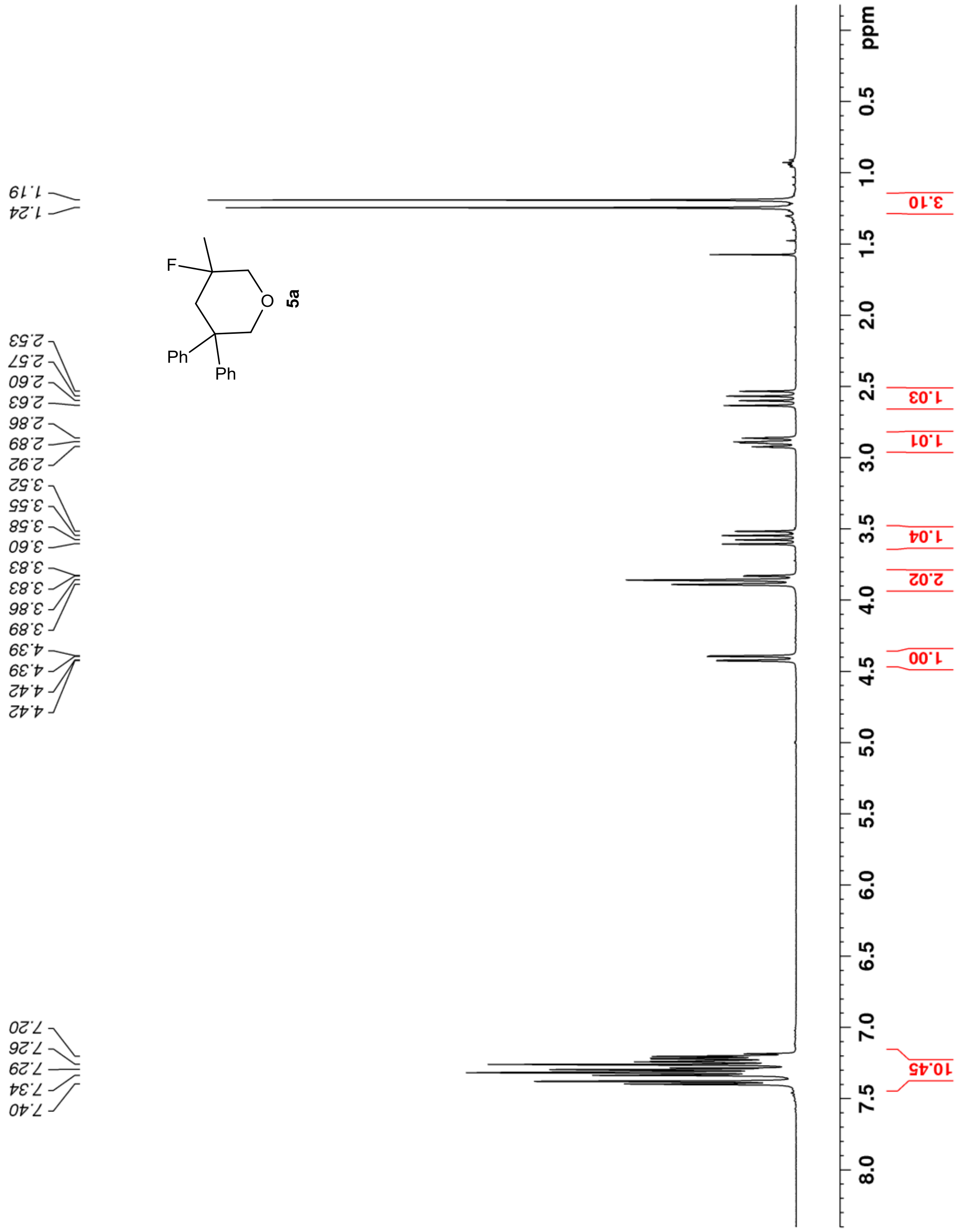
1.19
1.24
2.53
2.57
2.60
2.63
2.86
2.89
2.92
3.52
3.55
3.58
3.60
3.83
3.83
3.86
3.89
4.39
4.39
4.42
4.42
7.20
7.26
7.29
7.34
7.40
F
O
Ph
Ph
5a
3.10
1.03
1.01
1.04
2.02
1.00
10.45
ppm
0.5
1.0
1.5
2.0
2.5
3.0
3.5
4.0
4.5
5.0
5.5
6.0
6.5
7.0
7.5
8.0

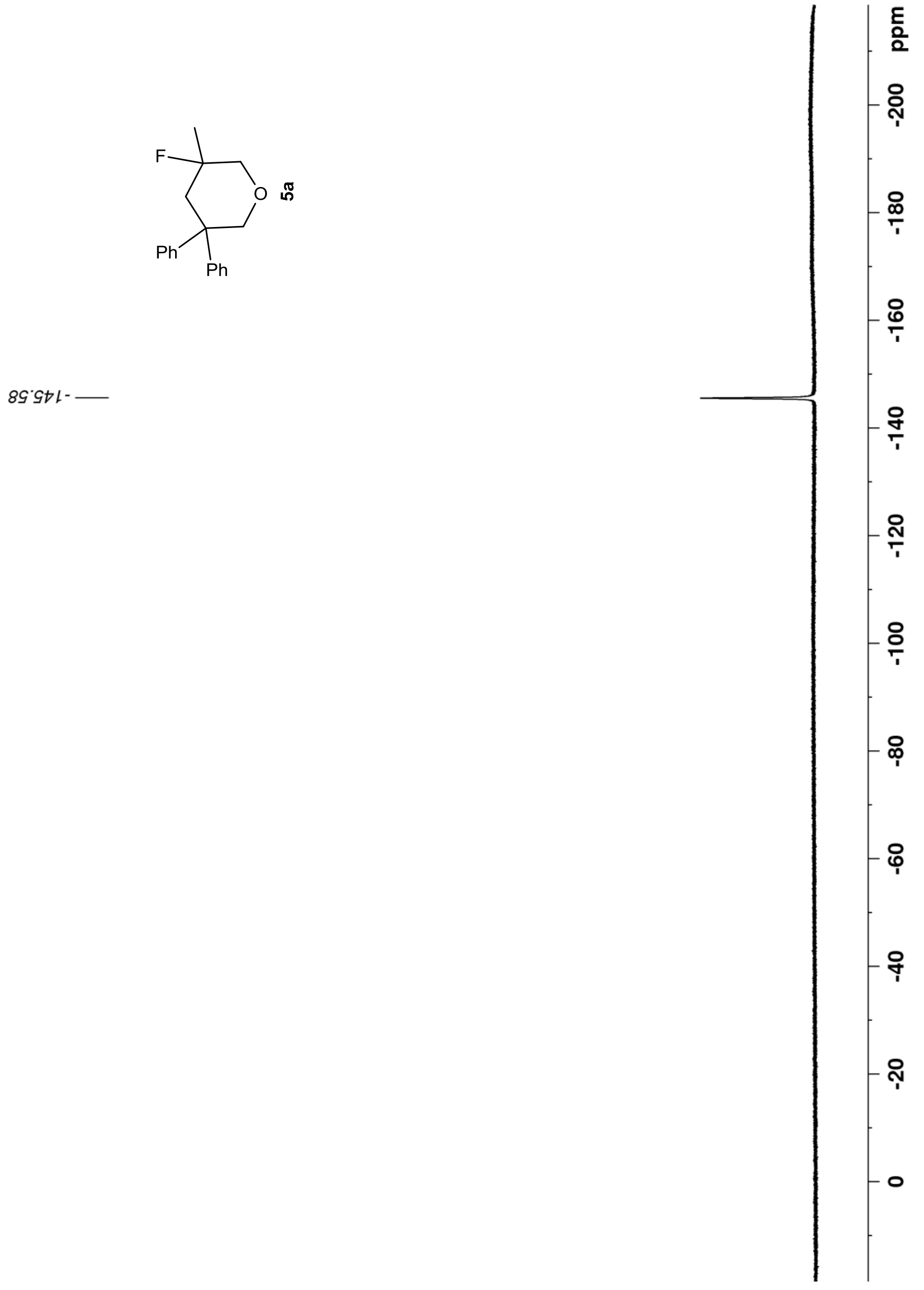
F
O
5a
Ph
Ph
-145.58
ppm
-200
-180
-160
-140
-120
-100
-80
-60
-40
-20
0

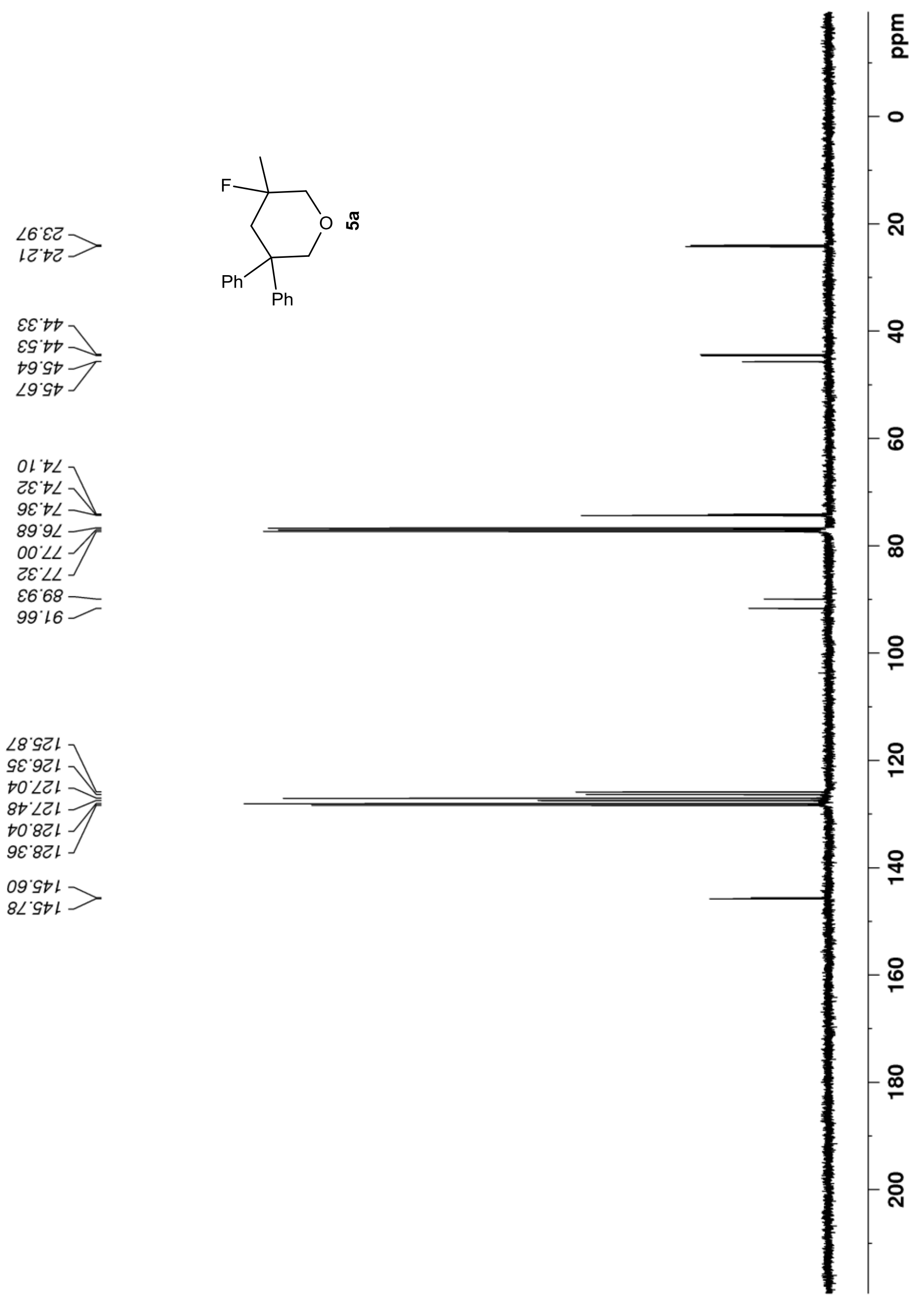
23.97
24.21
44.33
44.53
45.64
45.67
74.10
74.32
74.36
76.68
77.00
77.32
89.93
91.66
125.87
126.35
127.04
127.48
128.04
128.36
145.60
145.78
F
O
Ph
Ph
5a
ppm
0
20
40
60
80
100
120
140
160
180
200

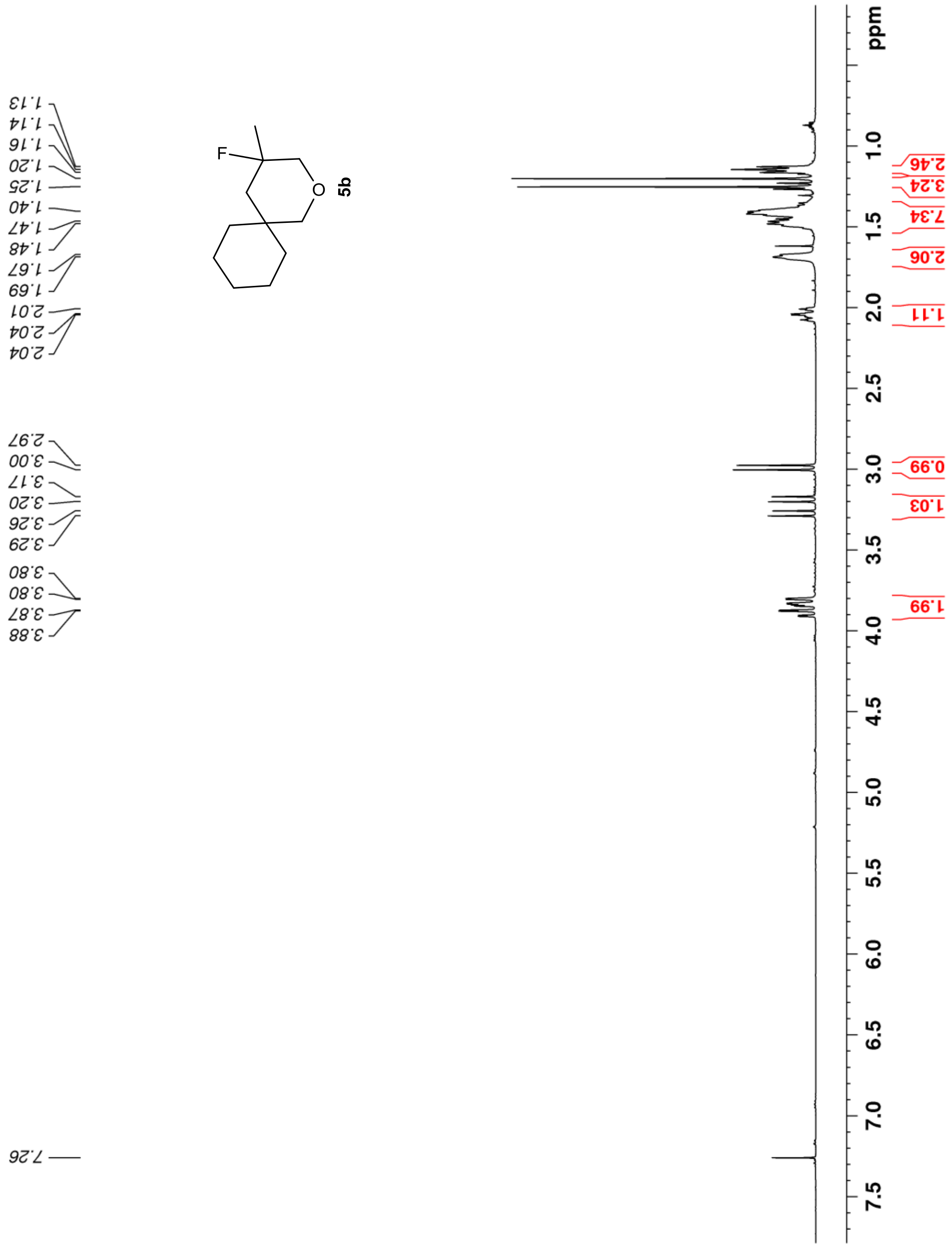

1.13
1.14
1.16
1.20
1.25
1.40
1.47
1.48
1.67
1.69
2.01
2.04
2.04
2.97
3.00
3.17
3.20
3.26
3.29
3.80
3.80
3.87
3.88
7.26
F
O
5b
2.46
3.24
7.34
2.06
1.11
0.99
1.03
1.99
ppm

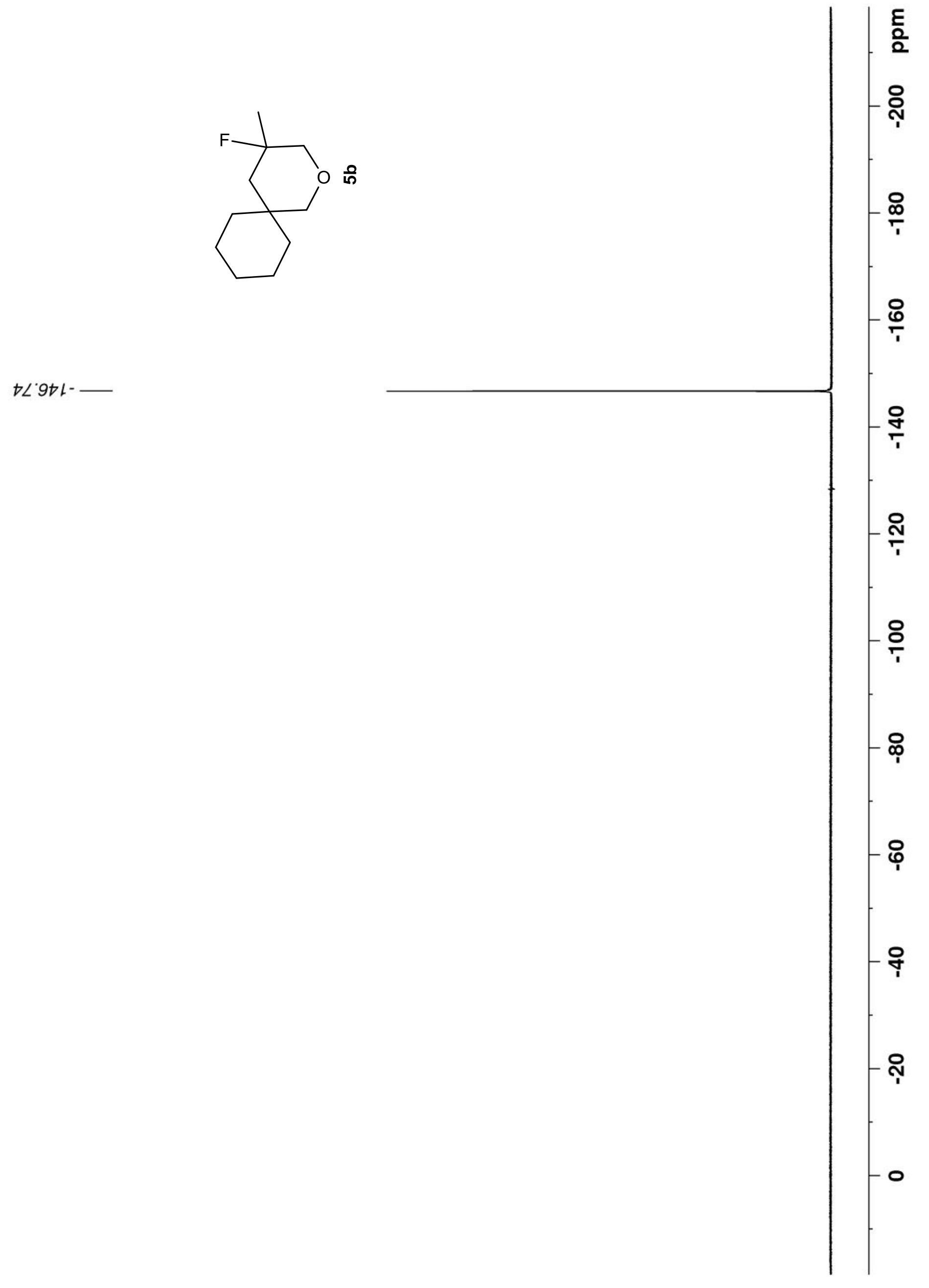
F
O
5b
-146.74
ppm
-200
-180
-160
-140
-120
-100
-80
-60
-40
-20
0

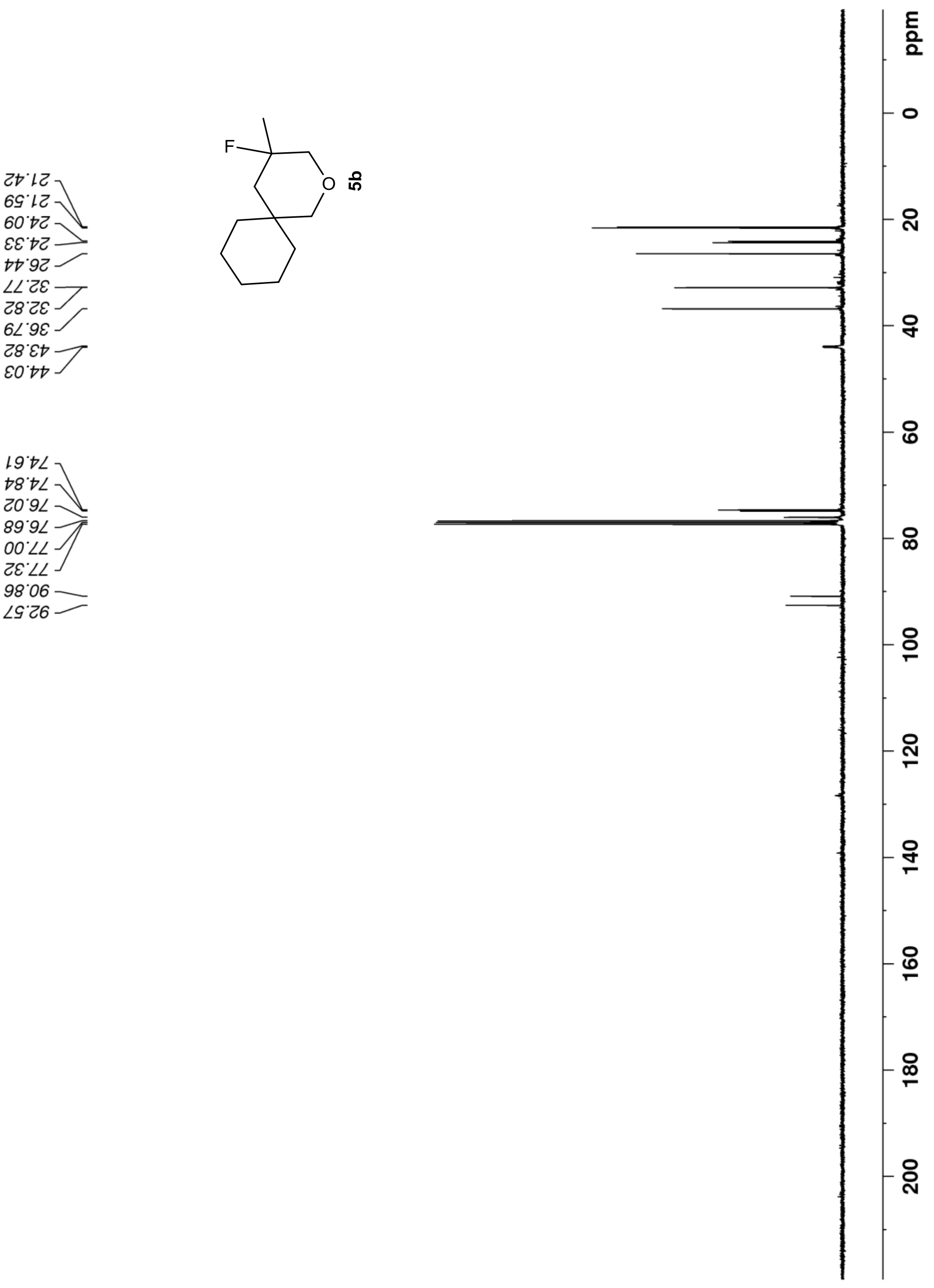

21.42
21.59
24.09
24.33
26.44
32.77
32.82
36.79
43.82
44.03
74.61
74.84
76.02
76.68
77.00
77.32
90.86
92.57
F
O
5b
ppm
0
20
40
60
80
100
120
140
160
180
200

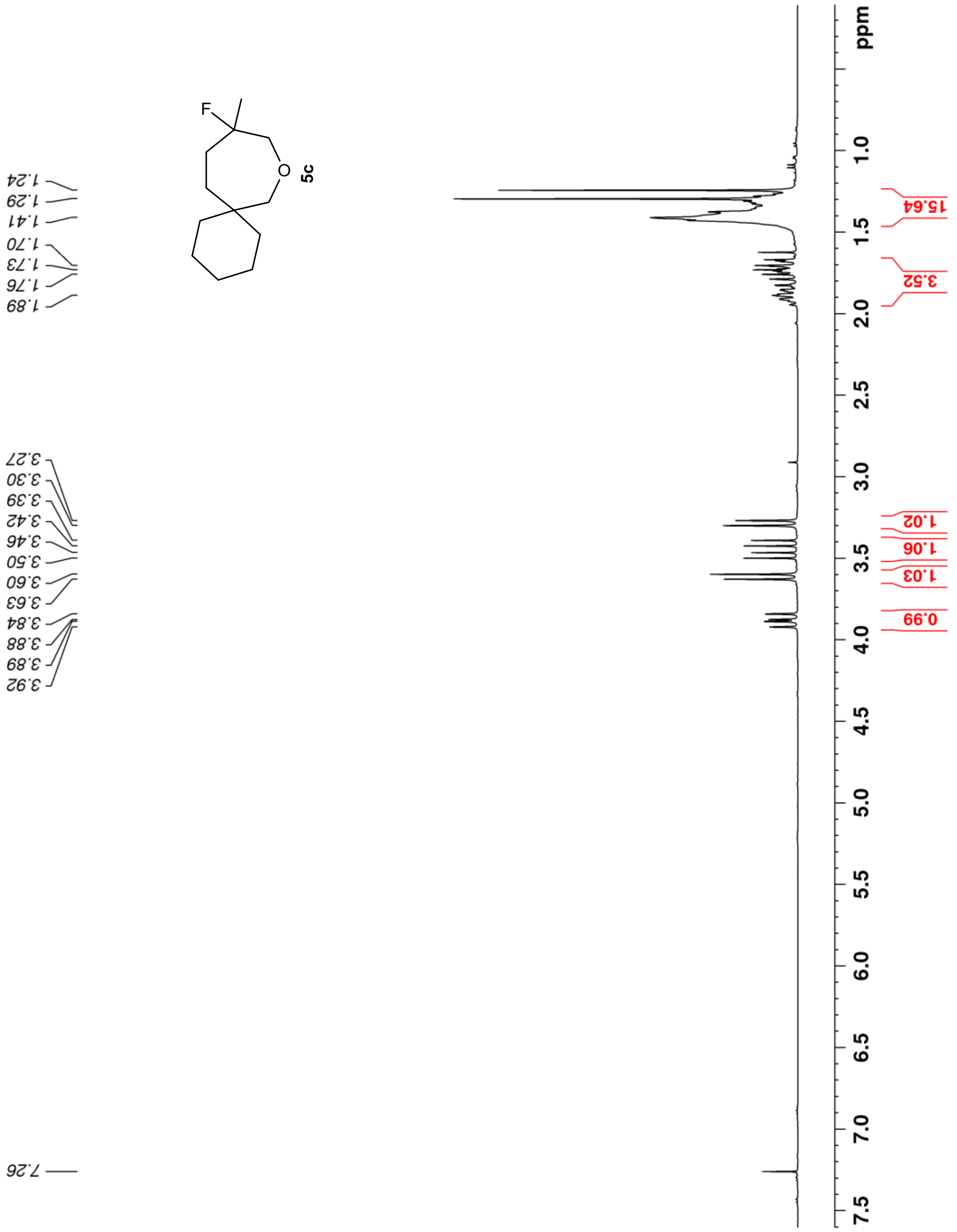
5c
F
O
1.24
1.29
1.41
1.70
1.73
1.76
1.89
3.27
3.30
3.39
3.42
3.46
3.50
3.60
3.63
3.84
3.88
3.89
3.92
7.26
15.64
3.52
1.02
1.06
1.03
0.99
ppm
1.0
1.5
2.0
2.5
3.0
3.5
4.0
4.5
5.0
5.5
6.0
6.5
7.0
7.5

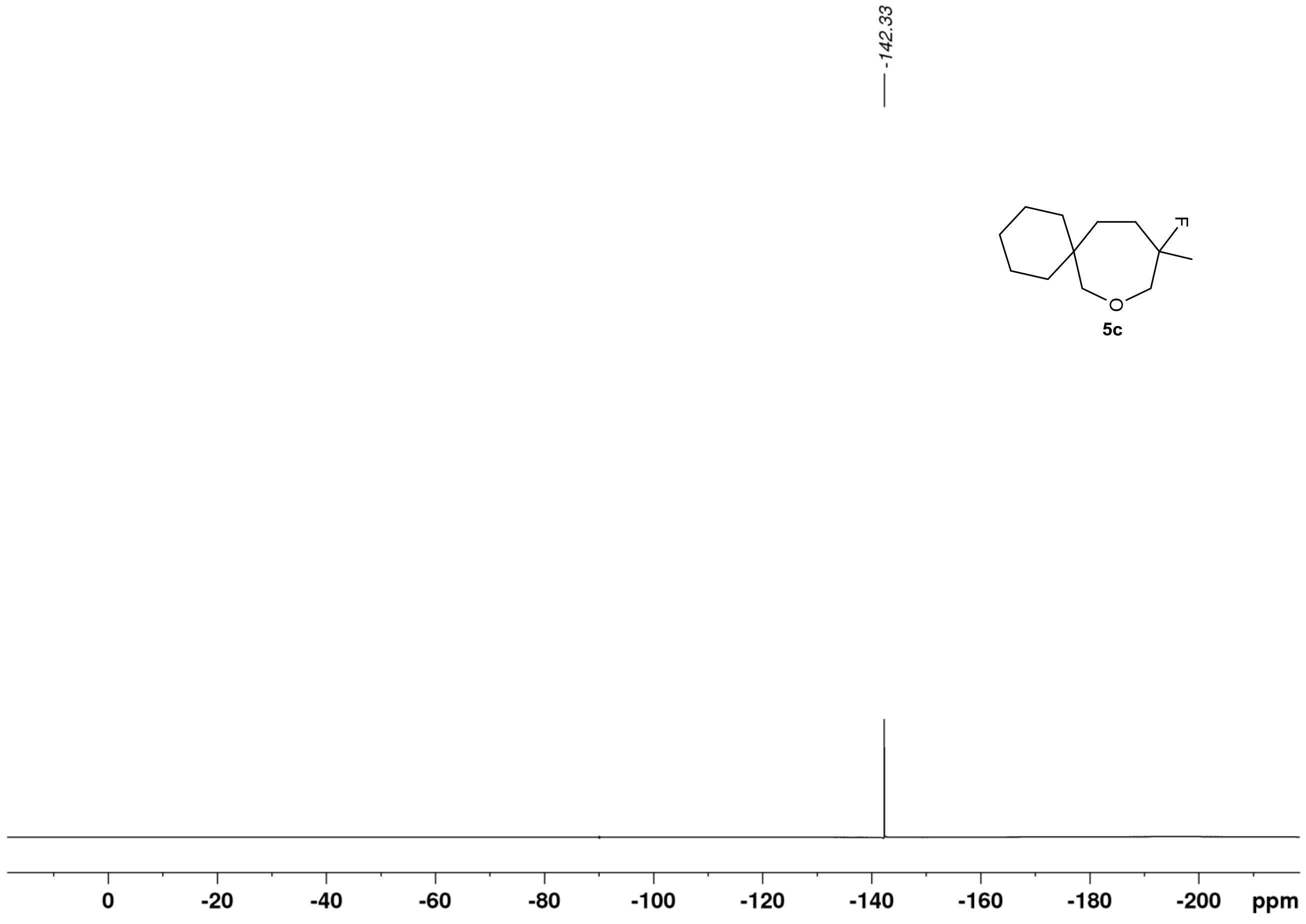
-142.33
5c
0
-20
-40
-60
-80
-100
-120
-140
-160
-180
-200
ppm

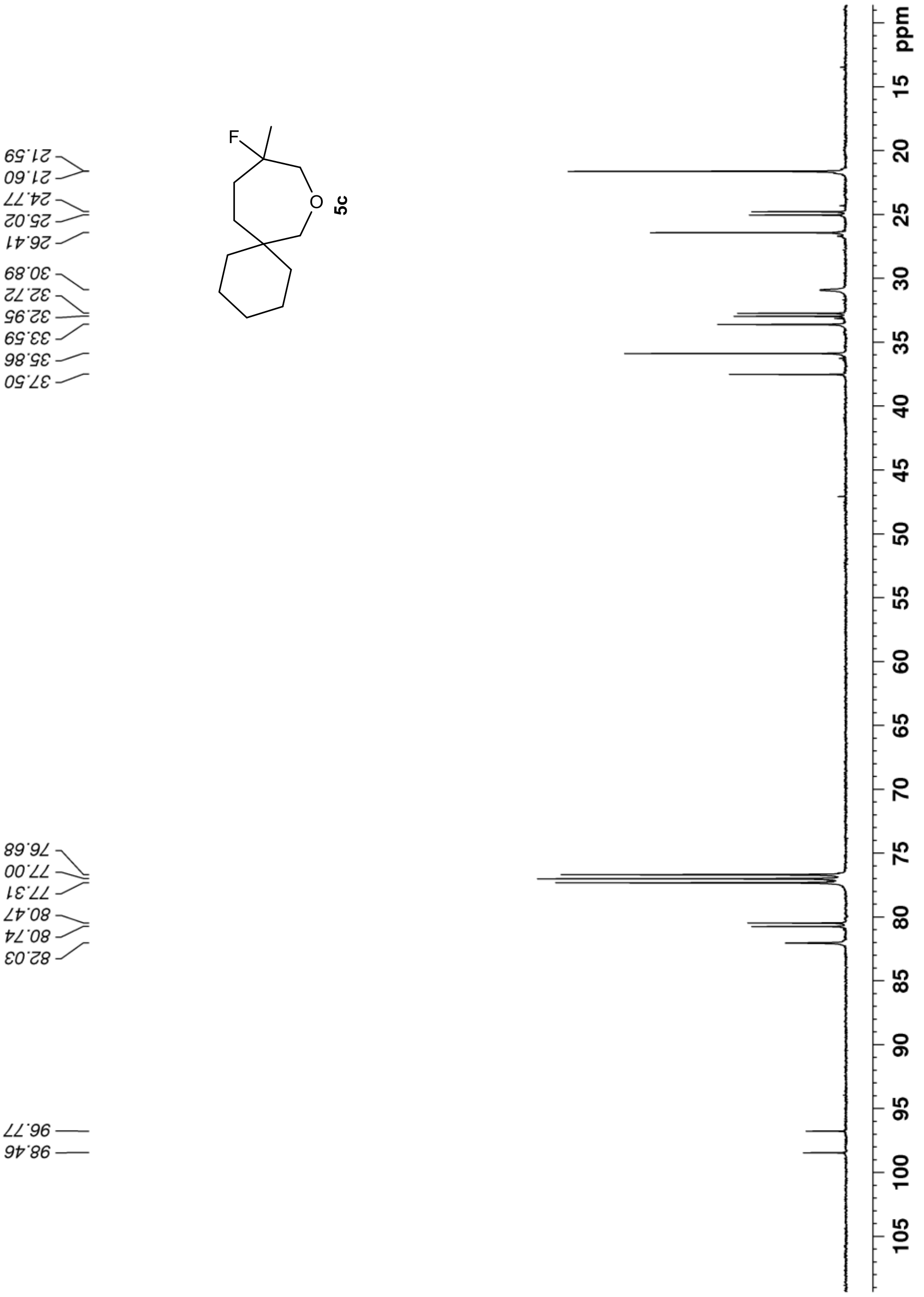
F
O
5c
21.59
21.60
24.77
25.02
26.41
30.89
32.72
32.95
33.59
35.86
37.50
76.68
77.00
77.31
80.47
80.74
82.03
96.77
98.46
ppm
15
20
25
30
35
40
45
50
55
60
65
70
75
80
85
90
95
100
105

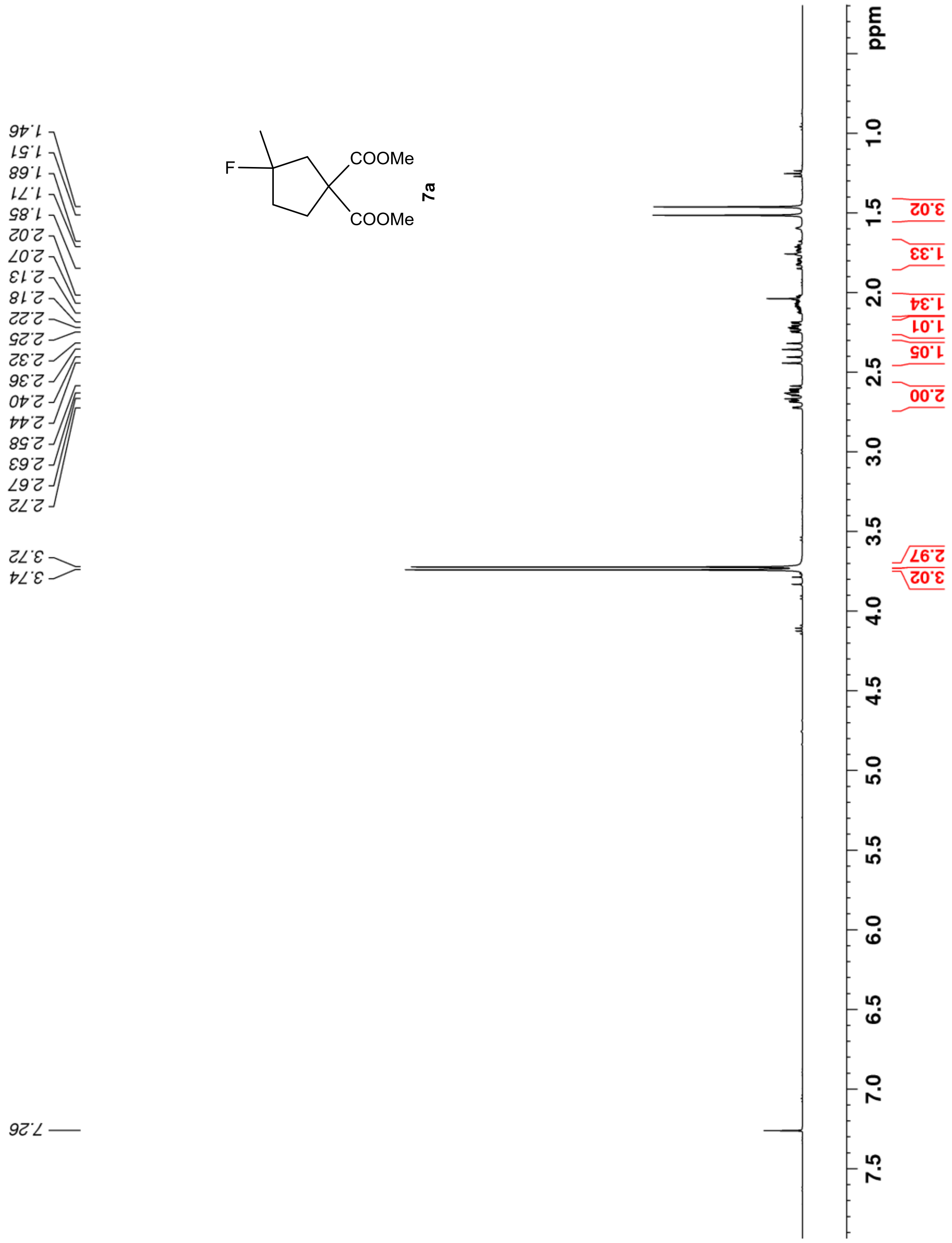
1.46
1.51
1.68
1.71
1.85
2.02
2.07
2.13
2.18
2.22
2.25
2.32
2.36
2.40
2.44
2.58
2.63
2.67
2.72
3.72
3.74
7.26
F
COOMe
COOMe
7a
ppm
3.02
1.33
1.34
1.01
1.05
2.00
2.97
3.02

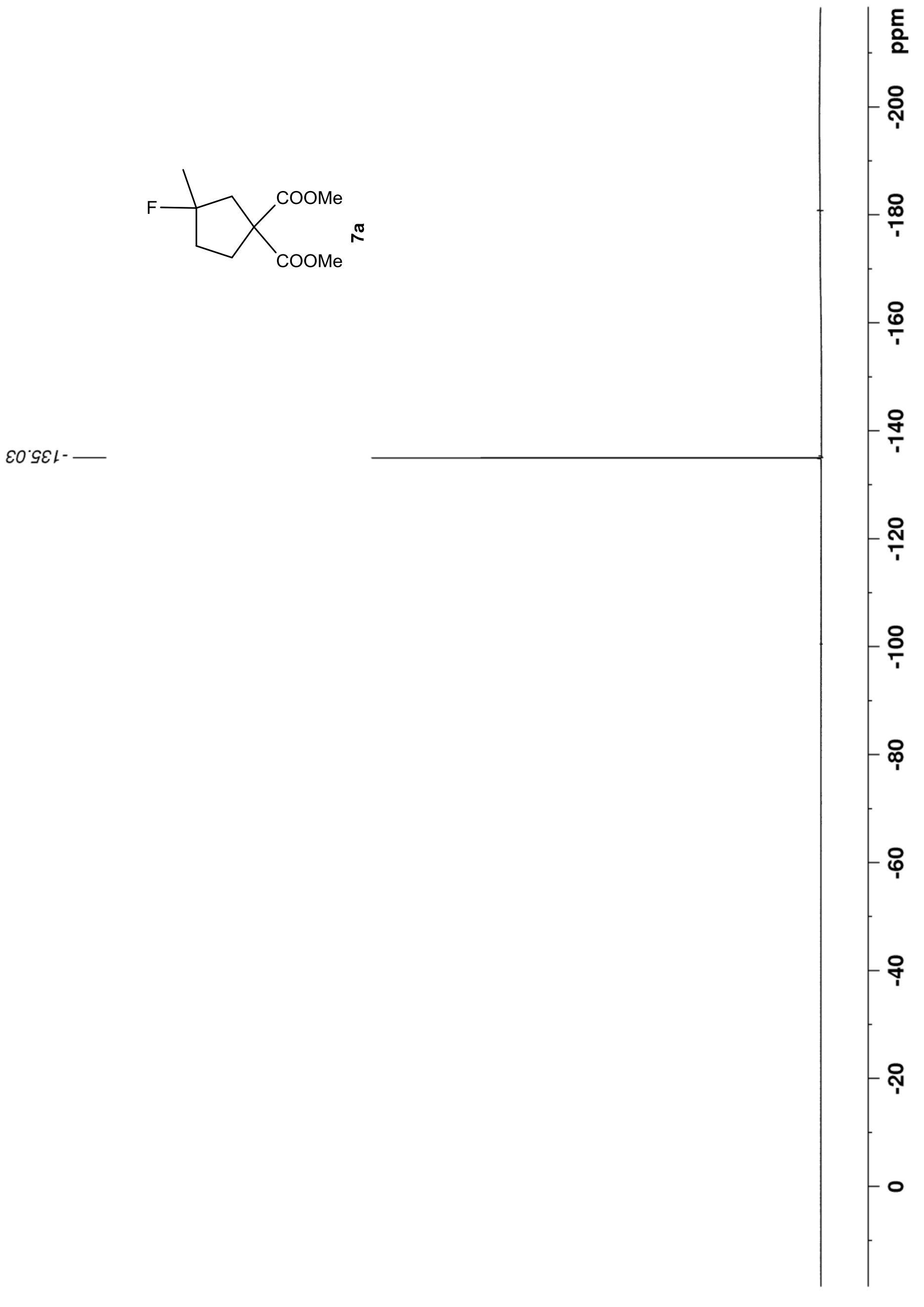

F
COOMe
COOMe
7a
-135.03
ppm
-200
-180
-160
-140
-120
-100
-80
-60
-40
-20
0

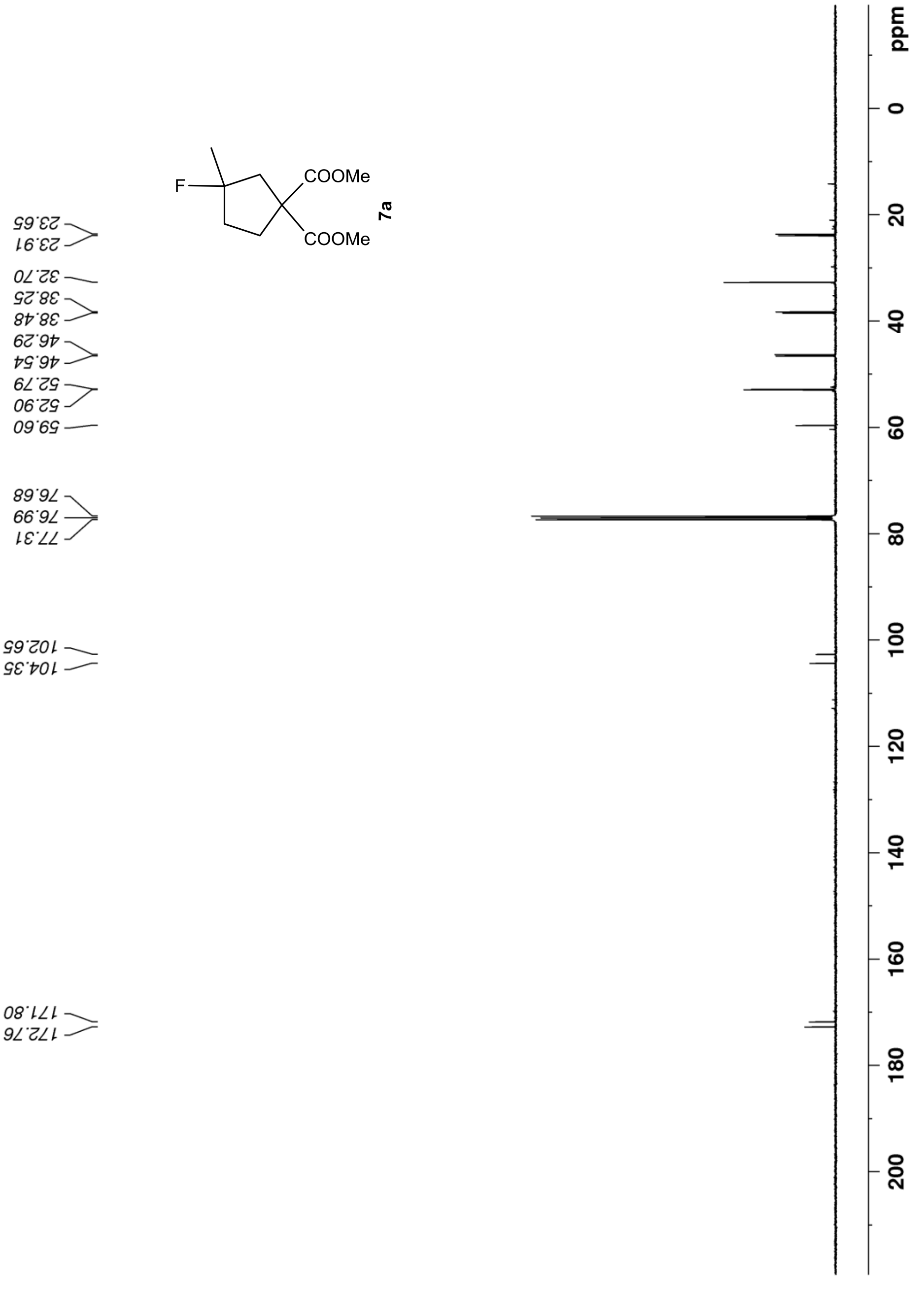
F
COOMe
COOMe
7a
23.65
23.91
32.70
38.25
38.48
46.29
46.54
52.79
52.90
59.60
76.68
76.99
77.31
102.65
104.35
171.80
172.76
ppm
0
20
40
60
80
100
120
140
160
180
200

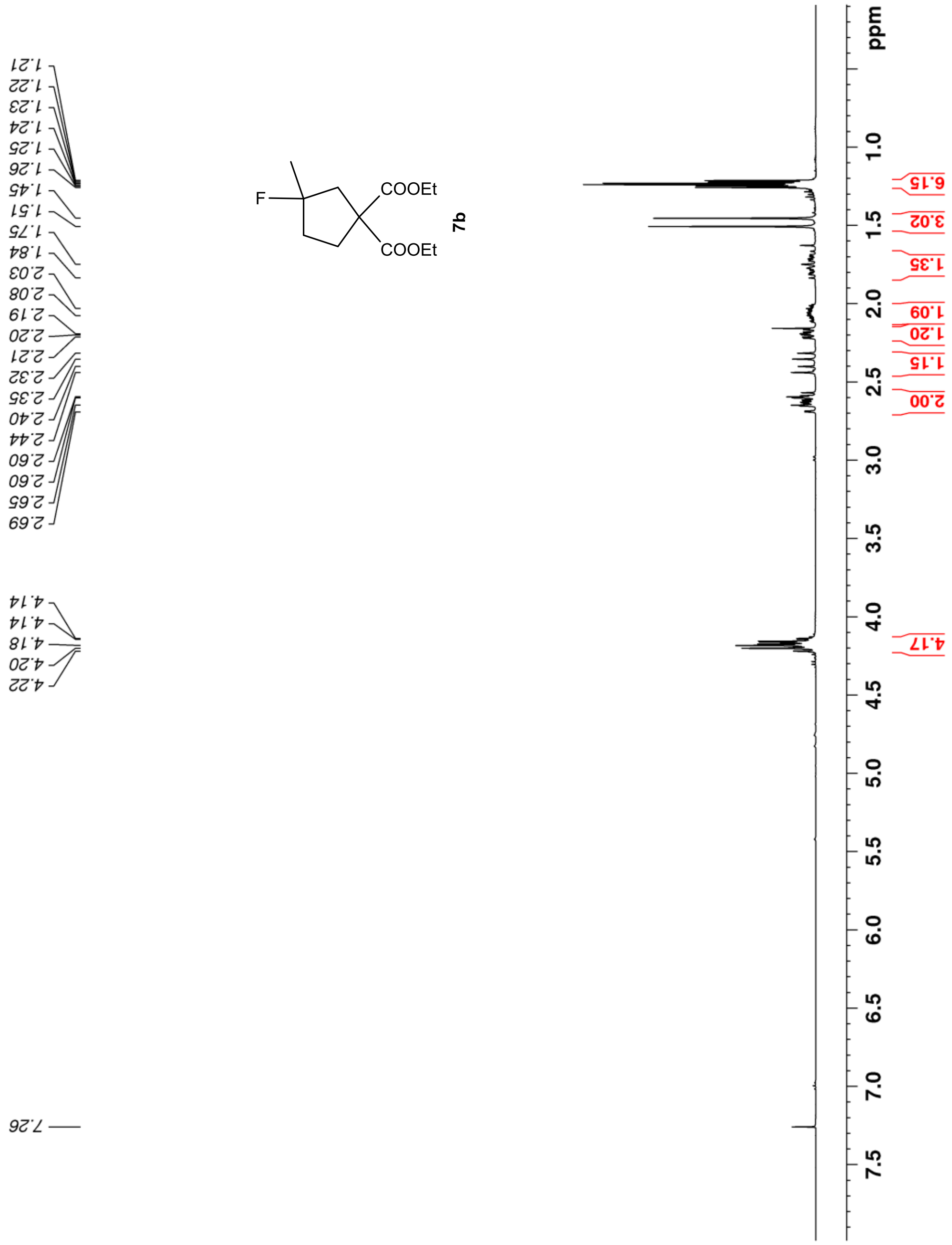
F
COOEt
COOEt
7b
ppm

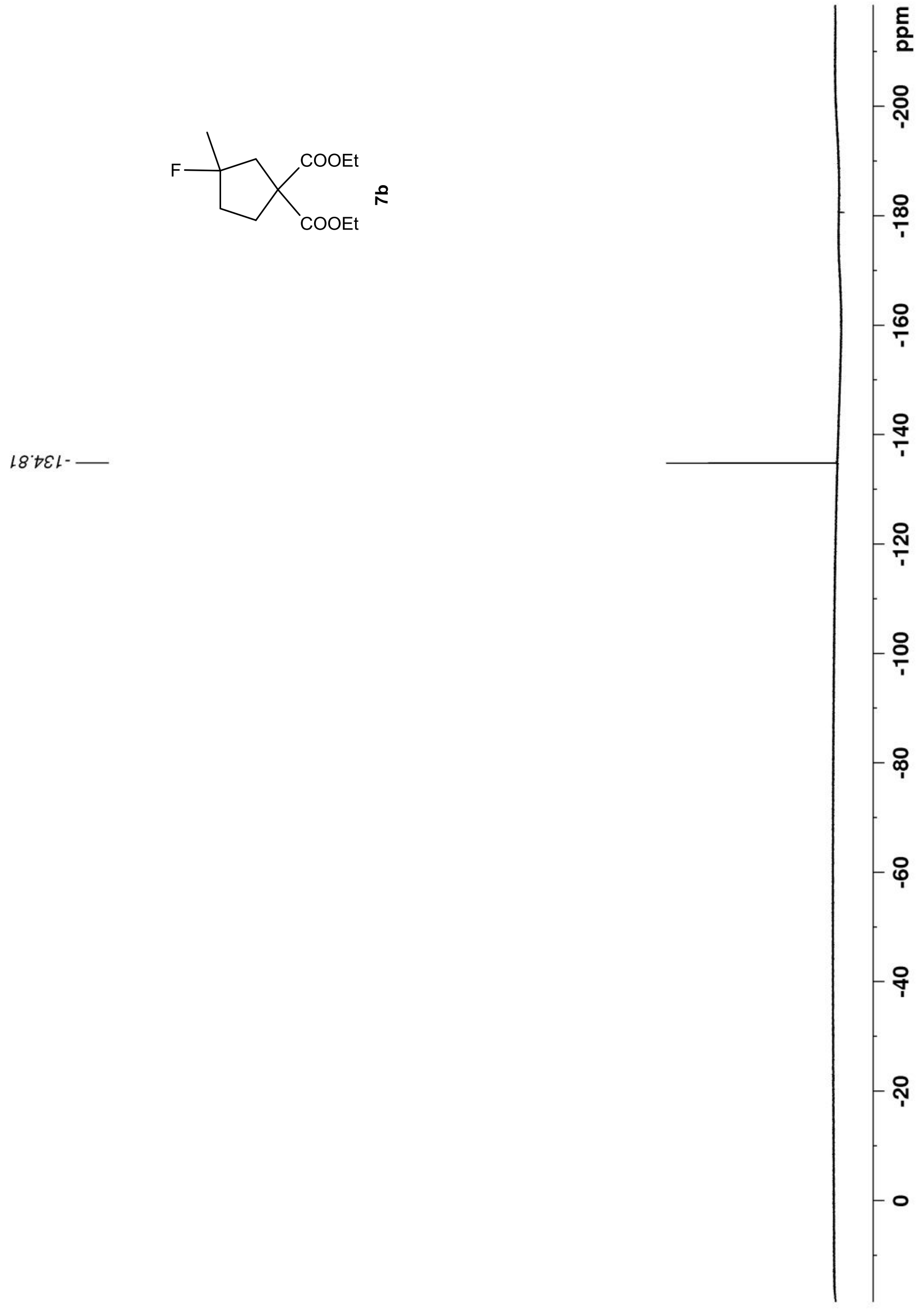

-134.81
F
COOEt
COOEt
7b
ppm
-200
-180
-160
-140
-120
-100
-80
-60
-40
-20
0

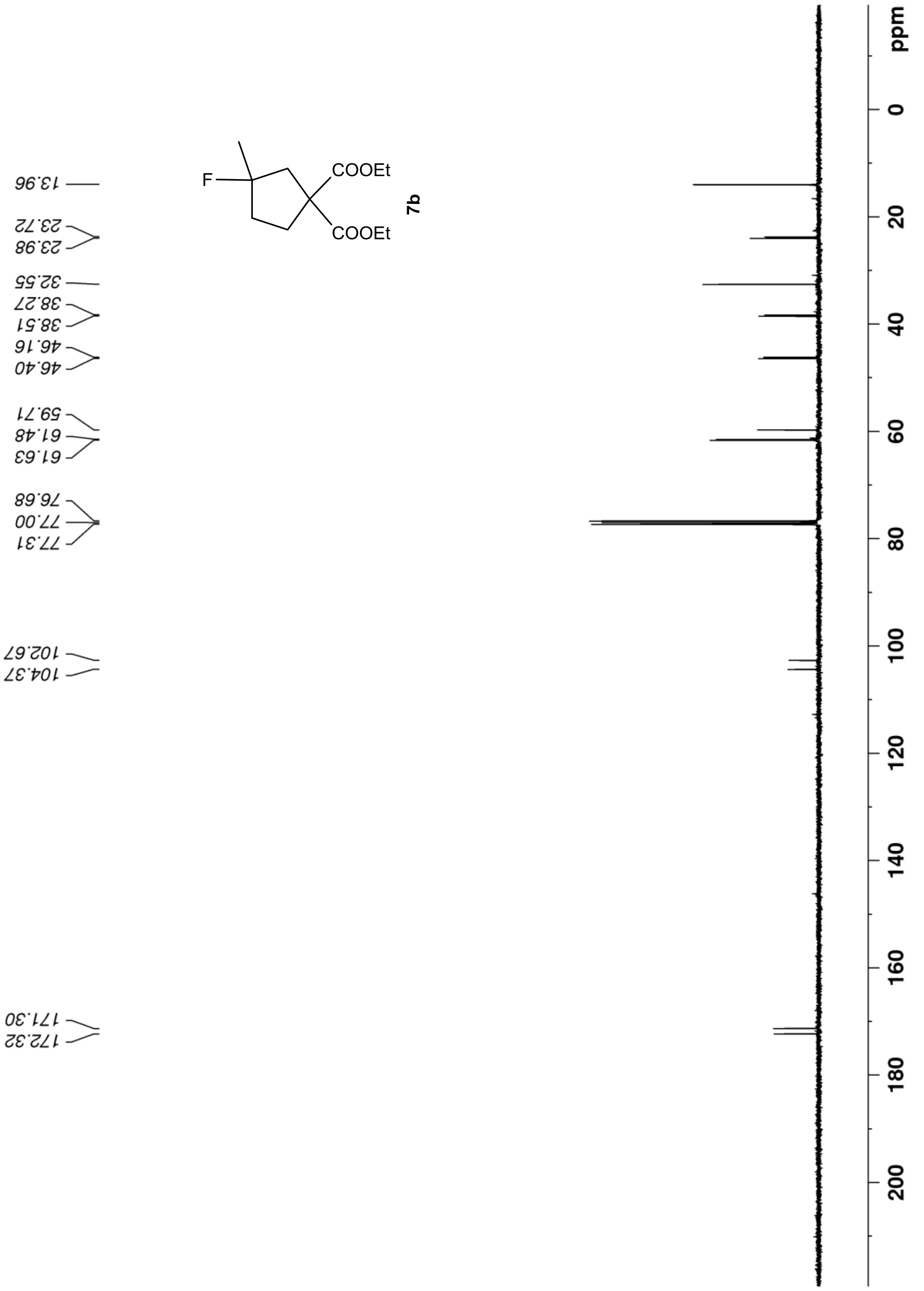

13.96
23.72
23.98
32.55
38.27
38.51
46.16
46.40
59.71
61.48
61.63
76.68
77.00
77.31
102.67
104.37
171.30
172.32
F
COOEt
COOEt
7b
ppm
0
20
40
60
80
100
120
140
160
180
200

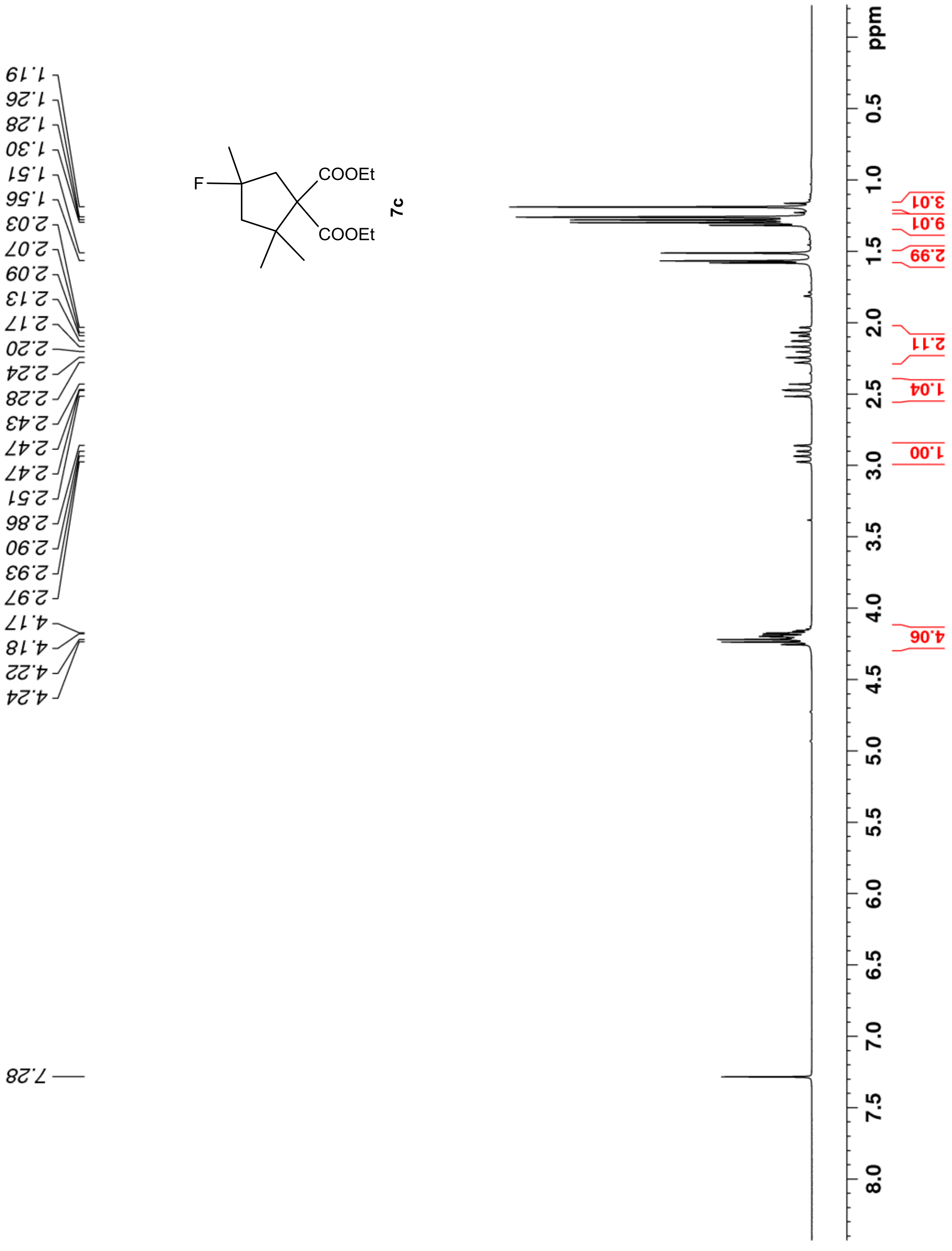
1.19
1.26
1.28
1.30
1.51
1.56
2.03
2.07
2.09
2.13
2.17
2.20
2.24
2.28
2.43
2.47
2.47
2.51
2.86
2.90
2.93
2.97
4.17
4.18
4.22
4.24
7.28
F
COOEt
COOEt
7c
3.01
9.01
2.99
2.11
1.04
1.00
4.06
ppm

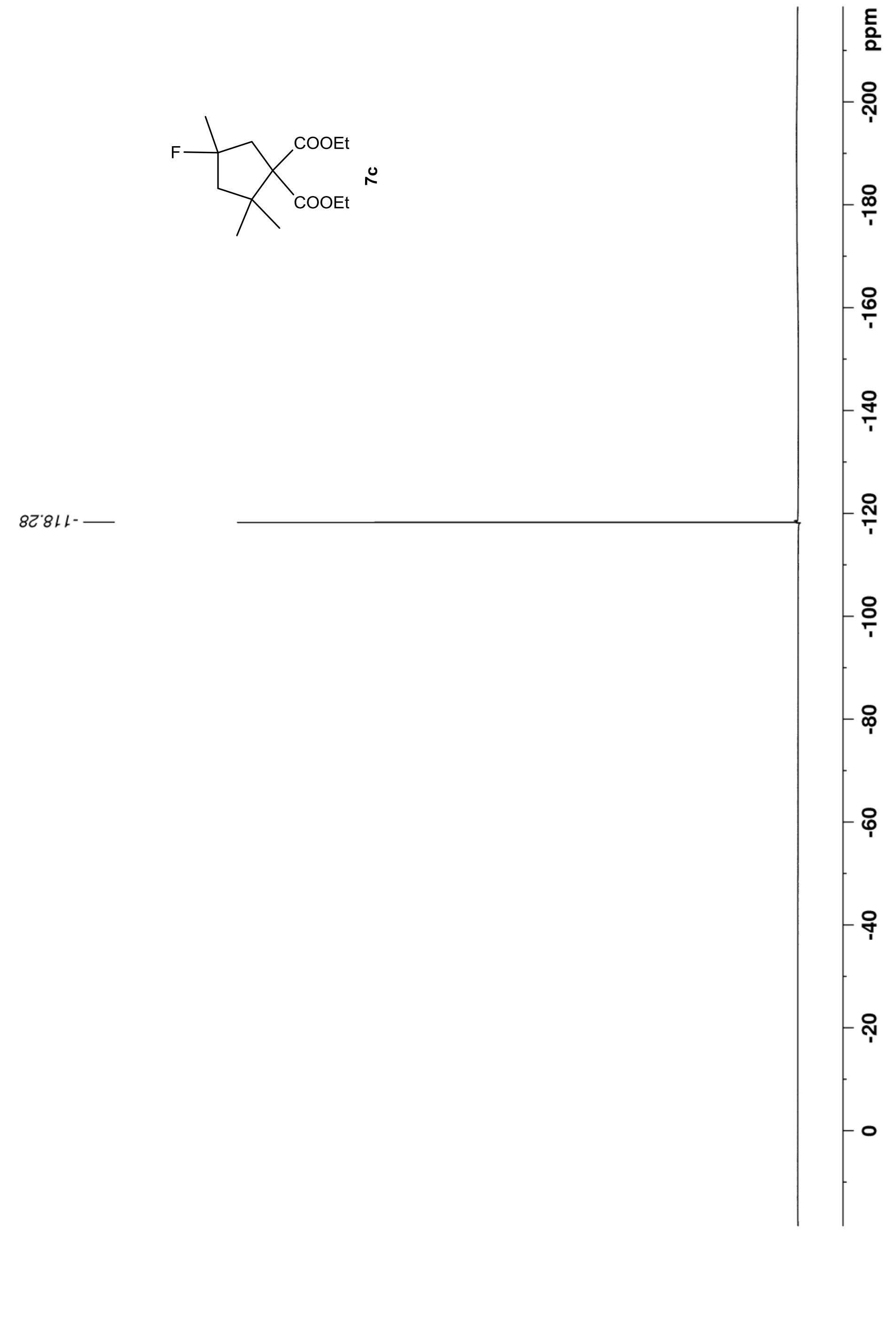

-118.28
F
COOEt
COOEt
7c
ppm
-200
-180
-160
-140
-120
-100
-80
-60
-40
-20
0

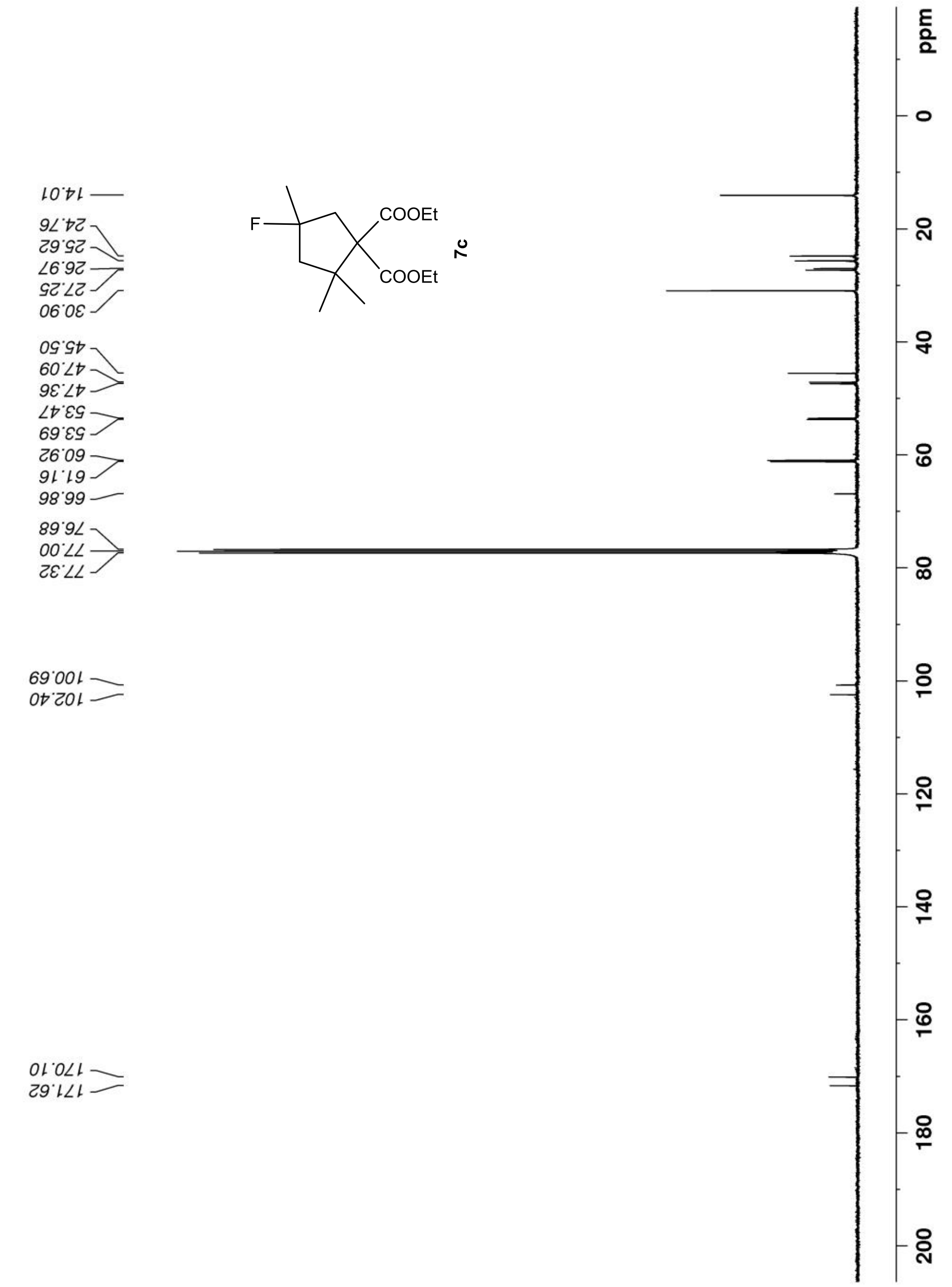
14.01
24.76
25.62
26.97
27.25
30.90
45.50
47.09
47.36
53.47
53.69
60.92
61.16
66.86
76.68
77.00
77.32
100.69
102.40
170.10
171.62
F
COOEt
COOEt
7c
ppm
0
20
40
60
80
100
120
140
160
180
200

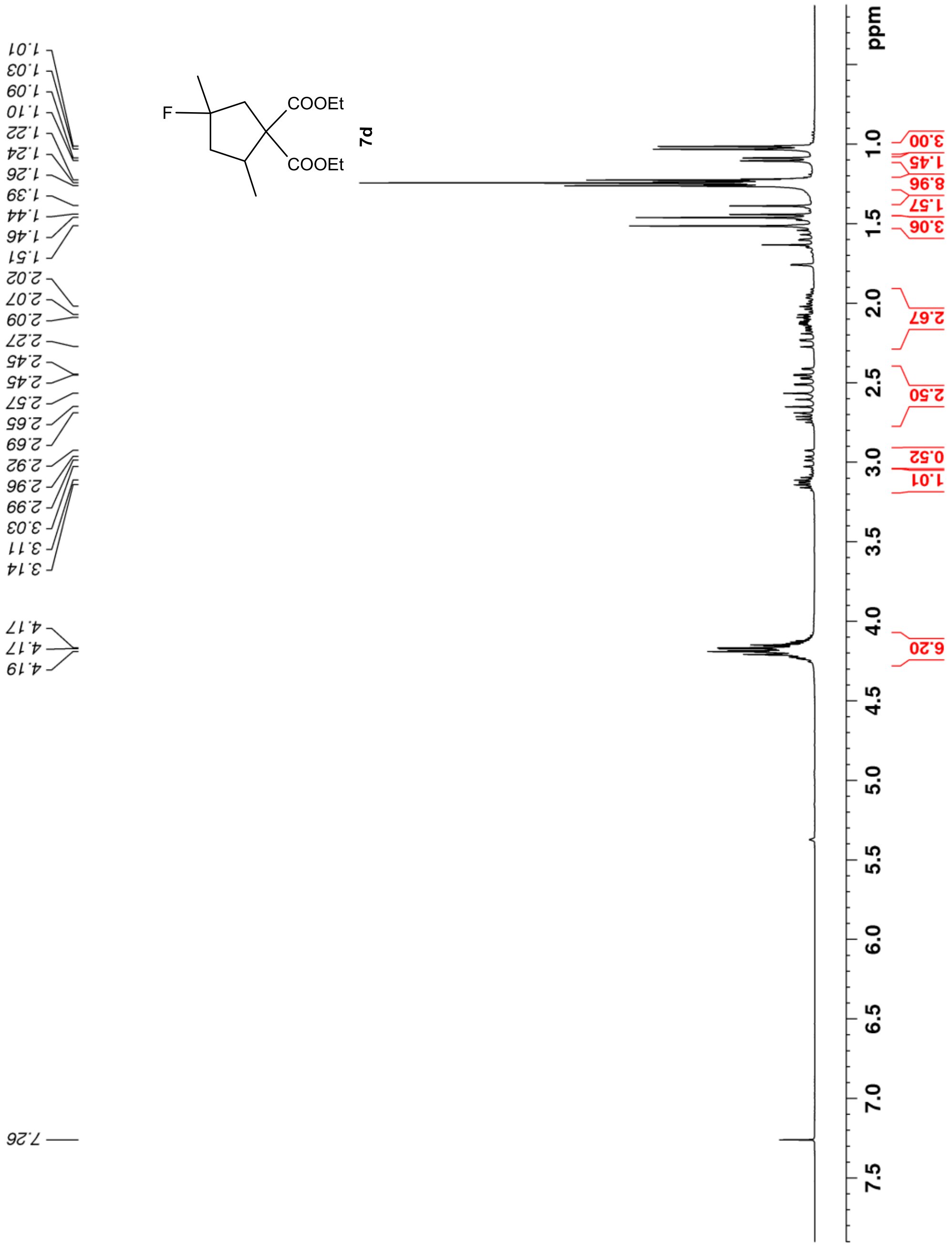

F
COOEt
COOEt
7d
1.01
1.03
1.09
1.10
1.22
1.24
1.26
1.39
1.44
1.46
1.51
2.02
2.07
2.09
2.27
2.45
2.45
2.57
2.65
2.69
2.92
2.96
2.99
3.03
3.11
3.14
4.17
4.17
4.19
7.26
3.00
1.45
8.96
1.57
3.06
2.67
2.50
0.52
1.01
6.20
ppm
1.0
1.5
2.0
2.5
3.0
3.5
4.0
4.5
5.0
5.5
6.0
6.5
7.0
7.5

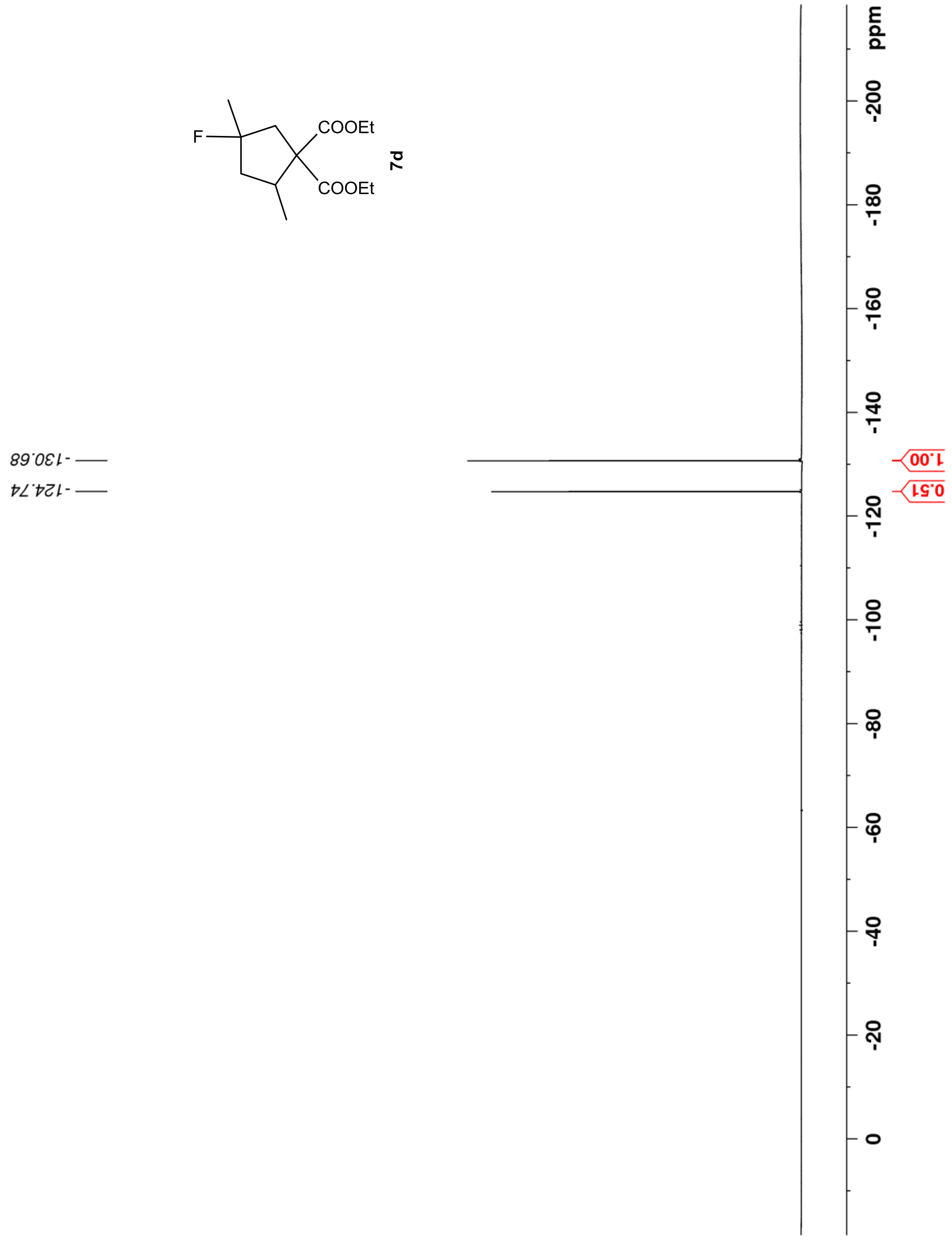
F
COOEt
COOEt
7d
-130.68
-124.74
1.00
0.51
ppm
-200
-180
-160
-140
-120
-100
-80
-60
-40
-20
0

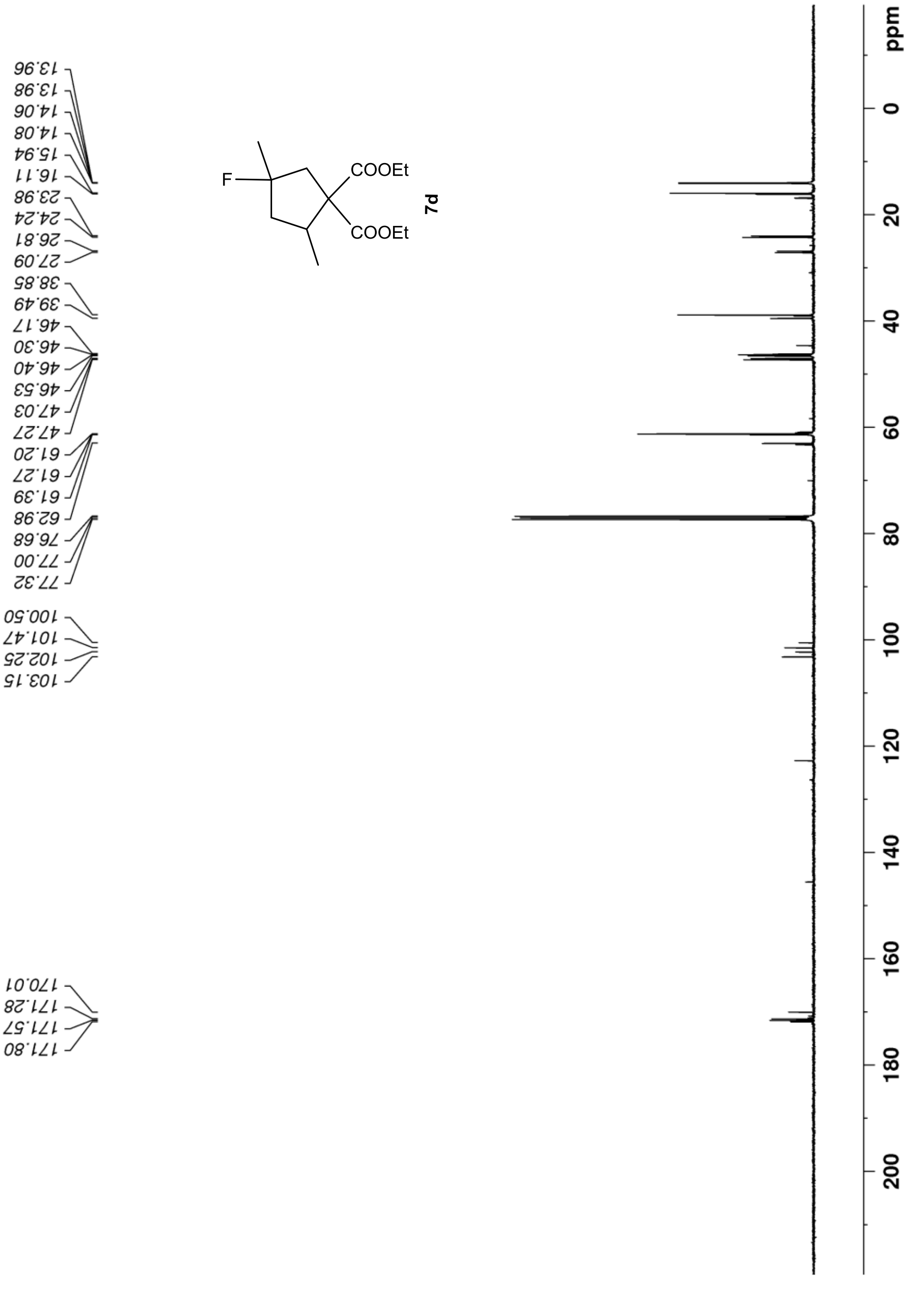
13.96
13.98
14.06
14.08
15.94
16.11
23.98
24.24
26.81
27.09
38.85
39.49
46.17
46.30
46.40
46.53
47.03
47.27
61.20
61.27
61.39
62.98
76.68
77.00
77.32
100.50
101.47
102.25
103.15
170.01
171.28
171.57
171.80
F
COOEt
COOEt
7d
ppm
0
20
40
60
80
100
120
140
160
180
200
